# Transformation of the Phosphorus Atom in Hexacyclic Polyaromates and Its Impact on Physicochemical Properties

**DOI:** 10.1021/acs.joc.6c00184

**Published:** 2026-05-13

**Authors:** Eliška Mizerová, Joaquín Almarza González, Vladimír Církva, Jaroslav Žádný, Jan Storch, Martin Jakubec, Tomáš Beránek

**Affiliations:** † 86876 Institute of Chemical Process Fundamentals of the Czech Academy of Sciences, Rozvojová 1/135, 165 00 Prague 6, Czech Republic; ‡ Department of Organic Chemistry, University of Chemistry and Technology Prague, Technická 5, 166 28 Prague 6, Czech Republic

## Abstract

A series of eight phenanthrene-based phosphorus heterocycles were prepared to evaluate the effect of phosphorus-center modification on the optical and electrochemical properties of π-conjugated systems. A versatile synthetic route provided access to λ^3^-, λ^4^-, and λ^5^-phosphorus derivatives, including phosphinine, phosphine oxide, sulfide, selenide, phosphinic acid, amide, and phosphonium species. The resulting compounds were investigated using DFT calculations in combination with absorption and emission spectroscopy as well as cyclic voltammetry (CV) and differential pulse voltammetry (DPV). The data show that phosphinic acid derivatives exhibit only minor changes in optical and redox properties, whereas chalcogen exchange (O, S, and Se) has a more pronounced effect on the optical behavior. All compounds display irreversible oxidation waves above 0 V. Comparison with carbo and aza analogues revealed that the incorporation of phosphorus into the phenanthrene scaffold leads to a bathochromic shift in both absorption and emission. In contrast, the first reduction potentials (*E*_red1_) remain comparable across the series. Overall, these results provide a systematic overview of the influence of the phosphorus-center modification on the electronic properties of phenanthrene-based systems.

## Graphical abstract

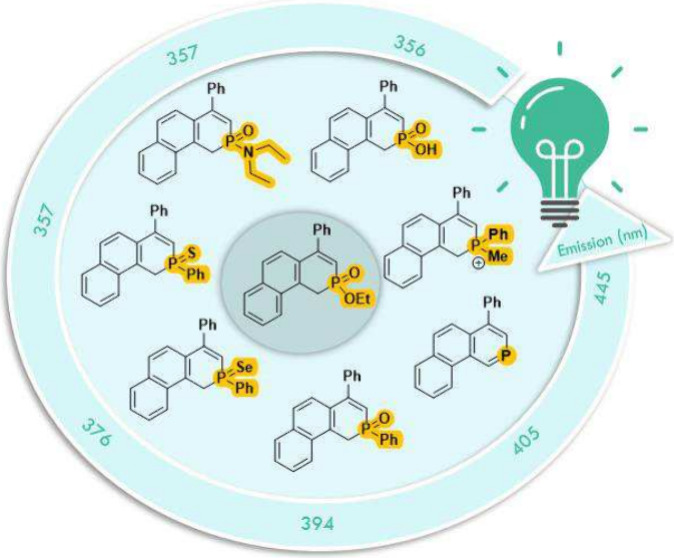

## Introduction

Phosphorus-enriched polycyclic aromatic π-systems have recently attracted a considerable amount of attention across various fields, such as bioimaging,^1^ cancer treatment,^2^ and material sciences.^3^ Compared with all-carbon analogues, introducing a phosphorus atom into the π-system offers the possibility of further functionalization of the target molecule. The phosphorus atom can be derivatized (e.g., through oxidation, reduction, or complex formation), which significantly influences some key characteristics of the entire system, such as the HOMO/LUMO gap, rate of charge transfer, triplet–triplet spin interaction, and absorption and luminescence properties.^4,5^ This facilitates the generation of a broad spectrum of derivatives, each exhibiting distinct properties, from a single initial structure.^3^ While phospholes, the five-membered phosphorus rings, have previously been utilized in organophosphorus chemistry, driving advances in various optoelectronic materials,^6,7^ π-systems based on six-membered phosphorus heterocycles have gained a significant amount of attention only recently.^8–12^ A phosphorus atom in these molecules can significantly enhance both the solubility and the chemical stability,^3^ providing ambipolar redox properties,^13,14^ and improves electroluminescence^15–17^ and, in some cases, increases quantum yields,^18^ paving the way for development in organic light-emitting diodes (OLEDs),^19–22^ organic field-effect transistors (OFETs),^23^ sensors,^24–26^ redox-active chromophores,^27^ and electrofluorochromic devices (EFs).^28^ Along with tuning optoelectronic properties, six-membered phosphorus heterocycles significantly modulate the molecular packing and spatial arrangement,^29^ which should be considered as a valuable factor in crystallographic engineering and accessing the application of these organic materials.^30,31^

The phosphorus derivatization approach provides a versatile means of tuning the photophysical properties, as demonstrated by various groups (Figure 1). Dibenzophosphindolizine derivatives prepared by Kamikawa^32^ showed changes in absorption maxima and molar absorption coefficients depending on the chalcogen atom. Romero-Nieto’s group has demonstrated that various λ^5^- and λ^4^-phosphorus derivatives of one initial structure can induce pronounced bathochromic emission shifts of up to 67 nm.^29^ This approach was also confirmed by Kivala’s study of phosphorus-containing dibenzonaphthanthrenes.^33^ Ren^34^ introduced a series of new (diaza)­phosphinines connected with fluorenes, and it was shown that the introduction of a phosphorus atom red-shifts the absorption spectra more than 100 nm when compared to those of a non-phosphorus derivative. A few other groups took advantage of the ability of phosphorus to red-shift the emission spectra of the molecules, and a series of fluorescent compounds containing a six-membered phosphorus ring were introduced.^1,15–17,25–27,35–37^

**Figure 1:**
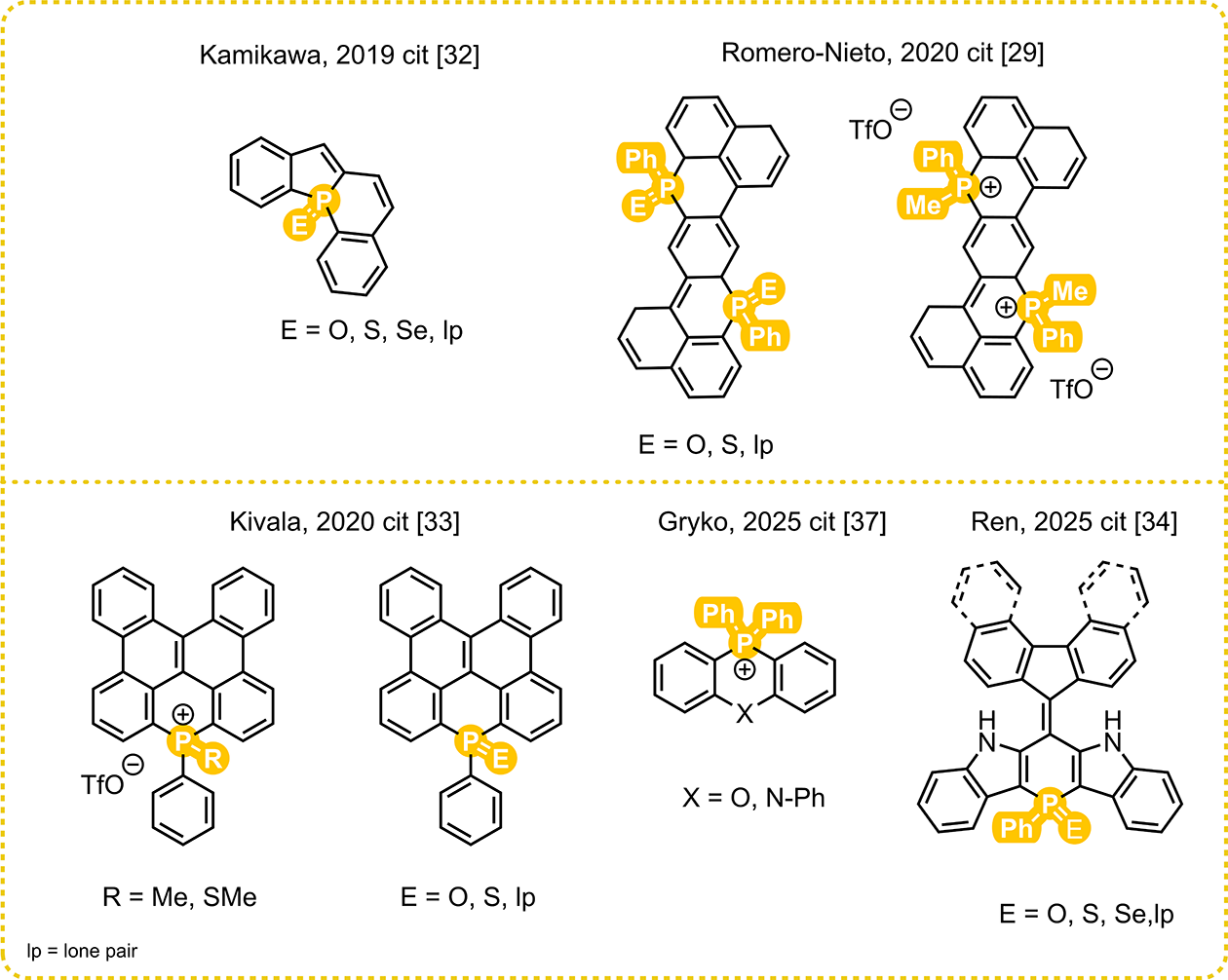
Selected examples of six-membered phosphorus ring derivatizations.

Despite the growing interest in this field, systematic and comprehensive studies addressing the impact of specific phosphorus derivatives on the properties of the investigated systems remain scarce. Until recently, this limitation was largely due to the lack of general synthetic methodologies enabling access to six-membered phosphorus-containing heterocycles.^12,38–43^ Our group has recently reported a robust synthetic approach allowing the incorporation of a phosphinine ring into an aromatic framework,^9,11^ as well as the preparation of quinolizidine-based 1,4-azaphosphinines from alkyne precursors.^44^ Building upon these findings, the present study describes an efficient and versatile strategy for the synthesis of λ^3^-, λ^4^-, and λ^5^-phosphorus derivatives from a common starting material, enabling access to a significantly broader range of phosphorus species than that typically achieved with phosphine chalcogenides. The optoelectronic properties of prepared compounds are studied and discussed. In some cases, the comparison with carbo and aza analogues is provided. We believe that the obtained data can serve as a starting point for synthesizing a new class of compounds with the potential to advance functional materials.

## Results and Discussion

### Synthesis

To investigate how the incorporation of different phosphorus moieties influences the optical characteristics, redox behavior, and overall photophysical response of the system, we prepared a small library of phosphorus derivatives based on the dihydro-phosphaphenanthrene framework. We have recently published a simple and straightforward synthesis of phosphinate **2**,^11^ which was identified as a suitable starting material not only because it is a convenient precursor for our proposed synthetic transformations (Scheme 1) but also because we previously observed an increased the stability of some phosphorus derivatives when incorporated into larger π-systems.

**Scheme 1:**
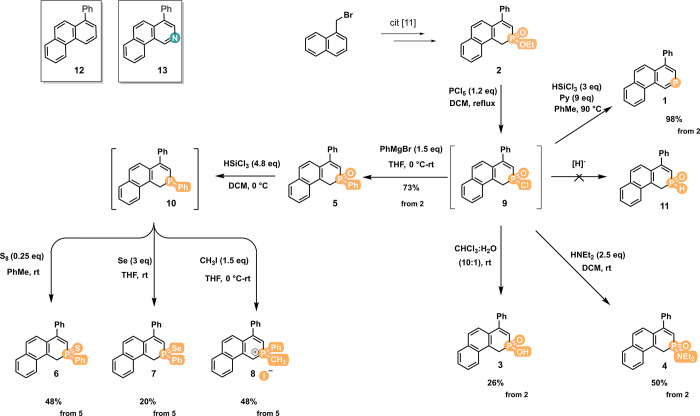
Synthesis of Phosphaphenanthrene Derivatives **1**–**8**

The first step in transforming phosphinate **2** was to sufficiently increase its reactivity since **2** itself was found to be poorly reactive in most of the transformations, such as hydrolysis, aminolysis, or direct substitution with organolithium reagents. Therefore, **2** was converted *in situ* into a more reactive phosphinic chloride **9**, which was subsequently used for the synthesis of other phosphorus derivatives (Scheme 1). The preparation of phosphinine **1** from **2** via chloride **9** was performed according to our previously reported procedure^11^ by reduction of the phosphine oxide group by trichlorosilane (HSiCl_3_), which resulted in the formation of fully aromatized product **1** in nearly quantitative yield.

Phosphinic acid **3** was obtained by the hydrolysis of chloride **9** in 26% yield from **2**. Although the conversion of the reaction was complete, the purification of acid **3** proved to be difficult, thereby significantly reducing the yield. Similarly, the reaction of chloride **9** with diethylamine resulted in the formation of amide **4**, with a yield of 50%.

In principle, the reduction of chloride **9** with a hydride reagent should provide secondary phosphine oxide **11**. However, all attempts to achieve this reaction were unsuccessful, resulting in a complex reaction mixture. The preparation of phenyl phosphine oxide **5** was performed from **2** via **9** by substitution with phenyl magnesium bromide in 73% overall yield. Phosphine oxide **5** can also function as a versatile precursor for further manipulation of phosphorus, in terms of both its oxidation state and substitution. The reduction of **5** by HSiCl_3_ leads to air-sensitive phosphine **10**. This compound is prone to oxidation with various oxidants; therefore, the immediate reaction with elemental sulfur or selenium provides sulfides **6** and selenide **7** in 48% and 20% yields, respectively. Additionally, phosphines can be utilized in the synthesis of phosphonium salts. The methylation of **10** by methyl iodide produced phosphonium iodide **8** in a total yield of 37%.

A majority of the prepared phosphorus derivatives were sufficiently stable to undergo purification by flash chromatography on silica gel. The exceptions in this case are methyl phosphonium iodide **8**, which was obtained by crystallization from a mixture of dichloromethane and heptane, and phosphinic acid **3**, which was isolated by crystallization from methanol. Phosphinine **1** decomposes on silica gel and, therefore, was purified by filtration through a short plug of alumina. In addition to the series of phosphaphenanthrene derivative **1**, we also prepared its carbo (**12**) and aza (**13**) analogues for direct comparison of the impact of the heteroatom on the physicochemical properties of the phenanthrene moiety. Aza-phenanthrene **13** was synthesized following a reported procedure,^45^ while purely hydrocarbon analogue **12** was obtained through UV-assisted cyclization of the corresponding stilbene (see the Supporting Information (SI) for details).

### Optical Properties

The absorption and emission spectra of phosphorus-derived phenanthrenes are summarized in Table 1 and Figure 2. Introduction of a phosphorus atom into the phenanthrene scaffold induces a pronounced bathochromic shift. Carbocyclic analogue **12** absorbs at 300 nm and aza analogue **13** at 305 nm, whereas phosphinine **1** absorbs at 327 nm (Figure 2A). The emission spectra follow the same trend. While the carbocyclic and nitrogen analogues emit at 357 and 355 nm, respectively, phosphinine **1** exhibits an emission maximum at 404 nm (Figure 2A).

**Figure 2:**
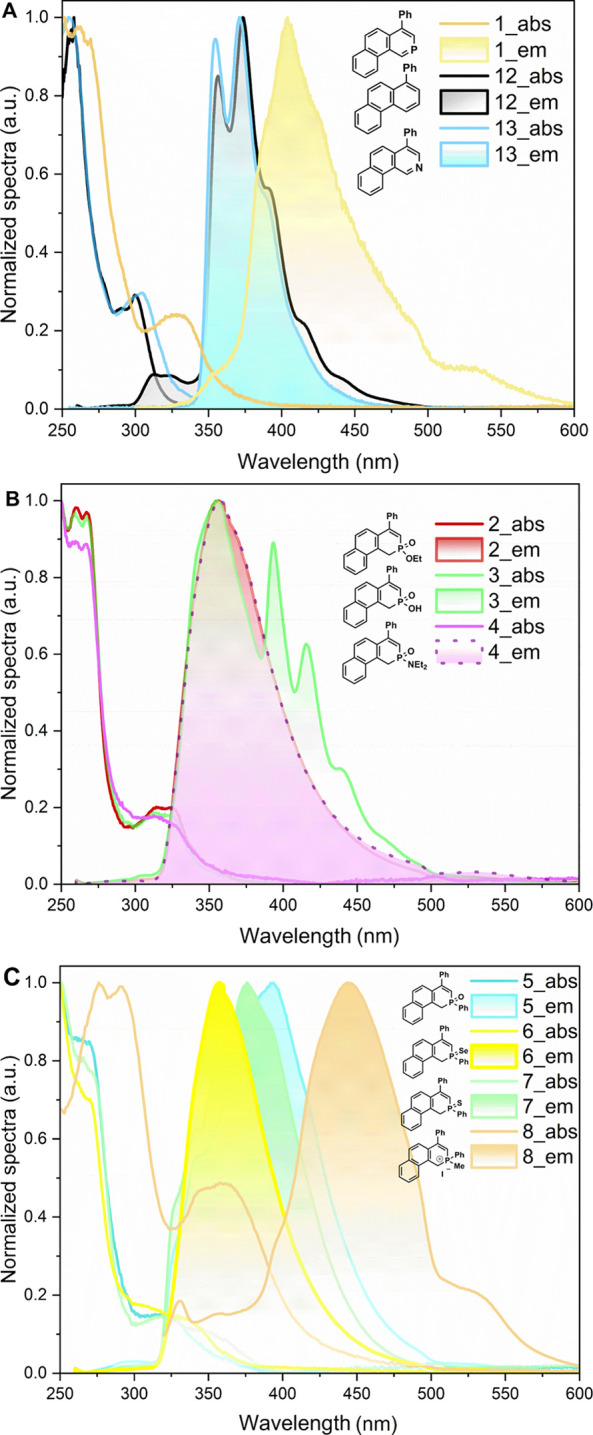
Normalized absorption and emission spectra in a DCM solution (*c*_abs_ = 1 × 10^–5^ M; *c*_em_ = 1 × 10^–6^ M) of (A) **1**, **12**, and **13** and (B) **2**–**4**, and (C) **5**–**8**.

**Table 1:** UV–Vis and Fluorescence Data of Compounds **1**–**8**, **12**, and **13**

compound	*λ*_max_^abs^ (nm) ( E (M^–1^ cm^–1^))^a^	*E*_g_^opt^ (eV)	*λ*_max_^em^ (nm)^b^
**1**	261 (24 665), 327 (6118)	3.49	404^c^
**2**	260 (26 795), 315 (5484)	3.51	357
**3**	260 (30 692), 314 (5914)	3.50	356, 394,^d^ 417,^d^ 441^d^
**4**	260 (28 715), 313 (5721)	3.46	357
**5**	266 (21 750), 319 (3826)	3.46	344, 394
**6**	270 (29 037), 330 (6158)	3.32	357
**7**	266 (26 666), 344 (3714)	3.16	376, 393
**8**	276 (25 112), 292 (24 868), 346 (11 845), 361 (12 217)	2.37	445
**12**	258 (19 571), 300 (5229)	3.86	357, 374
**13**	255 (40 342), 305 (11 952)	3.78	355, 372

a Absorption maxima in a DCM solution (1 × 10^–5^ M).

b Emission maxima in a DCM solution (1 × 10^–6^ M, excitation at 240 nm).

c Excitation at 240 nm. The underlined values signify the most intensive absorption/emission band.

d Emission maxima attributed to H-bonded dimers and higher-order aggregates.

Phosphorus derivatives **2** and **4**, derived from phosphinic acid **3**, do not significantly affect absorption with maxima between 313 and 315 nm (Figure 2B). The emissions of ester **2** (357 nm), acid **3** (356 nm), and amide **4** (357 nm) show nearly identical maxima (Figure 2B). The additional emission bands of acid **3** (394, 417, and 441 nm) were identified as H-bonded dimers and higher-order aggregates; this assignment was confirmed by adding MeOH to the measured solution and by measuring the fluorescence spectrum in the solid state (see the SI for details).

On the other hand, the substitution of phosphine oxide **5** for its sulfur **6** and selenium **7** analogues has a more pronounced effect on both absorption and emission spectra (Figure 2C). The absorption maximum increases from oxide **5** (319 nm) to sulfide **6** (330 nm) and selenide **7** (344 nm) (Figure 2C). The emission maximum progresses from sulfide **6** (357 nm) to selenide **7** (376 nm) and further to oxide **5** (394 nm), representing the largest red-shift among the λ^5^-derivatives (Figure 2C). Finally, λ^4^-phosphonium **8** possesses absorption maxima at 276, 292, 346, and 361 nm and displays an emission maximum at 445 nm (Figure 2C).

As demonstrated, some phosphorus derivatives, including phosphinic acid **3**, ester **2**, and amide **4**, showed only a slight impact on optical behavior, which can be suitable when nonoptical characteristics of the phosphaaromate are targeted for modification or when one of the derivatives is more readily available synthetically. Conversely, compounds **5**–**7** undergo a significant change in optical properties when oxygen is replaced with sulfur or selenium, serving as a representation of the fine-tuning of the properties in the final structure.

The absolute photoluminescence quantum yields (φ_PL_) of the synthesized organophosphorus derivatives were found to be modest, ranging from 0.9% to 3.8%. Detailed photophysical data for all compounds are provided in Table S1.

### Redox Properties

To investigate the electrochemical behavior of these new compounds, cyclic voltammetry (CV) and differential pulse voltammetry (DPV) measurements were performed, and the results are summarized in Table 1 and Figure 3. The potential values of the redox processes were assessed from DPV data. The resulting electrochemical data provided valuable insight into how structural modifications within the phenanthrene framework influence the redox behavior of these compounds, particularly relative to the first reduction potential, *E*_red1_.

**Figure 3:**
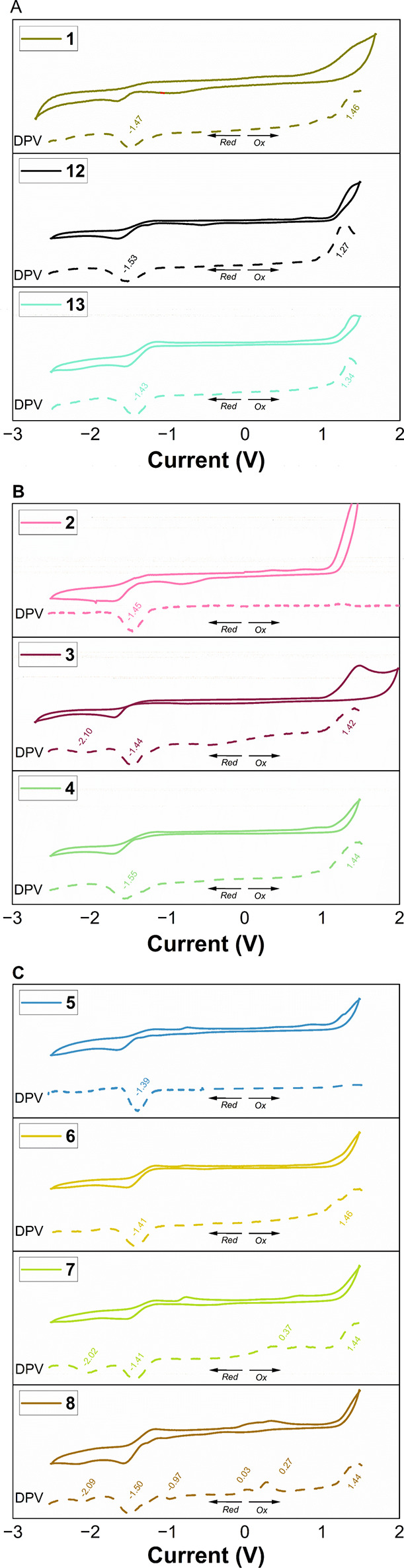
Cyclic voltammograms and differential cyclic voltammograms (scan rate of 100 mV s^–1^) in DCM with 0.1 M nBu_4_N­(ClO_4_) vs Fc/Fc^+^ of compounds (A) **1**, **12**, and **13**, (B) **2**–**4**, and (C) **5**–**8**. The data are presented according to IUPAC convention. The scan was initiated at 0.0 V and proceeded first in the anodic direction to 2.5 mV for **3**, 2.2 mV for **1**, **6**, **7**, and **13**, 2.0 mV for **4** and **5**, 1.6 mV for **1** and **12**, and 1.2 mV for **2**. The scan was then reversed toward the cathodic limit of −1.5 mV for **5**, −2.0 mV for **1** and **5**, −2.2 mV for **3**, **4**, **7**, **8**, **12**, and **13**, and −2.5 mV for **2** before returning to the initial potential.

Regarding the employed heteroatom in the phenanthrene scaffold, the values of *E*_red1_ are comparable: −1.53, −1.47, and −1.43 V for compounds **12**, **1** (P-doped analogue), and **13** (N-doped analogue), respectively.

Minor *E*_red1_ shifts were observed based on the phosphorus functionality. Phosphinate **2** and acid **3** derivatives exhibit *E*_red1_ values of −1.45 and −1.44 V, respectively. In contrast, the amide **4** derivative, featuring a weak electron-donating group, exhibits a lower potential of −1.55 V. Notably, acid derivative **3** shows an additional reduction wave at −2.10 V.^46^

Oxide **5**, sulfur **6**, and selenide **7** analogues exhibit similar reduction potentials, with oxide **5** at −1.39 V and both **6** and **7** at −1.41 V. Selenide **7** also displays an additional wave at −2.02 V. Phosphonium **8** shows a significantly shifted *E*_red1_ at −0.97 V, attributed to the methylation of the P center;^47^ this derivative further exhibits two additional reduction waves at −1.50 and −2.09 V.

Generally, derivatives with stronger electron-withdrawing groups (oxide, acid, and ester) on the aromatic system exhibit higher potentials than those with electron-donating groups.

In addition, most compounds also exhibit irreversible oxidation waves above 0 V, a phenomenon also observed in similar conjugated systems containing phosphorus.^32,36,47^

### Calculations

For comparison of phosphinine **1** and its derivatives containing a carbon atom (**12**) or a nitrogen atom (**13**) in place of phosphorus, HOMO/LUMO energies and energy gaps (*E*_g_) were calculated (using DFT calculations at the B3LYP/6-311G+ + (d,p) level of theory (Figure 4A)). The shapes of the frontier MOs are similar in all cases, but their relative energies are shifted in both the HOMO and the LUMO. The HOMO of nitrogen derivative **13** is decreased by more than 0.35 eV in comparison to that of parent phenanthrene **12**, while in phosphinine **1**, it is stabilized by 0.09 eV. More significant is the stabilization of the LUMO, which shows decreases of 0.37 eV upon introduction of nitrogen and up to 0.58 eV in the phosphorus species, which underlines the ability of phosphinines to act as π-acceptors.^48^ The changes in energy levels of the MOs together contribute to the decrease in *E*_g_ by almost 0.5 eV for **1**, while the *E*_g_ for **13** was maintained at virtually the same level as that of parent phenanthrene **12**. A more detailed analysis of the HOMOs for phosphinine **1** (Figure S16) shows that the HOMO contributes to π-donation, while the phosphorus lone pair (as a weak σ-donor) in **1** occupies the more diffuse, partly delocalized, and less directional HOMO–2 and HOMO–5 (in red), in comparison with the nitrogen lone pair as a strong δ-donor in **13** on HOMO–2 and HOMO–4 (in red).^49^

**Figure 4:**
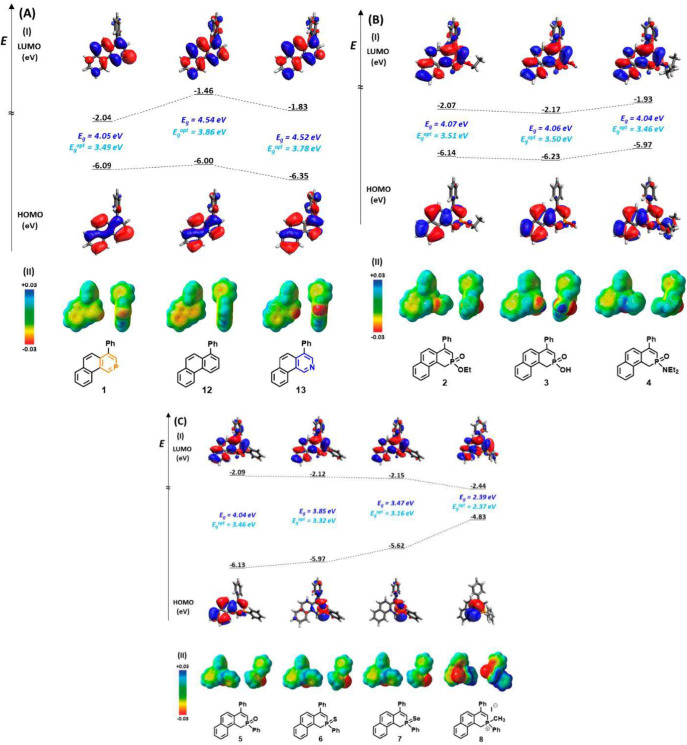
(I) Calculated HOMO/LUMO orbitals and energy gaps (*E*_g_) (DFT, B3LYP/6-311G+ + (d,p)) and experimental values of optical HOMO/LUMO energy gaps (in blue, *E*_g_^opt^) for compounds (A) **1**, **12**, and **13**, (B) **2**–**4**, and (C) **5**–**8**. (II) Electrostatic potentials mapped onto the electron density isosurface for series compounds in the range from −0.03 (red) to 0.03 (blue).

In the case of phosphinate **2**, phosphinic acid **3**, and phosphinamide **4**, the energy values of HOMO/LUMO levels shift with substitution, though the energy gap (*E*_g_) remains essentially very similar (Figure 4B). However, in the case of phosphine oxide **5**, sulfide **6**, selenide **7**, and phosphonium iodide **8** (Figure 4C), the *E*_g_ values are decreasing dramatically (from 4.04 eV for **5** to 2.39 eV for **8**), mainly due to the increase in HOMO energy (from −6.13 eV for **5** to −4.83 eV for **8**), supported by a large change in their shapes.

The electrostatic potential (EP) maps from the side and top views of compounds **1**, **12**, and **13**, which are shown in Figure 4A (part II), exhibit a more positive EP (orange) for **1** than **12**, in accordance with the phosphorus π-electron-accepting property (empty 3d/δ* orbitals).^50^ On the other hand, the heteroatom substitution with a lone pair induces negative charge (red) accumulation for **13**. Furthermore, the sp^2^ hybridization of the electronegative nitrogen within the aromatic moiety contributes to the strong electron-accepting character of the conjugated backbone. Similar behavior is shown in EP maps for other studied aromatic phosphorus compounds **2**–**8** (Figure 4B,C, part II).

The theoretical UV spectra of compounds **1**–**8**, **12**, and **13** were calculated using the TD-DFT method at the B3LYP/6-311G+ + (d,p) level. The vertical energy electronic transitions, corresponding wavelengths with oscillator strength (*f*), and their major orbital contributions for compounds **1**–**8**, **12**, and **13** are summarized in Tables S1–S10. The obtained values for **1**, **12**, and **13** indicate that the lowest singlet excited states S1 (HOMO → LUMO) are only moderately connected to the ground state (*f* = 0.152–0.209), and for **2**–**8** (*f* = 0.04–0.06), they are even lower, which corresponds to a weaker overlap of the HOMO/LUMO wave functions. As expected, the other higher excited states of **1**–**8** are strongly connected (*f* = 0.239–0.570).

## Conclusion

In summary, we have developed a versatile synthetic methodology for the derivatization of a phosphorus atom incorporated into an aromatic scaffold, enabling access to a much broader range of phosphorus species beyond commonly used phosphine chalcogenides. Starting from phosphinate **2**, this approach allowed the synthesis and thorough characterization of five new phenanthrene-based derivatives, including phosphinic acid derivatives (**3** and **4**), phosphine chalcogenides (**6** and **7**), and phosphonium derivative **8**.

All prepared compounds were thoroughly compared both theoretically (DFT calculations) and experimentally (optical and redox properties). As evidenced by the obtained data, phosphinic acid derivatives **2**–**4** do not exhibit significant changes in their optical or redox properties. This is particularly advantageous when the objective is to modify the physical properties (e.g., solubility or stability) of the whole molecule without affecting its optical and redox characteristics, or when a particular phosphorus derivative is more readily accessible synthetically. On the other hand, conversion of phosphine oxide **5** to its sulfur **6** and selenium **7** analogues has a more pronounced effect on the optical properties. The absorption maximum increases from oxide **5** (319 nm) to sulfide **6** (330 nm) and selenide **7** (344 nm). In contrast, the emission maximum shifts from sulfide **6** (357 nm) to selenide **7** (376 nm) and to oxide **5** (394 nm). These results indicate that the optical properties can be effectively tuned by appropriate selection of the phosphorus functional group. Furthermore, all prepared compounds exhibit irreversible oxidation waves above 0 V.

Phenanthrene derivative **12** and its nitrogen analogue, **13**, were also prepared, and the optical and electrochemical properties of both substances were compared to those of prepared 3-phosphaphenanthrene **1**. As a direct consequence of LUMO stabilization, phosphaphenanthrene **1** induces a bathochromic shift relative to those of its carbo **12** and nitrogen **13** analogues. In contrast, the first reduction potentials remain comparable across the series.

These findings establish a framework for understanding how modulation at the phosphorus center can be used to systematically adjust the optoelectronic properties. This will contribute to the appropriate selection of phosphorus groups in sophisticated systems, thereby facilitating the achievement of the desired properties in relation to their specific application.

## Experimental Section

### Materials and Methods

^1^H, ^13^C­{^1^H}, ^31^P, and ^31^P­{^1^H} NMR spectra were recorded by using a Bruker Avance 400 MHz instrument. Chemical shifts are reported in parts per million (δ) relative to TMS and PPh_3_ (−6 ppm) or referenced to residuals of CDCl_3_ (δ = 7.26 and 77.00 ppm, respectively) or DMSO (δ = 2.50 and 39.52 ppm, respectively). Coupling constants *J* are given in hertz. The HMBC experiments were set up for a *J*_C–H_ of 5 Hz. For the correct assignment of the ^1^H and ^13^C NMR spectra of the key compounds, COSY, HSQC, and HMBC experiments were performed. GC–MS analyses were performed on an Agilent 6890 gas chromatograph coupled to an Agilent 5973 mass spectrometer operating in 70 eV ionization mode. A DB-5MS column (30 m × 0.25 mm × 0.25 μm) was used with He as a carrier gas at a flow rate of 1.0 mL/min. The initial temperature was held at 50 °C for 3 min, programmed at a rate of 10 °C/min to 290 or 310 °C. The injection port was set at 250, 300, or 310 °C, depending on the volatility of the sample, and the *m*/*z* values are given along with their relative intensities (percent). For exact mass measurement, the spectra were internally calibrated using Na formate or tuning mix APCI-TOF. ESI and APCI high-resolution mass spectra were measured in positive mode by a micrOTOF QIII mass spectrometer (Bruker) and were determined by Compass Data Analysis software. TLC was performed on silica gel 60 F254- or 60 RP-18 F254 S-coated aluminum sheets, and compounds were visualized by UV light (254 and 366 nm). Column chromatography was performed on an HPFC Biotage Isolera One system with prepacked flash silica gel columns KP-Sil Silica and KP-C18-HS cartridges (0.040–0.063 mm). Absorption and fluorescence spectra were performed in a quartz cuvette with a 1 cm optical path using a JASCO (Tokyo, Japan) FP-8300 spectrofluorometer controlled by the Spectra Manager II software and equipped with a JASCO PMU-830 low-temperature accessory. To calibrate the instrument, calibrated light sources (Jasco ESC-842, ESC-843) were utilized. Data were acquired using the following measurement conditions: excitation and emission bandwidth of 5 nm, scanning speed set at 100 nm/min, and a data interval of 0.5 nm. Absolute photoluminescence quantum yields (φ_PL_) were determined by using an integrating sphere accessory. Samples were prepared in spectroscopic grade dichloromethane (DCM) and measured in a quartz cuvette. The excitation wavelength was set to 240 nm. All samples were measured within a concentration range of 1 × 10^–6^ M in dichloromethane. Cyclic voltammetry experiments were carried out with a LabVIEW2011 (CV and DPV) electrochemical analyzer using a Pt wire as the control electrode, glassy carbon as the working electrode, and Ag/AgCl as the reference electrode. The surface area of the working glassy carbon disk electrode was 0.071 cm^2^ (*d* = 3 mm). The surface was mechanically polished to a mirror-like finish using an aqueous alumina slurry on a microcloth pad (MicroCloth Polishinf Cloth, Buehler). The measurements were carried out in a degassed 1 mM dichloromethane solution containing 0.1 M tetra-*N*-butyl ammonium perchlorate as a supporting electrolyte with scan rates of 100 mV s^–1^ at room temperature. Ferrocene was used as an internal reference at the end of the experiments. The data are presented according to IUPAC convention. The scan was initiated at 0.0 V and proceeded first in the anodic direction to 2.5 mV for **3**, 2.2 mV for **1**, **6**, **7**, and **13**, 2.0 mV for **4** and **5**, 1.6 mV for **1** and **12**, and 1.2 mV for **2**. The scan was then reversed toward the cathodic limit of −1.5 for **5**, −2.0 mV for **1** and **5**, −2.2 mV for **3**, **4**, **7**, **8**, **12**, and **13**, and −2.5 for **2** before returning to the initial potential. The standard Schlenk technique was used for all reactions. Solvents were degassed by five freeze–pump–thaw cycles. Toluene was freshly distilled from sodium/benzophenone under an argon atmosphere. Dichloromethane was freshly distilled from calcium hydride under an argon atmosphere. Other solvents were of HPLC quality. Commercially available reagents were purchased from Merck, Fluorochem, Abcr, Apollo Scientific, or TCI Europe and used as received. Unless otherwise noted, all reactions requiring increased temperatures were performed using a Heidolph MR Hei-Tec magnetic stirring hot plate equipped with an integrated Pt1000 temperature sensor. To ensure uniform heat distribution and minimize the risk of localized hot spots associated with traditional oil baths, the reaction vessels (typically recovery or round-bottom flasks) were seated in contoured aluminum heating blocks. Photochemical reactions were performed in a custom-engineered irradiation vessel fabricated from high-purity quartz to ensure maximum ultraviolet transmittance (λ > 200 nm). The radiation source consisted of a high-pressure mercury lamp (Osram HQL 400 W), which was directly immersed in the reaction medium via a double-walled quartz cooling well. To maintain a constant internal temperature of 25 °C and prevent thermal degradation, the immersion well was continuously cooled with recirculating water. The in-house-fabricated apparatus utilized the polychromatic emission profile characteristic of Hg discharge, providing high-intensity spectral lines at 254, 313, 365, 405, and 436 nm.

### 1-(Bromomethyl)­naphthalene

2-Naphthalenemethanol (5000 mg, 31.61 mmol) was dissolved in toluene (80 mL) under an argon atmosphere. Subsequently, phosphorus tribromide (1.49 mL, 15.80 mmol, 0.5 equiv) was added to the solution. The reaction mixture was stirred at 110 °C for 90 min. After the reaction mixture was cooled to room temperature, the mixture was stirred overnight. The reaction was quenched with water, and the aqueous layer was extracted with toluene. The combined organic phases were dried over anhydrous magnesium sulfate, which was later filtered, and concentrated under reduced pressure to afford 1-(bromomethyl)­naphthalene as a yellow liquid (6990 mg, 31.61 mmol, quantitative yield): ^1^H NMR (400 MHz, CDCl_3_) δ 8.09 (dd, *J* = 8.6, 1.1 Hz, 1H), 7.82 (dt, *J* = 8.1, 0.9 Hz, 1H), 7.77 (dd, *J* = 8.3, 1.1 Hz, 1H), 7.55 (ddd, *J* = 8.4, 6.9, 1.4 Hz, 1H), 7.47 (ddt, *J* = 8.2, 6.9, 3.5 Hz, 2H), 7.34 (dd, *J* = 8.3, 7.0 Hz, 1H), 4.90 (s, 2H). This corresponds to the literature.^51^

### Ethyl (naphthalen-1-ylmethyl)­(phenylethynyl)­phosphinate

1-(Bromomethyl)­naphthalene (6000 mg, 27.14 mmol) was dissolved in diethyl­(phenylethynyl)­phosphite (7700 mg, 29.85 mmol,1.1 equiv) and heated at 100 °C for 2 h. The reaction mixture was purified by flash chromatography (1:1 petroleum ether/ethyl acetate). Phosphinate (9000 mg, 26.92 mmol, quantitative yield) was obtained as a green-brown liquid: ^1^H NMR (500 MHz, CDCl_3_) δ 8.21–8.14 (m, 1H), 7.86 (dd, *J* = 7.7, 1.8 Hz, 1H), 7.80 (dd, *J* = 8.2, 3.2 Hz, 1H), 7.58–7.42 (m, 4H), 7.41–7.35 (m, 1H), 7.29 (ddd, *J* = 8.3, 7.0, 0.8 Hz, 2H), 7.24–7.21 (m, 2H), 4.17–4.05 (m, 2H), 3.86 (d, *J*_P–H_ = 20.7 Hz, 2H), 1.29 (t, *J* = 7.1 Hz, 3H); ^31^P­{^1^H} NMR (202 MHz, CDCl_3_) δ 18.54. This corresponds to the literature.^11^

### 2-Ethoxy-4-phenyl-1*H*-benzo­[*h*]­isophosphinoline 2-Oxide (**2**)

Ethyl (naphthalen-1-ylmethyl)­(phenylethynyl)­phosphinate (7560 mg, 22.61 mmol) was dissolved in 70 mL of trifluoroacetic acid and heated at 60 °C overnight. After cooling to 0 °C, the solution was basified with aqueous NaOH. The mixture was extracted with dichloromethane, and the combined organic layers were washed with brine, dried over anhydrous magnesium sulfate, and filtered. The solvent was removed under reduced pressure, and the residue was purified by flash chromatography (ethyl acetate) to yield phosphinate **2** as a yellowish white solid (3840 g, 11.50 mmol, 51% yield): mp 135.9–140.0 °C; ^1^H NMR (400 MHz, CDCl_3_) δ 8.13 (d, *J* = 8.6 Hz, 1H), 7.84–7.81 (m, 1H), 7.68–7.59 (m, 2H), 7.54 (ddd, *J* = 8.0, 6.8, 1.1 Hz, 1H), 7.46–7.41 (m, 3H), 7.35 (ddt, *J* = 5.1, 3.6, 2.1 Hz, 2H), 7.18 (d, *J* = 8.7 Hz, 1H), 6.34 (d, *J*_P–H_ = 10.2 Hz, *J*_H–H_ = 0.9 Hz, 1H), 4.20–4.08 (m, 2H), 3.86–3.59 (m, 3H), 1.28 (t, *J* = 7.0 Hz, 3H); ^31^P­{^1^H} NMR (162 MHz, CDCl_3_) δ 33.69. This corresponds to the literature.^11^

### 2,4-Diphenyl-1*H*-benzo­[*h*]­isophosphinoline 2-Oxide (**5**)

Phosphinate **2** (200 mg, 0.60 mmol) and phosphorus pentachloride (187 mg, 0.89 mmol, 1.5 equiv) were dissolved in freshly distilled dichloromethane (5 mL) under an argon atmosphere and heated to reflux for 3 h. The solvent was removed under reduced pressure, and the crude residue with chloride **9** was dissolved in freshly distilled tetrahydrofuran (5 mL) and cooled to 0 °C. Phenyl magnesium bromide (1.0 M in THF, 0.87 mL, 0.87 mmol, 1.5 equiv) was added dropwise, and the mixture was stirred for 2 h. The reaction was quenched with saturated aqueous NH_4_Cl, and the sample extracted with ethyl acetate. The combined organic layers were dried over anhydrous magnesium sulfate. After the filtration of the drying agent, the residue was purified by flash chromatography (ethyl acetate) followed by reverse-phase flash chromatography (methanol). Phosphine oxide **5** was obtained as a yellowish-white solid (160 mg, 0.43 mmol, 73% yield): mp 142.8–146.2 °C; ^1^H NMR (400 MHz, CDCl_3_) δ 8.12 (d, *J* = 8.4 Hz, 1H), 7.90–7.80 (m, 3H), 7.69 (d, *J* = 8.8 Hz, 1H), 7.60–7.51 (m, 5H), 7.49–7.41 (m, 5H), 7.28 (s, 1H), 6.51 (d, *J* = 14.0 Hz, 1H), 4.21 (dd, *J*_P–H_ = 20.7 Hz, *J*_H–H_ = 17.2 Hz, 1H), 3.71 (dd, *J*_P–H_ = 17.2 Hz, *J*_H–H_ = 11.6 Hz, 1H); ^31^P­{^1^H} NMR (162 MHz, CDCl_3_) δ 16.74. This corresponds to the literature.^52^

### 2-(Diethylamino)-4-phenyl-1*H*-benzo­[*h*]­isophosphinoline 2-Oxide (**4**)

Phosphinate **2** (50 mg, 0.15 mmol) and phosphorus pentachloride (37 mg, 0.18 mmol, 1.2 equiv) were dissolved in freshly distilled dichloromethane (2 mL) under an argon atmosphere and refluxed for 3 h. The solvent was removed under reduced pressure, and the resulting solid of phosphinyl chloride **9** was dissolved in freshly distilled dichloromethane (2 mL). Diethylamine (0.39 mL, 0.37 mmol, 2.5 equiv) was added, and the reaction mixture was stirred overnight. The crude mixture was purified by gradient flash chromatography (petroleum ether/ethyl acetate, 50–100%) followed by reverse-phase flash chromatography (MeOH and 2% CHCl_3_) to afford phosphinamide **4** as a white amorphous solid (27 mg, 0.07 mmol, 50% yield): ^1^H NMR (400 MHz, CDCl_3_) δ 8.14 (d, *J* = 8.6 Hz, 1H), 7.81 (dd, *J* = 8.2, 1.4 Hz, 1H), 7.63 (d, *J* = 8.5 Hz, 1H), 7.61–7.57 (m, 1H), 7.52 (ddd, *J* = 7.9, 6.8, 1.1 Hz, 1H), 7.46–7.40 (m, 3H), 7.38–7.31 (m, 2H), 7.17 (d, *J* = 8.7 Hz, 1H), 6.24 (d, *J* = 9.3 Hz, 1H), 3.72 (d, *J* = 18.7 Hz, 2H), 3.06 (m, 4H), 1.01 (t, *J* = 7.1 Hz, 6H); ^13^C­{^1^H} NMR (101 MHz, CDCl_3_) δ 158.1 (d, *J*_P–C_ = 4.0 Hz), 141.8 (d, *J*_P–C_ = 16.1 Hz), 133.8, 132.2 (d, *J*_P–C_ = 9.2 Hz), 130.5 (d, *J*_P–C_ = 15.5 Hz), 123.0 (d, *J*_P–C_ = 6.5 Hz), 128.9, 128.7, 128.4, 127.2, 126.9, 126.9, 127.0, 126.9, 124.1, 120.7 (d, *J*_P–C_ = 115.5 Hz), 38.7 (d, *J*_P–C_ = 4.4 Hz), 28.4 (d, *J*_P–C_ = 88.5 Hz), 14.8 (d, *J*_P–C_ = 2.2 Hz); ^31^P­{^1^H} NMR (162 MHz, CDCl_3_) δ 25.92; HRMS (ESI/QTOF) *m*/*z* [M + H]^+^ calcd for [C_23_H_25_NOP]^+^ 362.1668, found 362.1667 (100%).

### 2-Hydroxy-4-phenyl-1*H*-benzo­[*h*]­isophosphinoline 2-Oxide (**3**)

Phosphinate **2** (200 mg, 0.60 mmol) and phosphorus pentachloride (150 mg, 0.72 mmol, 1.2 equiv) were dissolved in freshly distilled dichloromethane (5 mL) under an argon atmosphere and refluxed for 3 h. The solvent was removed under reduced pressure, and the resulting solid of phosphinyl chloride **9** was dissolved in chloroform and allowed to stand undisturbed overnight. The resulting white precipitate formed was collected by filtration to afford phosphinic acid **3** as a white solid (48 mg, 0.16 mmol, 26% yield): mp 256.2–256.6 °C; ^1^H NMR (400 MHz, DMSO-*d*_6_) δ 11.21 (s, 1H), 8.35 (d, *J* = 8.5 Hz, 1H), 7.92 (d, *J* = 8.0 Hz, 1H), 7.77 (d, *J* = 8.7 Hz, 1H), 7.61 (dt, *J* = 23.7, 7.3 Hz, 2H), 7.48 (d, *J* = 6.1 Hz, 3H), 7.39–7.33 (m, 2H), 7.07 (d, *J* = 8.7 Hz, 1H), 6.33 (d, *J* = 9.9 Hz, 1H), 3.66 (d, *J* = 19.5 Hz, 2H); ^13^C­{^1^H} NMR (101 MHz, DMSO-*d*_6_) δ 155.4 (d, *J*_P–C_ = 4.6 Hz), 141.2 (d, *J*_P–C_ = 16.1 Hz), 133.0, 132.0 (d, *J*_P–C_ = 9.3 Hz), 130.4 (d, *J*_P–C_ = 6.7 Hz), 130.2 (d, *J*_P–C_ = 17.1 Hz), 128.6, 128.6, 128.4, 128.1, 127.0, 126.8, 126.4, 126.1 (d, *J*_P–C_ = 2.2 Hz), 124.8, 122.4 (d, *J*_P–C_ = 122.3 Hz), 28.1 (d, *J*_P–C_ = 96.9 Hz); ^31^P­{^1^H} NMR (162 MHz, DMSO-*d*_6_) δ 25.39; HRMS (ESI/QTOF) *m*/*z* [M + H]^+^ calcd for [C_19_H_16_O_2_P]^+^ 307.0882, found 307.0886 (100%).

### 2,4-Diphenyl-1*H*-benzo­[*h*]­isophosphinoline 2-Sulfide (**6**)

Phosphine oxide **5** (105 mg, 0.29 mmol) was dissolved in freshly distilled dichloromethane (2 mL) under an argon atmosphere and cooled to 0 °C. Trichlorosilane (0.14 mL, 1.38 mmol, 4.8 equiv) was added, and the reaction mixture was stirred at 0 °C for 2 h, followed by an additional 2 h at rt. After the solvent was removed under reduced pressure, the crude material was analyzed by ^31^P NMR, which displayed a peak at −42 ppm and confirmed the presence of phosphine intermediate **10**. Without further purification, the crude solid was combined with sulfur S_8_ (18.3 mg, 0.07 mmol, 0.25 equiv) and dissolved in freshly distilled toluene (2 mL) under an argon atmosphere. The reaction mixture was stirred at rt overnight, after which the solvent was removed under reduced pressure. Purification by gradient flash chromatography (petroleum ether/ethyl acetate, 10–50%) afforded desired phosphine sulfide **6** as a yellowish-white amorphous solid (52 mg, 0.14 mmol, 48% yield): mp 196.0–196.9 °C; ^1^H NMR (400 MHz, CDCl_3_) δ 7.90 (d, *J* = 8.4 Hz, 1H), 7.83–7.75 (m, 2H), 7.64 (dd, *J* = 7.9, 1.5 Hz, 1H), 7.51 (d, *J* = 8.7 Hz, 1H), 7.41–7.27 (m, 3H), 7.29–7.20 (m, 6H), 7.09–7.06 (m, 2H), 6.24 (d, *J* = 17.5 Hz, 1H), 4.00–3.89 (m, 2H); ^13^C­{^1^H} NMR (101 MHz, CDCl_3_) δ 155.6, 141.1 (d, *J*_P–C_ = 14.7 Hz), 134.2, 132.3 (d, *J*_P–C_ = 14.7 Hz), 132.1, 132.1 (d, *J*_P–C_ = 3.0 Hz), 131.5, 131.3 (d, *J*_P–C_ = 10.7 Hz), 130.6 (d, *J*_P–C_ = 15.6 Hz), 129.0, 128.9, 128.8, 128.7 (d, *J*_P–C_ = 1.4 Hz), 127.6, 127.6 (d, *J*_P–C_ = 1.7 Hz), 127.4, 127.1, 126.8 (d, *J*_P–C_ = 2.1 Hz), 124.2, 119.2 (d, *J*_P–C_ = 80.2 Hz), 34.1 (d, *J*_P–C_ = 58.0 Hz); ^31^P­{^1^H} NMR (162 MHz, CDCl_3_) δ 21.02; HRMS (ESI/QTOF) *m*/*z* [M + H]^+^ calcd for [C_25_H_20_PS]^+^ 383.1018, found 383.1024 (100%).

### 2,4-Diphenyl-1*H*-benzo­[*h*]­isophosphinoline 2-Selenide (**7**)

Phosphine oxide **5** (100 mg, 0.27 mmol) was dissolved in freshly distilled dichloromethane (2 mL) under an argon atmosphere and cooled to 0 °C. Trichlorosilane (0.13 mL, 1.31 mmol, 4.8 equiv) was added, and the reaction mixture was stirred at 0 °C for 2 h, followed by an additional 2 h at rt. The solvent was removed under reduced pressure, and resulting crude phosphine intermediate **10** was dissolved in freshly distilled tetrahydrofuran (2 mL) together with selenium (65 mg, 0.82 mmol, 3 equiv). The mixture was stirred at room temperature overnight, filtered through Celite, and washed with ethyl acetate. The filtrate was concentrated under reduced pressure, and the residue was purified by flash chromatography (1:1 petroleum ether/ethyl acetate) to afford phosphine selenide **7** as a white amorphous solid with a characteristic odor (23 mg, 0.05 mmol, 20% yield): mp 190.0–191.2 °C; ^1^H NMR (400 MHz, CDCl_3_) δ 7.89 (d, *J* = 8.5 Hz, 1H), 7.86–7.76 (m, 2H), 7.64 (d, *J* = 8.0 Hz, 1H), 7.51 (d, *J* = 8.7 Hz, 1H), 7.36 (dt, *J* = 21.3, 7.4 Hz, 2H), 7.24 (d, *J* = 15.6 Hz, 7H), 7.10–7.04 (m, 2H), 6.31 (d, *J* = 18.9 Hz, 1H), 4.30–4.00 (m, 2H); ^13^C­{^1^H} (101 MHz, CDCl_3_) δ 155.1, 140.9 (d, *J*_P–C_ = 14.5 Hz), 134.2, 132.1 (d, *J*_P–C_ = 3.1 Hz), 132.0 (d, *J*_P–C_ = 7.8 Hz), 131.8 (d, *J*_P–C_ = 11.0 Hz), 130.7 (d, *J*_P–C_ = 29.2 Hz), 130.3 (d, *J*_P–C_ = 29.7 Hz), 129.1, 129.0, 128.9, 128.8, 128.7 (d, *J*_P–C_ = 1.7 Hz), 127.6, 127.4, 127.1, 126.8, 126.8, 124.2, 118.1 (d, *J*_P–C_ = 72.4 Hz), 34.1 (d, *J*_P–C_ = 51.5 Hz); ^31^P­{^1^H} NMR (162 MHz, CDCl_3_) δ 16.55; HRMS (ESI/QTOF) *m*/*z* [M + H]^+^ calcd for [C_25_H_20_PSe]^+^ 431.0464, found 431.0459 (100%).

### 2-Methyl-2,4-diphenyl-1,2-dihydrobenzo­[*h*]­isophosphinolin-2-ium Iodide (**8**)

Phosphine oxide **5** (100 mg, 0.27 mmol) was dissolved in freshly distilled dichloromethane (2 mL) under an argon atmosphere and cooled to 0 °C. Trichlorosilane (0.13 mL, 1.31 mmol, 4.8 equiv) was added, and the mixture was stirred at 0 °C for 2 h, followed by an additional 2 h at rt. After the solvent was removed under reduced pressure, the crude residue with phosphine **10** was dissolved in freshly distilled tetrahydrofuran (2 mL) and cooled to 0 °C. Methyl iodide (0.03 mL, 0.43 mmol, 1.5 equiv) was added dropwise, and the reaction mixture was stirred overnight. The reaction mixture was filtered through Celite, and the solvent was removed under reduced pressure. The resulting solid was dissolved in a minimal amount of dichloromethane and layered with heptane. The brown precipitate that formed was collected by filtration, washed with heptane, and further purified by reverse-phase chromatography (acetonitrile) to afford phosphonium salt **8** as a brown solid (50 mg, 0.14 mmol, 48% yield): mp 146.6–148.8 °C; ^1^H NMR (400 MHz, CDCl_3_) δ 8.49 (d, *J* = 8.7 Hz, 1H), 8.08–7.98 (m, 2H), 7.84 (d, *J* = 8.1 Hz, 1H), 7.80–7.49 (m, 11H), 7.27–7.25 (m, 1H), 6.57–6.42 (m, 1H), 4.86 (dt, *J* = 137.2, 16.9 Hz, 2H), 2.77–2.69 (m, 3H); ^13^C­{^1^H} NMR (101 MHz, CDCl_3_) δ 165.4, 139.5 (d, *J*_P–C_ = 15.1 Hz), 135.2 (d, *J*_P–C_ = 3.2 Hz), 134.7, 132.3 (d, *J*_P–C_ = 11.1 Hz), 131.8 (d, *J*_P–C_ = 8.5 Hz), 130.7, 130.5 (d, *J*_P–C_ = 12.9 Hz), 130.1 (d, *J*_P–C_ = 16.4 Hz), 129.0 (d, *J*_P–C_ = 22.7 Hz), 129.0 (d, *J*_P–C_ = 1.8 Hz), 128.7 (d, *J*_P–C_ = 25.7 Hz), 126.8 (d, *J*_P–C_ = 2.2 Hz), 125.4, 124.5 (d, *J*_P–C_ = 8.9 Hz), 118.2 (d, *J*_P–C_ = 86.3 Hz), 101.9 (d, *J*_P–C_ = 84.5 Hz), 23.2 (d, *J*_P–C_ = 59.2 Hz), 9.4 (d, *J*_P–C_ = 57.4 Hz); ^31^P­{^1^H} NMR (162 MHz, CDCl_3_) δ 4.36; HRMS (ESI/QTOF) *m*/*z* [M]^+^ calcd for [C_26_H_22_P]^+^ 365.1454, found 365.1459 (100%).

### 4-Phenylbenzo­[*h*]­isophosphinoline (**1**)

Phosphinate **2** (500 mg, 1.50 mmol) and phosphorus pentachloride (374 mg, 1.79 mmol, 1.2 equiv) were dissolved in freshly distilled dichloromethane (10 mL) under an argon atmosphere. The reaction mixture was then heated to reflux for 3 h. The solvent was then removed under reduced pressure. In a separate flask, trichlorosilane (0.45 mL, 4.49 mmol, 3 equiv) was dissolved in freshly distilled toluene (5 mL) and cooled to 0 °C. Pyridine (1.1 mL, 13.46 mmol, 9 equiv) was added dropwise, and the mixture was stirred at 0 °C for 10 min. The residue from the first part of the reaction (containing the phosphine chloride) was dissolved in freshly distilled toluene (5 mL) and added to the cooled solution. The combined mixture was heated at 90 °C and stirred overnight. The reaction mixture was purified by filtration through a short pad of alumina, and solvent removal under reduced pressure afforded phosphinine **1** as a yellow amorphous solid (400 mg, 1.47 mmol, 98% yield): ^1^H NMR (400 MHz, CDCl_3_) δ 10.31 (dd, *J* = 32.5, 2.7 Hz, 1H), 8.87 (d, *J* = 8.4 Hz, 1H), 8.55 (dd, *J* = 37.9, 2.7 Hz, 1H), 7.89 (dd, *J* = 7.8, 1.5 Hz, 1H), 7.81–7.65 (m, 4H), 7.56–7.44 (m, 5H), 7.21–7.14 (m, 1H); ^31^P­{^1^H} NMR (162 MHz, CDCl_3_) δ 187.04. This corresponds to the literature.^11^

Other references 11,45,51–60 can be found in the Supporting Information.

## Supplementary Material





## Data Availability

The data underlying this study are available in the published article and its Supporting Information.

## Notes

FAIR data, including the primary NMR FID files, for compounds **1–8**, **12**, **13**, **S1–S5**, 1-(bromomethyl)­naphthalene, ethyl (naphthalen-1-ylmethyl), (phenylethynyl)­phosphinate, and diphenyliodonium trifluoromethanesulfonate (ZIP)
^1^H, ^13^C, and ^31^P NMR spectra and computational data (PDF)
