# Supplementary figures and images for: Transformation of the Phosphorus Atom in Hexacyclic Polyaromates and Its Impact on Physicochemical Properties
Source: J Org Chem. 2026 May 13;91(20):6884–94. doi: 10.1021/acs.joc.6c00184 (PMC13200189; doi:10.1021/acs.joc.6c00184)

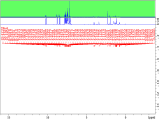

Supplement: Supplementary file 1 [file jo6c00184_si_001.zip › phenanthrene_FID/1/1_H/pdata/1/thumb.png]

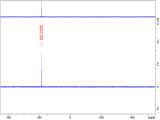

Supplement: Supplementary file 1 [file jo6c00184_si_001.zip › phenanthrene_FID/1/31_P/pdata/1/thumb.png]

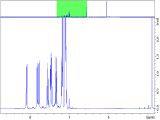

Supplement: Supplementary file 1 [file jo6c00184_si_001.zip › phenanthrene_FID/1-(bromomethyl)naphthalene/1_H/pdata/1/thumb.png]

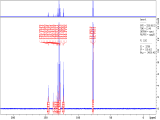

Supplement: Supplementary file 1 [file jo6c00184_si_001.zip › phenanthrene_FID/13/13_C/pdata/1/thumb.png]

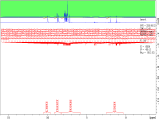

Supplement: Supplementary file 1 [file jo6c00184_si_001.zip › phenanthrene_FID/13/1_H/pdata/1/thumb.png]

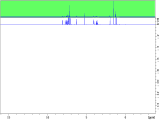

Supplement: Supplementary file 1 [file jo6c00184_si_001.zip › phenanthrene_FID/2/1_H/pdata/1/thumb.png]

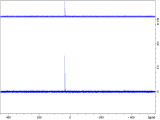

Supplement: Supplementary file 1 [file jo6c00184_si_001.zip › phenanthrene_FID/2/31_P/pdata/1/thumb.png]
