# Supplementary material for: Transformation of the Phosphorus Atom in Hexacyclic Polyaromates and Its Impact on Physicochemical Properties
Source: J Org Chem. 2026 May 13;91(20):6884–94. doi: 10.1021/acs.joc.6c00184 (PMC13200189; doi:10.1021/acs.joc.6c00184)
Supplement: Supplementary file 2 [file jo6c00184_si_002.pdf]

Transformation of Phosphorus Atom in Hexacyclic Polyaromates and its Impact on Physico-Chemical Properties

Eliška Mizerová,[a,b] Joaquín Almarza González,[a] Vladimír Církva,[a] Jaroslav Žádný[a], Jan Storch,[a] Martin Jakubec[a], Tomáš Beránek[a,*]

[a] Institute of Chemical Process Fundamentals of the Czech Academy of Sciences, Rozvojová 1/135, 165 00 Prague 6, Czech Republic

[b] Department of Organic Chemistry, University of Chemistry and Technology Prague, Technická 5, 166 28 Prague 6, Czech Republic

Table of contents

1. Materials and Methods 2
2. Synthetic Procedures 4
   2.1 Synthesis of 1-phenylphenanthrene (**12**) 10
   2.2 Synthesis of 4-phenylbenzo[*h*]isoquinoline (**13**) 12
3. ^{1}H, ^{13}C and ^{31}P NMR Spectra of prepared Compounds 15
4. DFT Calculations 31
5. Quantum yields 52
6. Comparison of solid-state and solution-phase emission for acid **3** 52
7. References 53

1. Materials and Methods

^{1}H, $^{13}C\{^{1}H\}$, ^{31}P, and $^{31}P\{^{1}H\}$ NMR spectra were recorded using a Bruker Avance 400 MHz instrument. Chemical shifts are reported in parts per million (δ) relative to TMS and PPh_3 (−6 ppm) or referenced to residuals of $CDCl_3$ (δ = 7.26 and δ = 77.00 ppm, respectively) or DMSO (δ = 2.50 and 39.52 ppm, respectively). The coupling constants *J* are given in hertz. The HMBC experiments were set up for J_{C-H} = 5 Hz. For the correct assignment of both the ^{1}H and ^{13}C NMR spectra of key compounds, COSY, HSQC, and HMBC experiments were performed. GC−MS analyses were performed on an Agilent 6890 gas chromatograph coupled to an Agilent 5973 mass spectrometer operating in 70 eV ionization mode. A DB-5MS column (30 m × 0.25 mm × 0.25 μm) was used with He as a carrier gas at a 1.0 mL/min flow rate. The initial temperature was 50 °C held for 3 min, programmed at 10°C/min to 290 °C or 310 °C. The injection port was set at 250, 300, or 310 °C, depending on the volatility of the sample, and the m/z values are given along with their relative intensities (%). For exact mass measurement, the spectra were internally calibrated using Na formate or tuning mix APCI-TOF. ESI and APCI high-resolution mass spectra were measured in positive mode by a micrOTOF QIII mass spectrometer (Bruker) and were determined by Compass Data Analysis software. TLC was performed on silica gel 60 F254- or 60 RP- 18 F254 S-coated aluminum sheets and compounds were visualized by UV light (254 and 366 nm). Column chromatography was performed on an HPFC Biotage Isolera One system with prepacked flash silica gel columns KP-Sil Silica and KP-C18-HS cartridges (0.040−0.063 mm). Absorption and fluorescence spectra were performed in a quartz cuvette with a 1 cm optical path using a JASCO FP-8300 spectrofluorometer (Tokyo, Japan) controlled by the Spectra Manager II software and equipped with a JASCO PMU-830 low-temperature accessory. To calibrate the instrument, calibrated light sources (Jasco ESC-842, ESC-843) were utilized. Data acquisition was performed using the following measurement conditions: excitation and emission bandwidth of 5 nm, scanning speed set at 100 nm/min, and a data interval of 0.5 nm. Absolute photoluminescence quantum yields (ϕ_{PL}) were determined using an integrating sphere accessory. Samples were prepared in spectroscopic grade dichloromethane (DCM) and measured in a quartz cuvette. The excitation wavelength was set to 240 nm. All samples were measured within a concentration range of 10^{-6} M in dichloromethane. Cyclic voltammetry experiments were carried out with LabVIEW2011 (CV and DPV) electrochemical analyser using Pt wire control electrode, glassy carbon as working electrode and Ag/AgCl as a reference electrode. The surface area of the working glassy carbon disk electrode is 0.071 cm^2 (d = 3 mm). The surface was mechanically polished to a mirror-like finish using an aqueous alumina slurry on a microcloth pad (MicroCloth Polishinf Cloth, Buehler). The measurements were carried out in the degassed 1 mM dichloromethane solution containing 0.1 M tetra-*N*-butyl ammonium perchlorate as a supporting electrolyte with scan rates of 100 mVs^{-1} at room temperature.

Ferrocene was used as an internal reference at the end of the experiments. The data is presented according to IUPAC convention. The scan initiated at 0.0 V and proceeded first in the anodic direction to 2.5 mV for 3; 2.2 mV for 1, 6, 7, and 13; 2.0 mV for 4 and 5; 1.6 mV for 1, and 12; 1.2 mV for 2. The scan was then reversed towards the cathodic limit of -1.5 for 5; -2.0 mV for 1 and 5; -2.2 mV for 3, 4, 7, 8, 12 and 13; -2.5 for 2 before returning to the initial potential. The standard Schlenk technique was used for all reactions. Solvents were degassed by five freeze-pump-thaw cycles. Toluene was freshly distilled from sodium/benzophenone under an atmosphere of argon. Dichloromethane was freshly distilled from calcium hydride under an atmosphere of argon. Other solvents were used in HPLC quality. Commercially available reagents were purchased from Merck, Fluorochem, Abcr, Apollo Scientific or TCI Europe and used as received. Unless otherwise noted, all reactions requiring elevated temperatures were performed using a Heidolph MR Hei-Tec magnetic stirring hotplate equipped with an integrated Pt1000 temperature sensor. To ensure uniform heat distribution and minimize the risk of localized hotspots associated with traditional oil baths, the reaction vessels (typically recovery or round-bottom flasks) were seated in contoured aluminum heating blocks. Photochemical reactions were performed in a custom-engineered irradiation vessel fabricated from high-purity quartz to ensure maximum ultraviolet transmittance ($\lambda > 200$ nm). The radiation source consisted of a high-pressure mercury lamp (Osram HQL 400 W), which was directly immersed into the reaction medium via a double-walled quartz cooling well. To maintain a constant internal temperature of 25 °C and prevent thermal degradation, the immersion well was continuously cooled with recirculating water. The in-house fabricated apparatus utilized the polychromatic emission profile characteristic of the Hg-discharge, providing high-intensity spectral lines at 254 nm, 313 nm, 365 nm, 405 nm, and 436nm.

Safety statement

The chemicals used in this study, including trifluoroacetic acid (TFA), meta-chloroperoxybenzoic acid (*m*CPBA), iodobenzene, phosphorus tribromide (PBr_3), phosphorus pentachloride (PCl_5), triethyl phosphite, and sodium hydride (NaH), are hazardous and should be handled with extreme care. Phosphorus tribromide and phosphorus pentachloride are highly corrosive and react violently with moisture to release toxic hydrogen halide gases. Sodium hydride is a flammable, pyrophoric solid that reacts exothermically with water to release hydrogen gas. Meta-chloroperoxybenzoic acid is a strong oxidant and potentially explosive. Trifluoroacetic acid is a highly corrosive and volatile strong acid. Triethyl phosphite is a malodorous and should be used strictly within a fume hood. All procedures involving these reagents were performed in a well-ventilated fume hood using appropriate personal protective equipment including gloves, a lab coat, and eye protection. No unexpected or unusually hazardous conditions were encountered in this work.

2. Synthetic Procedures

1-(Bromomethyl)naphthalene

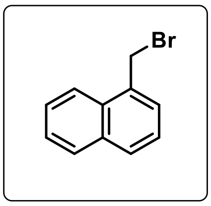

2-Naphthalenemethanol (5000 mg, 31.61 mmol) was dissolved in toluene (80 mL) under an argon atmosphere. Subsequently, phosphorus tribromide (1.49 ml, 15.80 mmol, 0.5 eq) was added to the solution. The reaction mixture was stirred at 110 °C for 90 minutes. After cooling the reaction to room temperature, the mixture was stirred overnight. The reaction was quenched with water, and the aqueous layer was extracted with toluene. The combined organic phases were dried over anhydrous magnesium sulphate, which was later filtered, and concentrated under reduced pressure to afford 1-(bromomethyl)naphthalene as a yellow liquid (6990 mg, 31.61 mmol, quantitative yield).

^{1}H NMR (400 MHz, $CDCl_3$) δ 8.09 (dd, J = 8.6, 1.1 Hz, 1H), 7.82 (dt, J = 8.1, 0.9 Hz, 1H), 7.77 (dd, J = 8.3, 1.1 Hz, 1H), 7.55 (ddd, J = 8.4, 6.9, 1.4 Hz, 1H), 7.47 (ddt, J = 8.2, 6.9, 3.5 Hz, 2H), 7.34 (dd, J = 8.3, 7.0 Hz, 1H), 4.90 (s, 2H) ppm.

Corresponds to the literature.[1]

Ethyl (naphthalen-1-ylmethyl)(phenylethynyl)phosphinate

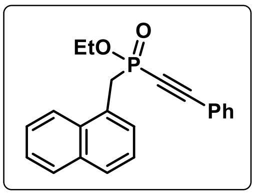

1-(Bromomethyl)naphthalene (6000 mg, 27.14 mmol) was dissolved in diethyl(phenylethynyl)phosphite (7700 mg, 29.85 mmol,1.1 eq) and heated at 100 °C for two hours. The reaction mixture was purified by flash chromatography (petroleum ether/ethyl acetate, 1:1). Phosphinate (9000 mg, 26.92 mmol, quantitative yield) was obtained as a green-brown liquid.

^{1}H NMR (500 MHz, $CDCl_3$) δ 8.21 – 8.14 (m, 1H), 7.86 (dd, J = 7.7, 1.8 Hz, 1H), 7.80 (dd, J = 8.2, 3.2 Hz, 1H), 7.58 – 7.42 (m, 4H), 7.41 – 7.35 (m, 1H), 7.29 (ddd, J = 8.3, 7.0, 0.8 Hz, 2H), 7.24 – 7.21 (m, 2H), 4.17 – 4.05 (m, 2H), 3.86 (d, $J_{P\text{-}H}$ = 20.7 Hz, 2H), 1.29 (t, J = 7.1 Hz, 3H) ppm.

^{31}P{^{1}H} NMR (202 MHz, $CDCl_3$) δ 18.54 ppm.

Corresponds to the literature.[2]

*2-Ethoxy-4-phenyl-1H-benzo[h]isophosphinoline 2-oxide (**2**)*

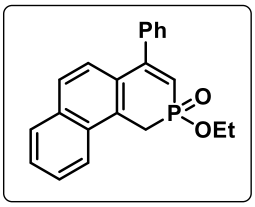

Ethyl (naphthalen-1-ylmethyl)(phenylethynyl)phosphinate (7560 mg, 22.61 mmol) was dissolved in 70 mL of trifluoroacetic acid and heated at 60 °C overnight. After cooling to 0 °C, the solution was basified with aqueous NaOH. The mixture was extracted with dichloromethane and the combined organic layers were washed with brine, dried over anhydrous magnesium sulphate and filtered. The solvent was removed under reduced pressure and the residue was purified by flash chromatography (ethyl acetate) to yield the phosphinate **2** as a yellowish white solid (3840 g, 11.50 mmol, 51% yield). mp = 135.9 – 140.0 °C

^{1}H NMR (400 MHz, $CDCl_3$) δ 8.13 (d, *J* = 8.6 Hz, 1H), 7.84 – 7.81 (m, 1H), 7.68 – 7.59 (m, 2H), 7.54 (ddd, *J* = 8.0, 6.8, 1.1 Hz, 1H), 7.46 – 7.41 (m, 3H), 7.35 (ddt, *J* = 5.1, 3.6, 2.1 Hz, 2H), 7.18 (d, *J* = 8.7 Hz, 1H), 6.34 (d, J_{P-H} = 10.2, J_{H-H} = 0.9 Hz, 1H), 4.20 – 4.08 (m, 2H), 3.86 – 3.59 (m, 3H), 1.28 (t, *J* = 7.0 Hz, 3H) ppm.

^{31}P{^{1}H} NMR (162 MHz, $CDCl_3$) δ 33.69 ppm.

Corresponds to the literature.[2]

*2,4-Diphenyl-1H-benzo[h]isophosphinoline 2-oxide (**5**)*

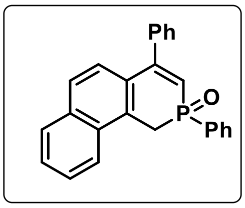

Phosphinate **2** (200 mg, 0.60 mmol) and phosphorus pentachloride (187 mg, 0.89 mmol, 1.5 eq) were dissolved in freshly distilled dichloromethane (5 mL) under an argon atmosphere and heated to reflux for 3 hours. The solvent was removed under reduced pressure, and the crude residue with chloride **9** was dissolved in freshly distilled tetrahydrofuran (5 mL) and cooled to 0 °C. Phenyl magnesium bromide (1.0 M in THF, 0.87 mL, 0.87 mmol, 1.5 eq) was added dropwise, and the mixture and stirred for 2 hours. The reaction was quenched with saturated aqueous NH_4Cl and extracted with ethyl acetate. The combined organic layers were dried over anhydrous magnesium sulphate. After the filtration of the drying agent, the residue was purified by flash chromatography (ethyl acetate) followed by reverse-phase flash chromatography (methanol). The phosphine oxide **5** was obtained as a yellowish-white solid (160 mg, 0.43 mmol, 73% yield). mp = 142.8 – 146.2 °C

^{1}H NMR (400 MHz, $CDCl_3$) δ 8.12 (d, *J* = 8.4 Hz, 1H), 7.90 – 7.80 (m, 3H), 7.69 (d, *J* = 8.8 Hz, 1H), 7.60 – 7.51 (m, 5H), 7.49 – 7.41 (m, 5H), 7.28 (s, 1H), 6.51 (d, *J* = 14.0 Hz, 1H), 4.21 (dd, J_{P-H} = 20.7, J_{H-H} =17.2 Hz, 1H), 3.71 (dd, J_{P-H} = 17.2, J_{H-H} =11.6 Hz, 1H) ppm.

^{31}P{^{1}H} NMR (162 MHz, $CDCl_3$) δ 16.74 ppm.

Corresponds to the literature.[3]

*2-(Diethylamino)-4-phenyl-1H-benzo[h]isophosphinoline 2-oxide (**4**)*

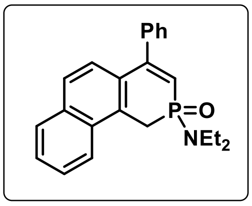

Phosphinate **2** (50 mg, 0.15 mmol) and phosphorus pentachloride (37 mg, 0.18 mmol, 1.2 eq) were dissolved in freshly distilled dichloromethane (2 mL) under an argon atmosphere and refluxed for 3 hours. The solvent was removed under reduced pressure and the resulting solid of phosphinyl chloride **9** was dissolved in freshly distilled dichloromethane (2 mL). Diethylamine (0.39 mL, 0.37 mmol, 2.5 eq) was added, and the reaction mixture was stirred overnight. The crude mixture was purified by gradient flash chromatography (petroleum ether/ethyl acetate, 50 – 100%) followed by reverse-phase flash chromatography (MeOH + 2% $CHCl_3$) to afford the phosphinamide **4** as a white amorphous solid (27 mg, 0.07 mmol, 50% yield).

^{1}H NMR (400 MHz, $CDCl_3$) δ 8.14 (d, *J* = 8.6 Hz, 1H), 7.81 (dd, *J* = 8.2, 1.4 Hz, 1H), 7.63 (d, *J* = 8.5 Hz, 1H), 7.61 – 7.57 (m, 1H), 7.52 (ddd, *J* = 7.9, 6.8, 1.1 Hz, 1H), 7.46 – 7.40 (m, 3H), 7.38 – 7.31 (m, 2H), 7.17 (d, *J* = 8.7 Hz, 1H), 6.24 (d, *J* = 9.3 Hz, 1H), 3.72 (d, *J* = 18.7 Hz, 2H), 3.06 (m, 4H), 1.01 (t, *J* = 7.1 Hz, 6H) ppm.

^{13}C{^{1}H} NMR (101 MHz, $CDCl_3$) δ 158.1 (d, J_{P-C} = 4.0 Hz), 141.8 (d, J_{P-C} = 16.1 Hz), 133.8, 132.2 (d, J_{P-C} = 9.2 Hz), 130.5 (d, J_{P-C} = 15.5 Hz), 123.0 (d, J_{P-C} = 6.5 Hz), 128.9, 128.7, 128.4, 127.2, 126.9, 126.9, 127.0, 126.9, 124.1, 120.7 (d, J_{P-C} = 115.5 Hz), 38.7 (d, J_{P-C} = 4.4 Hz), 28.4 (d, J_{P-C} = 88.5 Hz), 14.8 (d, J_{P-C} = 2.2 Hz) ppm.

^{31}P{^{1}H} NMR (162 MHz, $CDCl_3$) δ 25.92 ppm.

HRMS (ESI/QTOF) m/z $[M+H]^+$ calculated for $[C_{23}H_{25}NOP]^+$ 362.1668, found 362.1667 (100%).

*2-Hydroxy-4-phenyl-1H-benzo[h]isophosphinoline 2-oxide (**3**)*

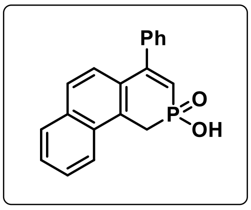

Phosphinate **2** (200 mg, 0.60 mmol) and phosphorus pentachloride (150 mg, 0.72 mmol, 1.2 eq) were dissolved in freshly distilled dichloromethane (5 mL) under argon atmosphere and refluxed for 3 hours. The solvent was removed under reduced pressure and the resulting solid of phosphinyl chloride **9** was dissolved in chloroform and allowed to stand undisturbed overnight. The resulting white precipitate formed was collected by filtration to afford phosphinic acid **3** as a white solid of (48 mg, 0.16 mmol, 26% yield). mp = 256.2 – 256.6 °C

^{1}H NMR (400 MHz, DMSO-d6) δ 11.21 (s, 1H), 8.35 (d, *J* = 8.5 Hz, 1H), 7.92 (d, *J* = 8.0 Hz, 1H), 7.77 (d, *J* = 8.7 Hz, 1H), 7.61 (dt, *J* = 23.7, 7.3 Hz, 2H), 7.48 (d, *J* = 6.1 Hz, 3H), 7.39 – 7.33 (m, 2H), 7.07 (d, *J* = 8.7 Hz, 1H), 6.33 (d, *J* = 9.9 Hz, 1H), 3.66 (d, *J* = 19.5 Hz, 2H) ppm.

$^{13}C\{^1H\}$ NMR (101 MHz, DMSO-d6) δ 155.4 (d, J_{P-C} = 4.6 Hz), 141.2 (d, J_{P-C} = 16.1 Hz), 133.0, 132.0 (d, J_{P-C} = 9.3 Hz), 130.4 (d, J_{P-C} = 6.7 Hz), 130.2 (d, J_{P-C} = 17.1 Hz), 128.6, 128.6, 128.4, 128.1, 127.0, 126.8, 126.4, 126.1 (d, J_{P-C} = 2.2 Hz), 124.8, 122.4 (d, J_{P-C} = 122.3 Hz), 28.1 (d, J_{P-C} = 96.9 Hz) ppm.

$^{31}P\{^1H\}$ NMR (162 MHz, DMSO-d6) δ 25.39 ppm.

HRMS (ESI/QTOF) m/z $[M+H]^+$ calculated for $[C_{19}H_{16}O_2P]^+$ 307.0882, found 307.0886 (100%).

*2,4-Diphenyl-1H-benzo[h]isophosphinoline 2-sulfide (**6**)*

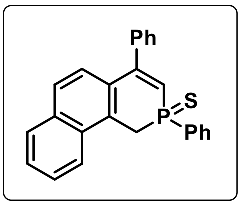

Phosphine oxide **5** (105 mg, 0.29 mmol) was dissolved in freshly distilled dichloromethane (2 mL) under an argon atmosphere and cooled to 0 °C. Trichlorosilane (0.14 mL, 1.38 mmol, 4.8 eq) was added, and the reaction mixture was stirred at 0 °C for 2 hours, followed by an additional 2 hours at rt. After the solvent removal under reduced pressure, the crude material was analysed by ^{31}P NMR, which displayed a peak at – 42 ppm and confirmed the presence of phosphine intermediate **10**. Without further purification, the crude solid was combined with sulphur S_8 (18.3 mg, 0.07 mmol, 0.25 eq) and dissolved in freshly distilled toluene (2 mL) under an argon atmosphere. The reaction mixture was stirred at rt overnight, after which the solvent was removed by reduced pressure. Purification by gradient flash chromatography (petroleum ether/ethyl acetate, 10-50%), afforded the desired phosphine sulfide **6** as a yellowish-white amorphous solid (52 mg, 0.14 mmol, 48% yield). mp = 196.0 – 196.9 °C

^1H NMR (400 MHz, $CDCl_3$) δ 7.90 (d, J = 8.4 Hz, 1H), 7.83 – 7.75 (m, 2H), 7.64 (dd, J = 7.9, 1.5 Hz, 1H), 7.51 (d, J = 8.7 Hz, 1H), 7.41 – 7.27 (m, 3H), 7.29 – 7.20 (m, 6H), 7.09 – 7.06 (m, 2H), 6.24 (d, J = 17.5 Hz, 1H), 4.00 – 3.89 (m, 2H) ppm.

$^{13}C\{^1H\}$ NMR (101 MHz, $CDCl_3$) δ 155.6, 141.1 (d, J_{P-C} = 14.7 Hz), 134.2, 132.3 (d, J_{P-C} = 14.7 Hz), 132.1, 132.1 (d, J_{P-C} = 3.0 Hz), 131.5, 131.3 (d, J_{P-C} = 10.7 Hz), 130.6 (d, J_{P-C} = 15.6 Hz), 129.0, 128.9, 128.8, 128.7 (d, J_{P-C} = 1.4 Hz), 127.6, 127.6 (d, J_{P-C} = 1.7 Hz), 127.4, 127.1, 126.8 (d, J_{P-C} = 2.1 Hz), 124.2, 119.2 (d, J_{P-C} = 80.2 Hz), 34.1 (d, J_{P-C} = 58.0 Hz) ppm.

$^{31}P\{^1H\}$ NMR (162 MHz, $CDCl_3$) δ 21.02 ppm.

HRMS (ESI/QTOF) m/z $[M+H]^+$ calculated for$[C_{25}H_{20}PS]^+$ 383.1018, found 383.1024 (100%).

*2,4-Diphenyl-1H-benzo[h]isophosphinoline 2-selenide (**7**)*

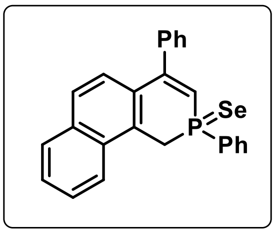

Phosphine oxide **5** (100 mg, 0.27 mmol) was dissolved in freshly distilled dichloromethane (2 mL) under an argon atmosphere and cooled to 0 °C. Trichlorosilane (0.13 mL, 1.31 mmol, 4.8 eq) was added, and the reaction mixture was stirred at 0 °C for 2 hours, followed by an additional 2 hours at rt. The solvent was removed under reduced pressure, and the resulting crude phosphine intermediate **10** was dissolved in freshly distilled tetrahydrofuran (2 mL) together with selenium (65 mg, 0.82 mmol, 3 eq). The mixture was stirred at room temperature overnight, then filtered through celite and washed with ethyl acetate. The filtrate was concentrated under reduced pressure, and the residue was purified by flash chromatography (petroleum ether/ethyl acetate, 1:1) to afford the phosphine selenide **7** as a white amorphous solid with a characteristic odor (23 mg, 0.05 mmol, 20% yield). mp = 190.0 – 191.2 °C

^{1}H NMR (400 MHz, $CDCl_3$) δ 7.89 (d, J = 8.5 Hz, 1H), 7.86 – 7.76 (m, 2H), 7.64 (d, J = 8.0 Hz, 1H), 7.51 (d, J = 8.7 Hz, 1H), 7.36 (dt, J = 21.3, 7.4 Hz, 2H), 7.24 (d, J = 15.6 Hz, 7H), 7.10 – 7.04 (m, 2H), 6.31 (d, J = 18.9 Hz, 1H), 4.30 – 4.00 (m, 2H) ppm.

^{13}C{^{1}H} (101 MHz, $CDCl_3$) δ 155.1, 140.9 (d, $J_{P\text{-}C}$ = 14.5 Hz), 134.2, 132.1 (d, $J_{P\text{-}C}$ = 3.1 Hz), 132.0 (d, $J_{P\text{-}C}$ = 7.8 Hz), 131.8 (d, $J_{P\text{-}C}$= 11.0 Hz), 130.7 (d, $J_{P\text{-}C}$ = 29.2 Hz), 130.3 (d, $J_{P\text{-}C}$ = 29.7 Hz), 129.1, 129.0, 128.9, 128.8, 128.7 (d, $J_{P\text{-}C}$ = 1.7 Hz), 127.6, 127.4, 127.1, 126.8, 126.8, 124.2, 118.1 (d, $J_{P\text{-}C}$ = 72.4 Hz), 34.1 (d, $J_{P\text{-}C}$ = 51.5 Hz) ppm.

^{31}P{^{1}H} NMR (162 MHz, $CDCl_3$) δ 16.55 ppm.

HRMS (ESI/QTOF) m/z $[M+H]^+$ calculated for$[C_{25}H_{20}PSe]^+$ 431.0464, found 431.0459 (100%).

*2-Methyl-2,4-diphenyl-1,2-dihydrobenzo[h]isophosphinolin-2-ium iodide (**8**)*

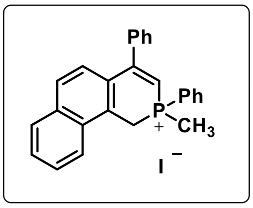

Phosphine oxide **5** (100 mg, 0.27 mmol) was dissolved in freshly distilled dichloromethane (2 mL) under an argon atmosphere and cooled to 0 °C. Trichlorosilane (0.13 mL, 1.31 mmol, 4.8 eq) was added, and the mixture was stirred at 0 °C for 2 hours, followed by an additional 2 hours at rt. After the solvent removal under reduced pressure, the crude residue with phosphine **10** was dissolved in freshly distilled tetrahydrofuran (2 mL) and cooled to 0 °C. Methyl iodide (0.03 mL, 0.43 mmol, 1.5 eq) was added dropwise, and the reaction mixture was stirred overnight. The reaction mixture was filtered through celite, and the solvent was removed under reduced pressure. The resulting solid was dissolved in a minimal amount of dichloromethane and layered with heptane. The brown precipitate formed was collected by filtration, washed with heptane,

and further purified by reverse-phase chromatography (acetonitrile) to afford the phosphonium salt **8** as a brown solid 50 mg (0.10 mmol, 37% yield). mp = 146.6 – 148.8 °C

^{1}H NMR (400 MHz, $CDCl_3$) δ 8.49 (d, *J* = 8.7 Hz, 1H), 8.08 – 7.98 (m, 2H), 7.84 (d, *J* = 8.1 Hz, 1H), 7.80 – 7.49 (m, 11H), 7.27–7.25 (m, 1H), 6.57 – 6.42 (m, 1H), 4.86 (dt, *J* = 137.2, 16.9 Hz, 2H), 2.77 – 2.69 (m, 3H) ppm.

^{31}P{^{1}H} NMR (162 MHz, $CDCl_3$) δ 4.36 ppm.

HRMS (ESI/QTOF) m/z $[M]^+$ calculated for $[C_{26}H_{22}P]^+$ 365.1454, found 365.1459 (100%).

*4-Phenylbenzo[h]isophosphinoline (**1**)*

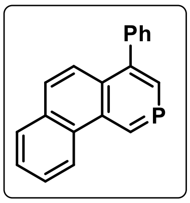

Phosphinate **2** (500 mg, 1.50 mmol) and phosphorus pentachloride (374 mg, 1.79 mmol, 1.2 eq) were dissolved in freshly distilled dichloromethane (10 mL) under an argon atmosphere. The reaction mixture was then heated to reflux for 3 hours. The solvent was then removed under reduced pressure. In a separate flask, trichlorosilane (0.45 mL, 4.49 mmol, 3 eq) was dissolved in freshly distilled toluene (5 mL) and cooled to 0 °C. Pyridine (1.1 mL, 13.46 mmol, 9 eq) was added dropwise, and the mixture was stirred at 0 °C for 10 minutes. The residue from the first part of the reaction (containing the phosphine chloride) was dissolved in freshly distilled toluene (5 mL) and added to the cooled solution. The combined mixture was heated at 90 °C and stirred overnight. The reaction mixture was purified by filtration through a short pad of alumina, and solvent removal under reduced pressure afforded the phosphinine **1** as a yellow amorphous solid (400 mg, 1.47 mmol, 98% yield).

^{1}H NMR (400 MHz, $CDCl_3$) δ 10.31 (dd, *J* = 32.5, 2.7 Hz, 1H), 8.87 (d, *J* = 8.4 Hz, 1H), 8.55 (dd, *J* = 37.9, 2.7 Hz, 1H), 7.89 (dd, *J* = 7.8, 1.5 Hz, 1H), 7.81 – 7.65 (m, 4H), 7.56 – 7.44 (m, 5H), 7.21 – 7.14 (m, 1H) ppm.

^{31}P{^{1}H} NMR (162 MHz, $CDCl_3$) δ 187.04 ppm.

Corresponds to the literature.[2]

2.1 Synthesis of 1-phenylphenanthrene (**12**)

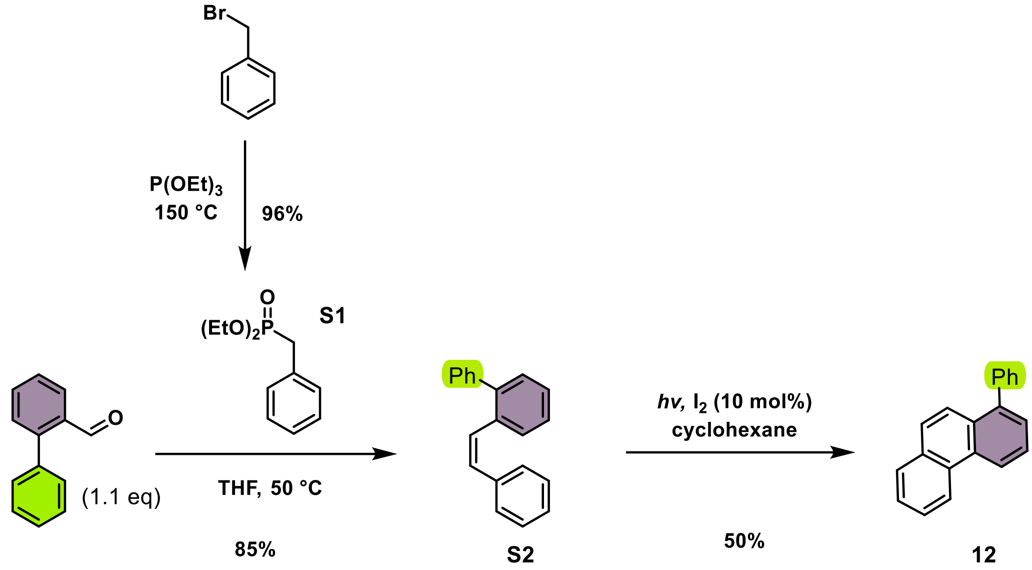

Scheme S1: Synthesis of 1-phenylphenanthrene (**12**)

*Diethyl benzylphosphonate (**S1**)*

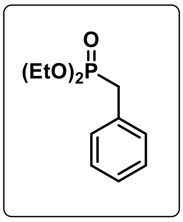

Benzyl bromide (0.49 mL, 4.09 mmol) was dissolved in triethyl phosphite (0.77 mL, 4.50 mmol, 1.1 eq) and heated at 150 °C for 3 hours. The byproducts formed were removed using high vacuum while heating at 50 °C for an hour. The product **S1** was obtained as a yellow, thick liquid (900 mg, 3.94 mmol, 96% yield).

^{1}H NMR (400 MHz, $CDCl_3$) δ 7.32 – 7.23 (m, 5H), 4.07 – 3.93 (m, 4H), 3.15 (d, *J* = 21.6 Hz, 2H), 1.24 (t, *J* = 7.0 Hz, 6H) ppm.

^{31}P{^{1}H} NMR (162 MHz, $CDCl_3$) δ 26.44 ppm.

Corresponds to the literature.[4]

*2-Styryl-1,1'-biphenyl (**S2**)*

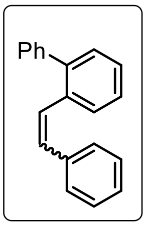

Phosphonate **S1** (560 mg, 2.45 mmol) was dissolved in freshly distilled tetrahydrofuran (11 mL) under an argon atmosphere. Sodium hydride (60% w/w, 128 mg, 3.19 mmol, 1.3 eq) was added, and the mixture was stirred for 5 minutes, before biphenyl-2-carbaldehyde (492 mg, 2.70 mmol, 1.1 eq) was introduced. The reaction mixture was heated at 50 °C overnight. After cooling to room temperature, the reaction was quenched by slow addition of water, followed by extraction with ethyl acetate. The combined organic layers were washed with water, dried over anhydrous magnesium sulphate and filtered. Removal of the solvent under reduced

pressure afforded a crude residue, which was purified by flash chromatography (petroleum ether/ethyl acetate, 9:1) to give the product **S2** as an off-white viscous liquid (533 mg, 2.08 mmol, 85% yield).

1H NMR (400 MHz, $CDCl_3$) δ 7.77 (dd, *J* = 7.4, 1.5 Hz, 1H), 7.47 – 7.34 (m, 10 H), 7.33 – 7.28 (m, 2 H), 7.25 – 7.20 (m, 1H), 7.09 (q, *J* = 16.3 Hz, 2H) ppm.

Corresponds to the literature.[5]

*1-Phenylphenanthrene (**12**)*

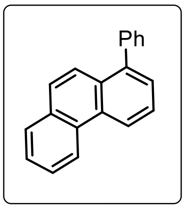

Stilbene derivative **S2** (333 mg, 1.3 mmol) and iodine (3 mg, 0.01 mmol, 10 mol%) were dissolved in cyclohexane (333 mL). The reaction mixture was irradiate d with UV light (254 nm) in a quartz photoreactor under an air atmosphere, and the reaction was monitored by GC-MS. After 2.5 hours, the reaction was quenched by washing the mixture with a saturated aqueous solution of sodium thiosulfate. The organic phase was dried over anhydrous magnesium sulphate, filtered, and concentrated under reduced pressure. The crude residue-was purified by flash chromatography (petroleum ether/ethyl acetate, 30:1) to afford the product **12** as a bright yellow viscous liquid (165 mg, 0.65 mmol, 50% yield).

^{1}H NMR (400 MHz, $CDCl_3$) δ 8.79 – 8.72 (m, 2H), 7.89 (dd, *J* = 7.8, 1.5 Hz, 1H), 7.82 (dd, *J* = 9.3, 0.8 Hz, 1H), 7.73 – 7.66 (m, 3H), 7.62 (ddd, *J* = 8.1, 7.0, 1.3 Hz, 1H), 7.56 (dd, *J* = 7.1, 1.2 Hz, 1H), 7.52 (d, *J* = 4.4 Hz, 3H), 7.45 (ddd, *J* = 8.7, 5.0, 3.8 Hz, 1H), 7.41 – 7.29 (m, 1H) ppm.

Corresponds to the literature.[6]

2.2 Synthesis of 4-phenylbenzo[*h*]isoquinoline (**13**)

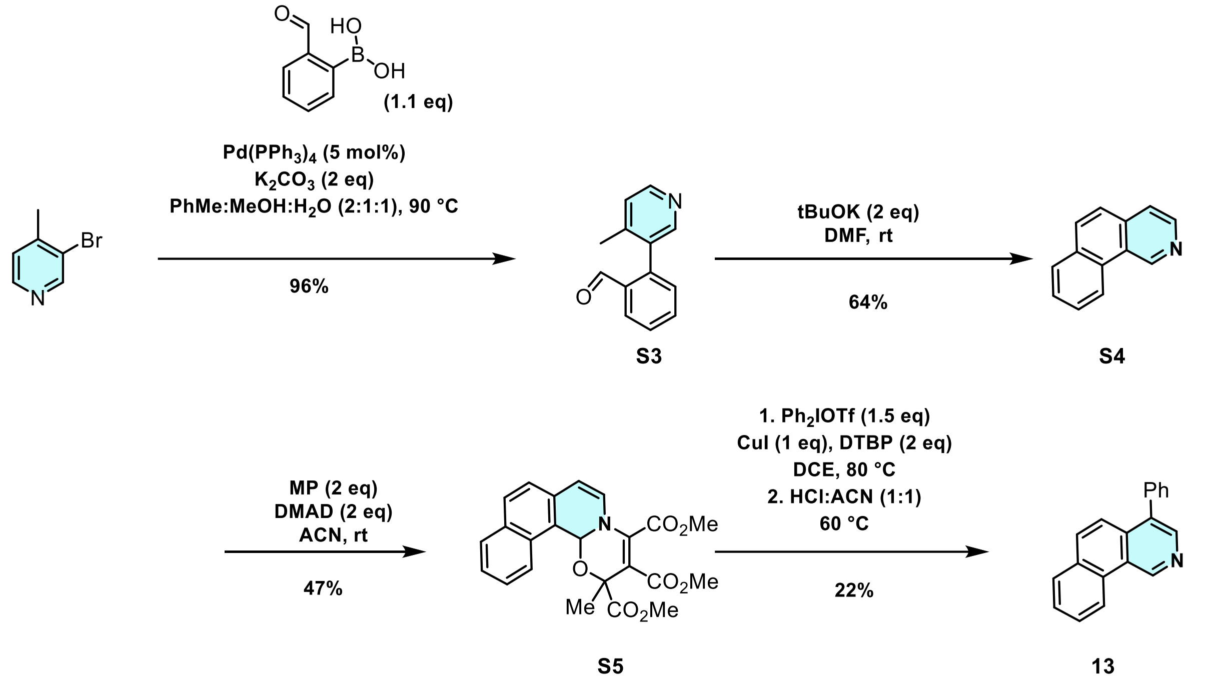

Scheme S2: Synthesis of 4-phenylbenzo[*h*]isoquinoline (**13**)

*2-(4-Methylpyridin-3-yl)benzaldehyde (**S3**)*

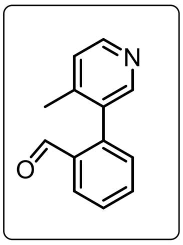

Tetrakis(triphenylphosphine)palladium (0) (168 mg, 0.15 mmol, 5 mol%) was dissolved in toluene (12 mL, dried over molecular sieves). The solution was degassed under vacuum and maintained under an argon atmosphere. 3-Bromo-4-methylpyridine (500 mg, 2.91 mmol) was then added to the solution, followed by boronic acid derivative (479 mg, 3.20 mmol, 1.1 eq) dissolved in degassed methanol (6 mL) and sodium carbonate (616 mg, 5.81 mmol, 2 eq) in degassed distilled water (6 mL). The reaction mixture was heated at 90 °C overnight. After cooling, the mixture was extracted with ethyl acetate and water, and the combined organic layers were washed with brine, dried over anhydrous magnesium sulphate, and filtered. Removal of the solvent under reduced pressure afforded a crude of **S3**, which was purified by flash chromatography (petroleum ether/ethyl acetate, 8:1 to 100% ethyl acetate). The product **S3** was obtained as a yellow, viscous liquid (548 mg, 2.78 mmol, 96% yield)

^{1}H NMR (400 MHz, $CDCl_3$) δ 9.77 (d, *J* = 0.7 Hz, 1H), 8.48 (d, *J* = 49.9 Hz, 2H), 8.05 (dd, *J* = 7.8, 1.5 Hz, 1H), 7.68 (td, *J* = 7.5, 1.5 Hz, 1H), 7.57 (tt, *J* = 7.5, 1.0 Hz, 1H), 7.29 (dd, *J* = 7.6, 1.3 Hz, 1H), 7.24 (d, *J* = 5.0 Hz, 1H), 2.12 (s, 3H) ppm.

Corresponds to the literature.[7]

*Benzo[h]isoquinoline (**S4**)*

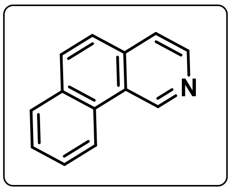

2-(4-Methylpyridin-3-yl)benzaldehyde (**S3**) (548 mg, 2.78 mmol) was dissolved in dried dimethylformamide (3 mL) under an argon atmosphere. Potassium tert-butoxide (623.5 mg, 5.56 mmol, 2 eq), pre-dissolved in dried dimethylformamide (4 mL), was added dropwise to the cooled reaction mixture at 0 °C. The mixture was then stirred at room temperature overnight. The reaction was quenched by the addition of water and extracted with dichloromethane. The combined organic layers were washed with brine and dried over anhydrous magnesium sulphate. After the filtration of the drying agent, the crude product was purified by flash chromatography (petroleum ether/ethyl acetate, 8:1) to yield **S4** as an amorphous white solid (319 mg, 1.78 mmol, 64%).

^{1}H NMR (400 MHz, $CDCl_3$) δ 10.05 (s, 1H), 8.80 (d, *J* = 8.2 Hz, 1H), 8.71 (d, *J* = 5.4 Hz, 1H), 7.93 (dd, *J* = 9.1, 2.3 Hz, 2H), 7.80 – 7.63 (m, 4H) ppm.

Corresponds to the literature.[7]

*Trimethyl 2-methyl-2H,13cH-benzo[h][1,3]oxazino[2,3-a]isoquinoline-2,3,4-tricarboxylate (**S5**)*

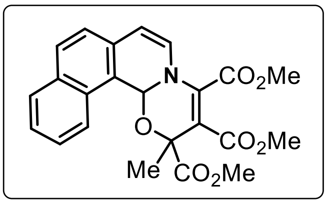

Benzo[*h*]isoquinoline (**S4**) (319 mg, 1.78 mmol) and methyl pyruvate (0.32 mL, 3.56 mmol, 2 eq) were dissolved in acetonitrile (10 mL). Dimethyl acetylenecarboxylate (0.43 mL, 3.56 mmol, 2 eq) was then added, and the reaction mixture was stirred at rt for 2 hours. The solvent was removed under reduced pressure, and the crude residue was purified by flash chromatography (petroleum ether/ethyl acetate, 4:1). The product **S5** was obtained as a yellow solid (350 mg, 0.83 mmol, 47% yield).

^{1}H NMR (400 MHz, $CDCl_3$) δ 7.85 – 7.78 (m, 2H), 7.75 – 7.71 (m, 1H), 7.50 – 7.41 (m, 2H), 7.24 (s, 1H), 6.90 (s, 1H), 6.47 (dd, *J* = 7.6, 0.6 Hz, 1H), 5.93 (d, *J* = 7.6 Hz, 1H), 3.98 (s, 3H), 3.97 (s, 3H), 3.78 (s, 3H), 2.04 (s, 3H), 1.76 (s, 3H) ppm.

Corresponds to the literature.[8]

Diphenyliodonium trifluoromethanesulfonate

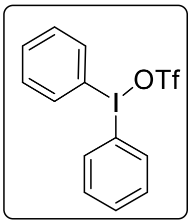

Iodobenzene (300 mg, 1.47 mmol), *m*CPBA (279 mg, 1.62 mmol, 1.1 eq) and benzene (0.14 mL, 1.62 mmol, 1.1 eq) were dissolved in dichloromethane (20 mL). Triflic acid (0.39 mL, 4.41 mmol, 3 eq) was added dropwise to the cooled solution at 0 °C, and the mixture was stirred at room temperature for 30 minutes. The solvent was removed under reduced pressure, and the residue was dissolved in diethyl ether and left to stand at – 30 °C. The resulting white precipitate was collected by filtration, washed with diethyl ether and dried to afford the desired product (427 mg, 0.99 mmol, 68% yield).

^{1}H NMR (400 MHz, $CDCl_3$) δ 8.01 – 7.94 (m, 4H), 7.68 – 7.62 (m, 2H), 7.53 – 7.46 (m, 4H) ppm.

Corresponds to the literature.[9]

*4-Phenylbenzo[h]isoquinoline (**13**)*

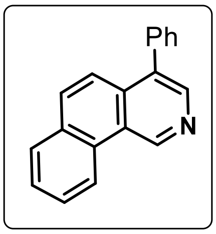

Isoquinoline derivative **S5** (280 mg, 0.66 mmol), diphenyliodonium salt (426 mg, 0.99 mmol, 1.5 eq) and copper(I) iodine (126 mg, 0.66 mmol, 1 eq) were placed in a Schlenk tube, flushed three times with argon and evacuated. Degassed dichloroethane (5 mL) and pyridine (0.29 mL, 1.32 mmol, 2 eq) were added, and the reaction mixture was stirred at 80 °C overnight. After the solvent removal under reduced pressure, the residue was dissolved in hydrochloric acid (12 M, 2 mL) and acetonitrile (2 mL) and heated at 60 °C for 5 hours. The reaction mixture was cooled to 0 °C, and a saturated aqueous solution of sodium carbonate was added dropwise. The resulting precipitate was collected by filtration and washed with acetonitrile. The filtrate was washed with brine, dried over anhydrous magnesium sulphate and filtered. The crude reaction mixture was pre-purified using flash chromatography (petroleum ether/ethyl acetate, 5:1), followed by further purification with flash chromatography (ethyl acetate) affording the product **13** as a brown-green oil (37 mg, 0.14 mmol, 22% yield).

^{1}H NMR (400 MHz, $CDCl_3$) δ 10.10 (s, 1H), 8.88 (d, J = 8.3 Hz, 1H), 8.68 (s, 1H), 8.00 – 7.89 (m, 2H), 7.83 – 7.76 (m, 2H), 7.70 (ddd, J = 8.1, 7.1, 1.2 Hz, 1H), 7.59 – 7.48 (m, 5H) ppm.

Corresponds to the literature.[8]

3. ^{1}H, ^{13}C and ^{31}P NMR Spectra of prepared Compounds

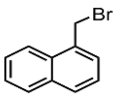

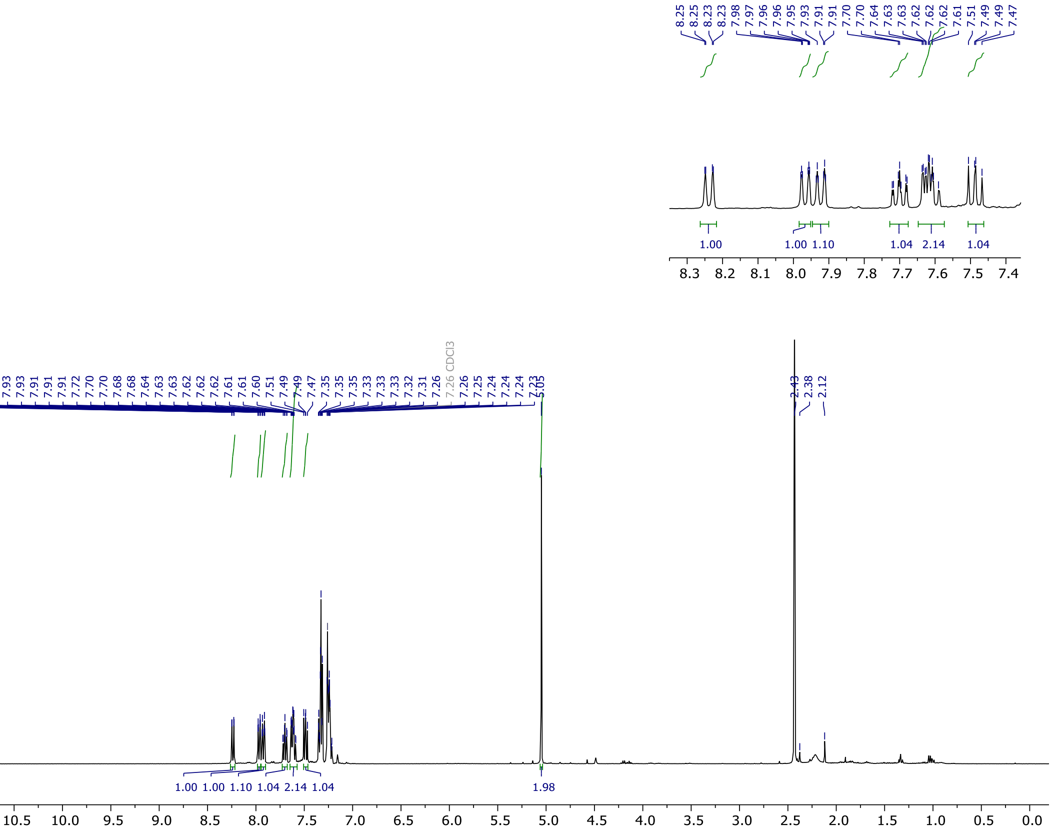

Figure S1 ^{1}H Spectrum of 1-(Bromomethyl)naphthalene (400 MHz, $CDCl_3$)

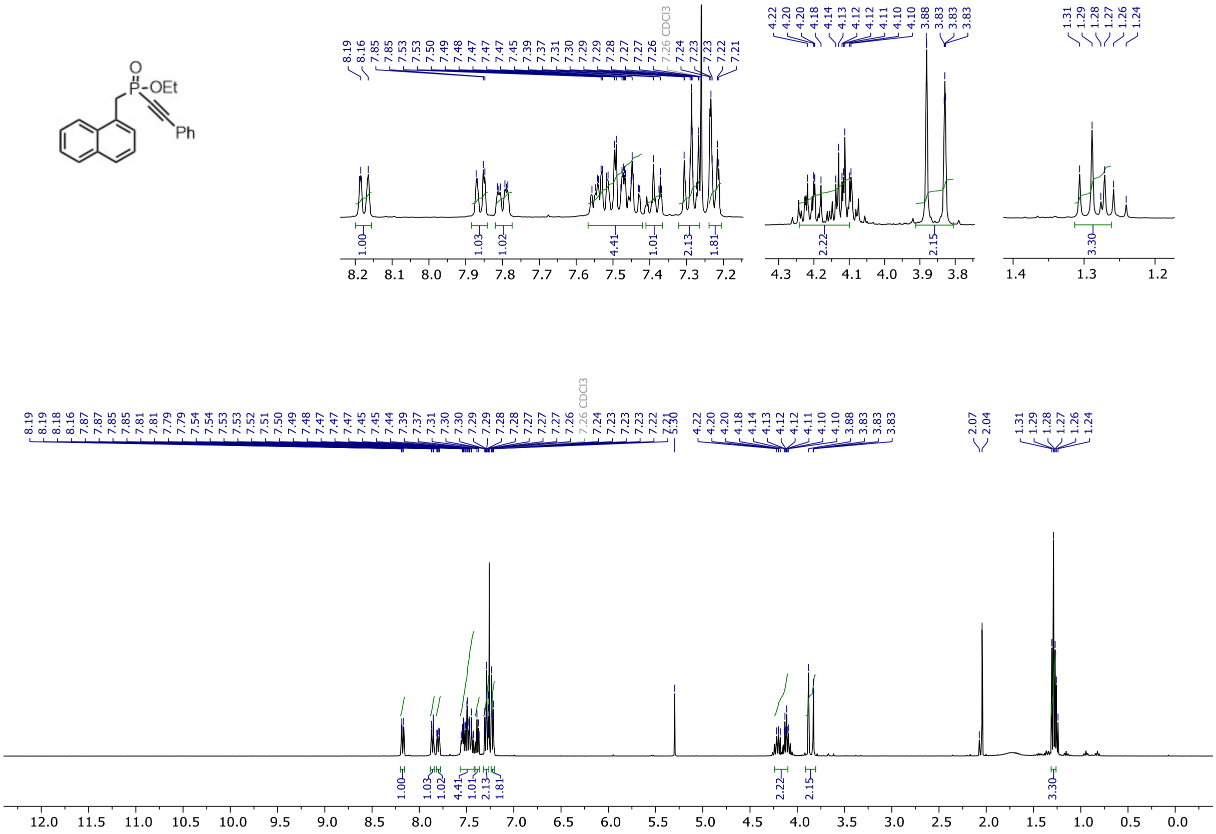

Figure S2 ^{1}H Spectrum of ethyl (naphthalen-1-ylmethyl)(phenylethynyl)phosphinate (400 MHz, $CDCl_3$)

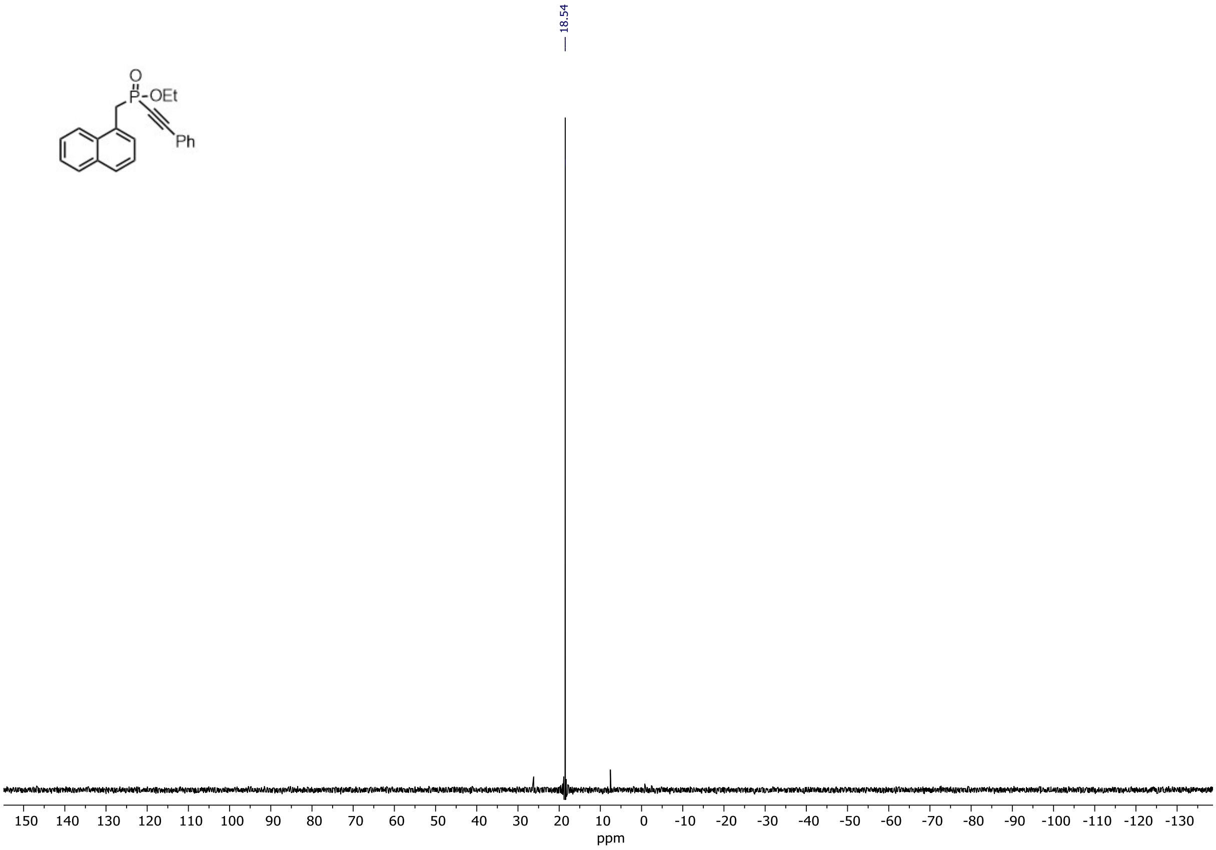

Figure S3 ^{31}P{^{1}H} Spectrum of ethyl (naphthalen-1-ylmethyl)(phenylethynyl)phosphinate (162 MHz, $CDCl_3$)

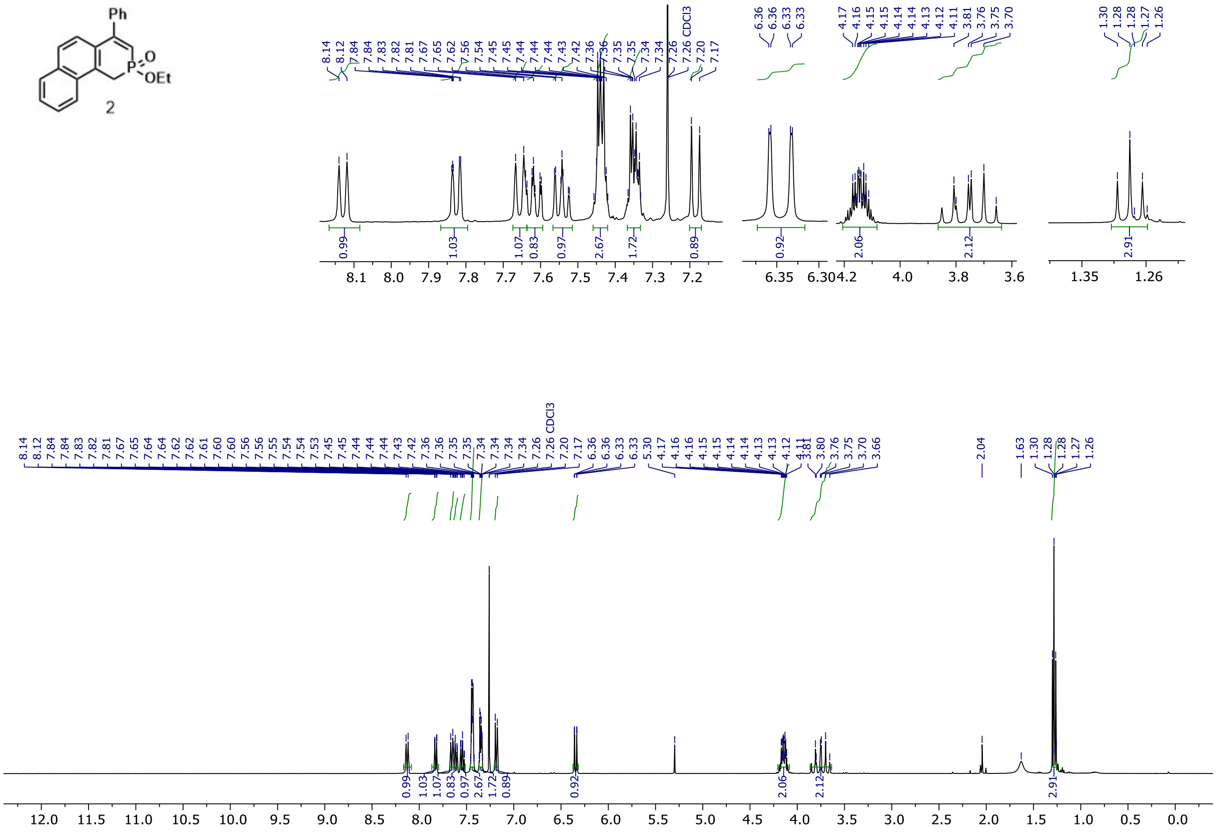

Figure S4 ^{1}H Spectrum of **2** (400 MHz, $CDCl_3$)

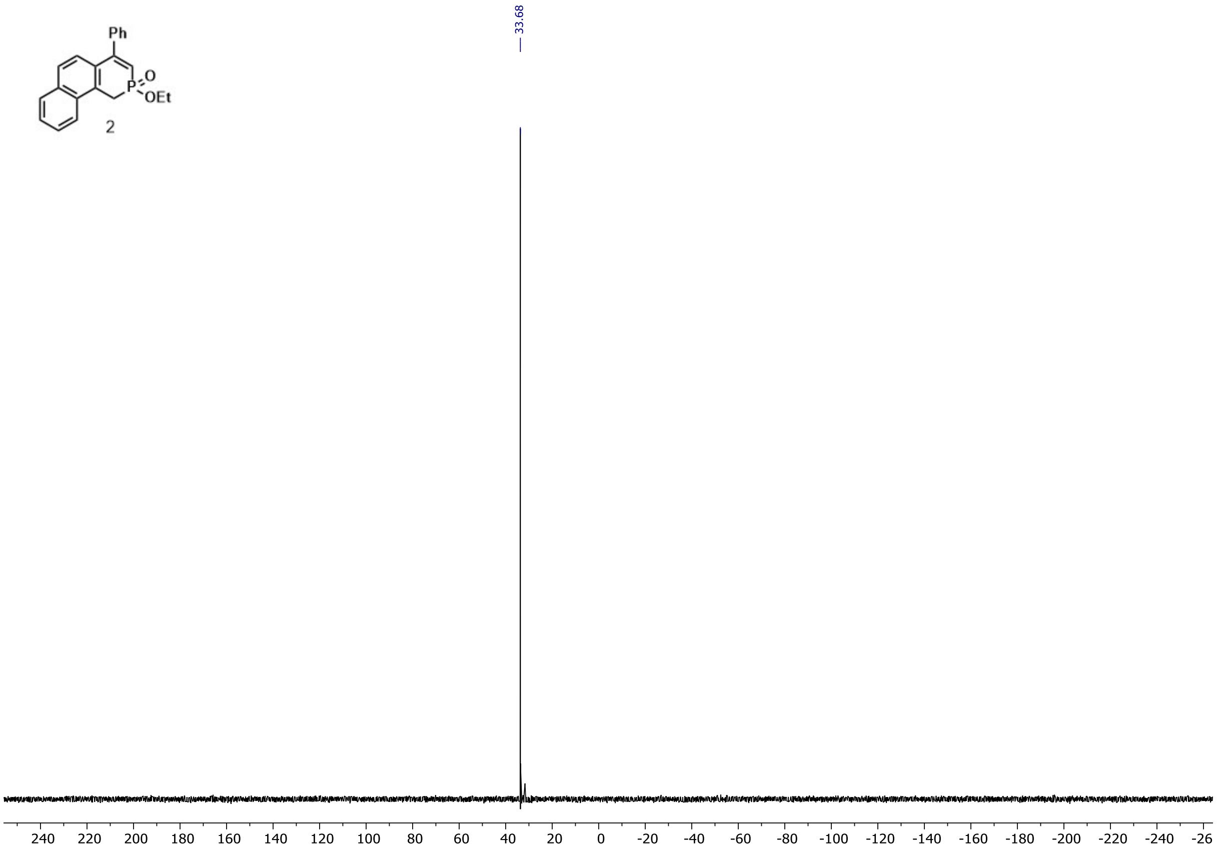

Figure S5 $^{31}P\{^{1}H\}$ Spectrum of **2** (162 MHz, $CDCl_3$)

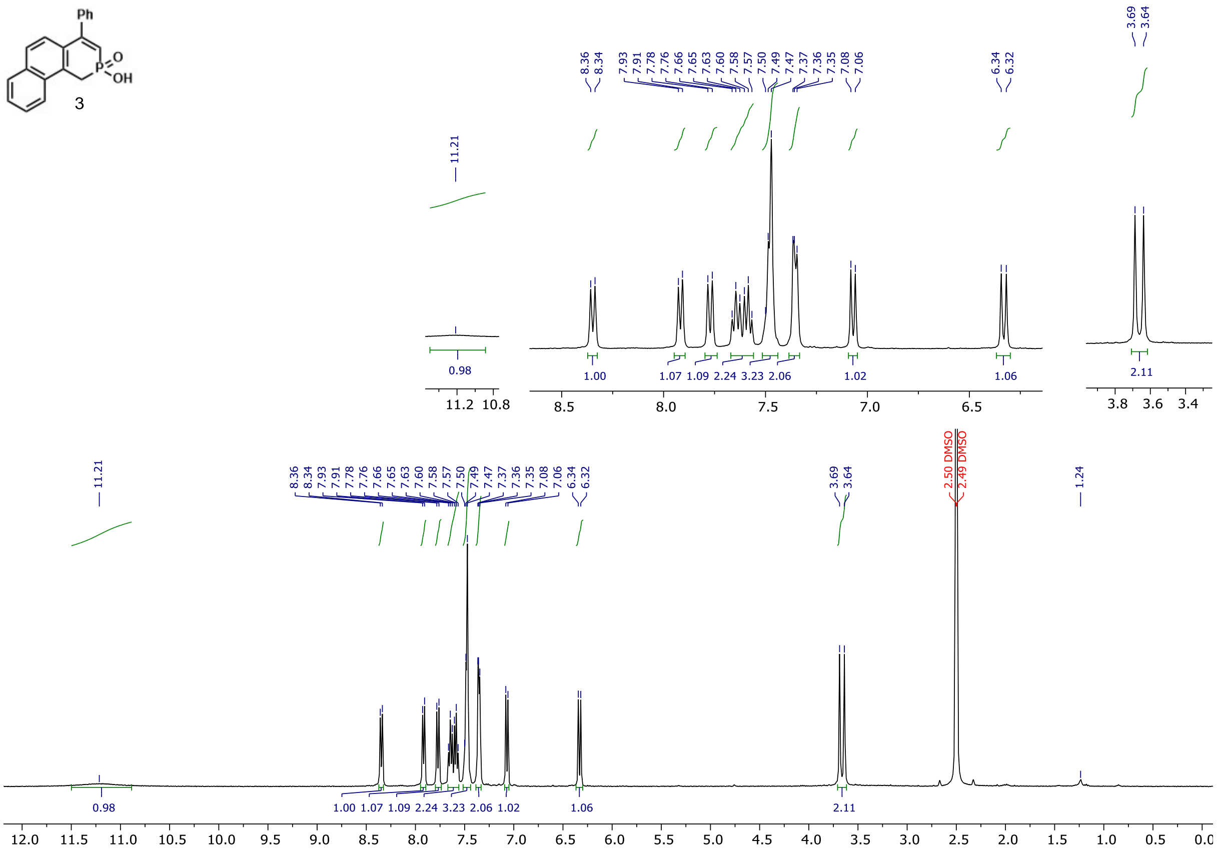

Figure S6 ^{1}H Spectrum of **3** (400 MHz, DMSO)

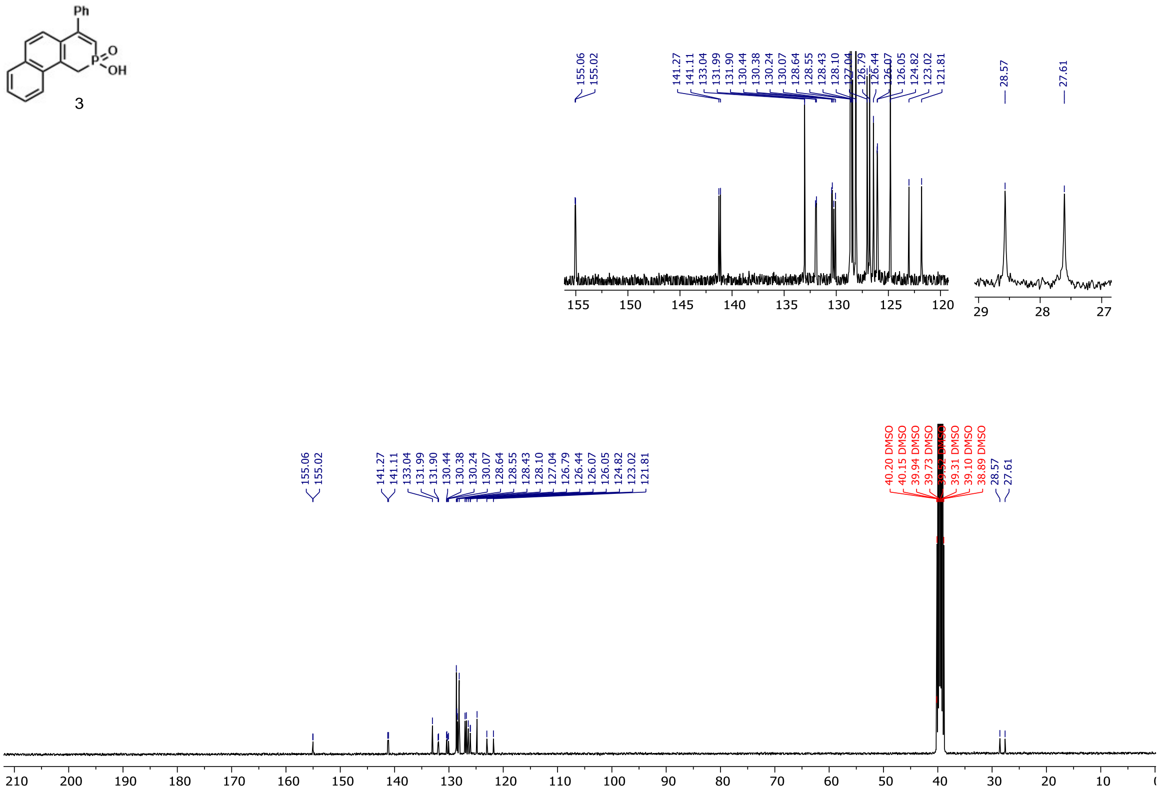

Figure S7 ^{13}C{^{1}H} Spectrum of **3** (101 MHz, DMSO)

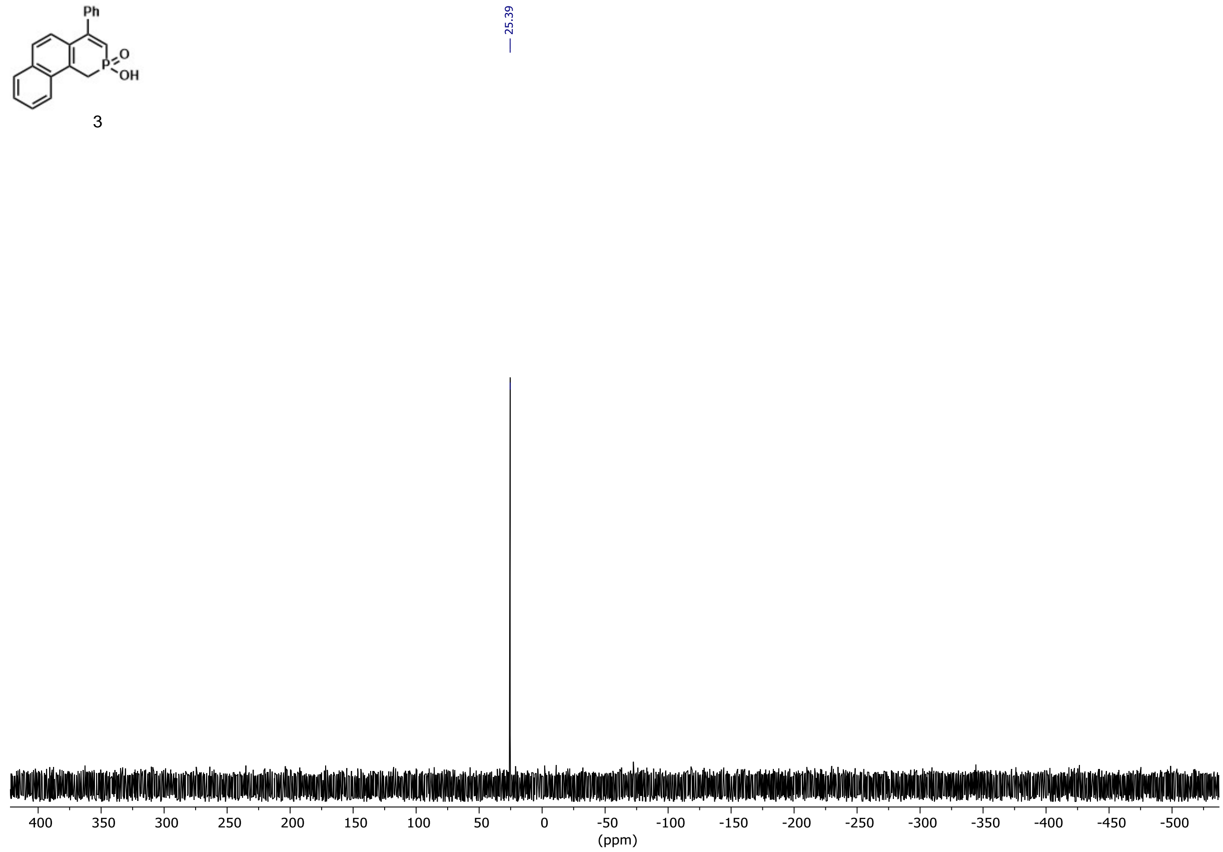

Figure S8 $^{31}P\{^{1}H\}$ Spectrum of **3** (162 MHz, DMSO)

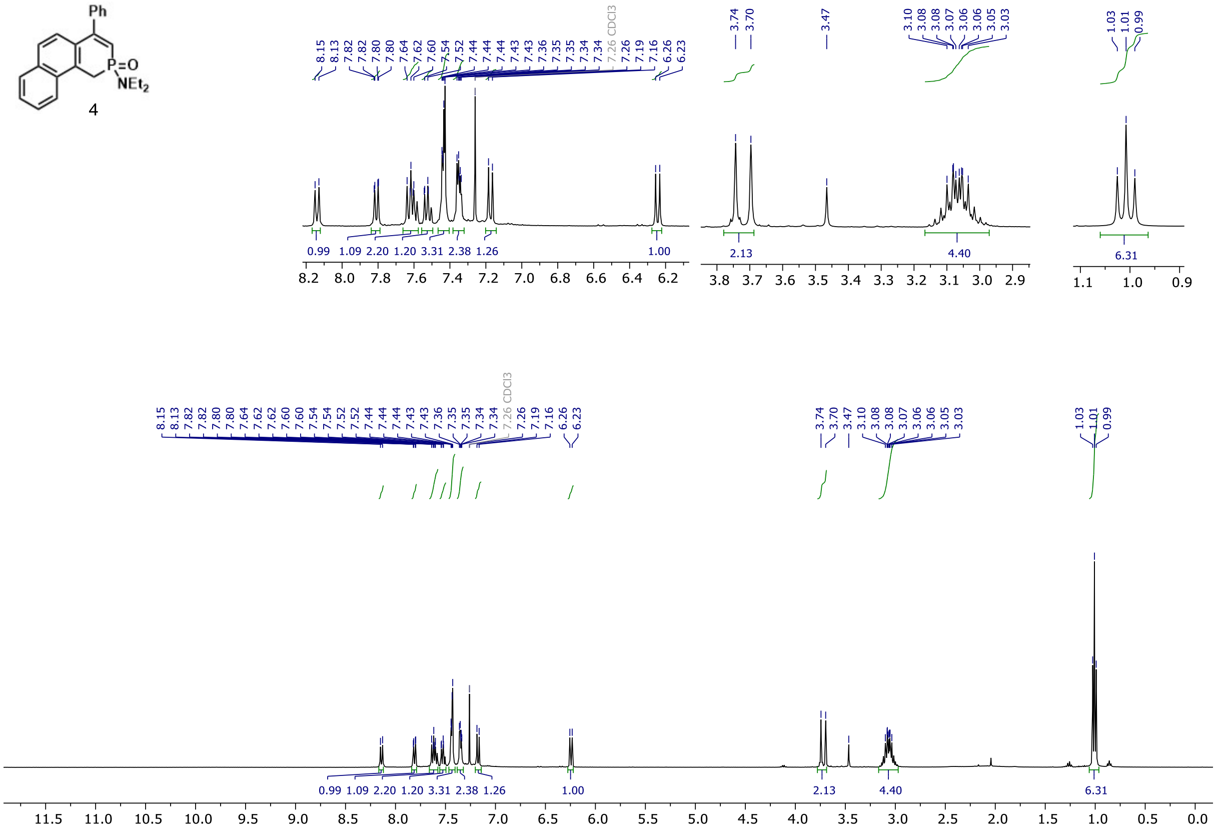

Figure S9 ^{1}H Spectrum of **4** (400 MHz, $CDCl_3$)

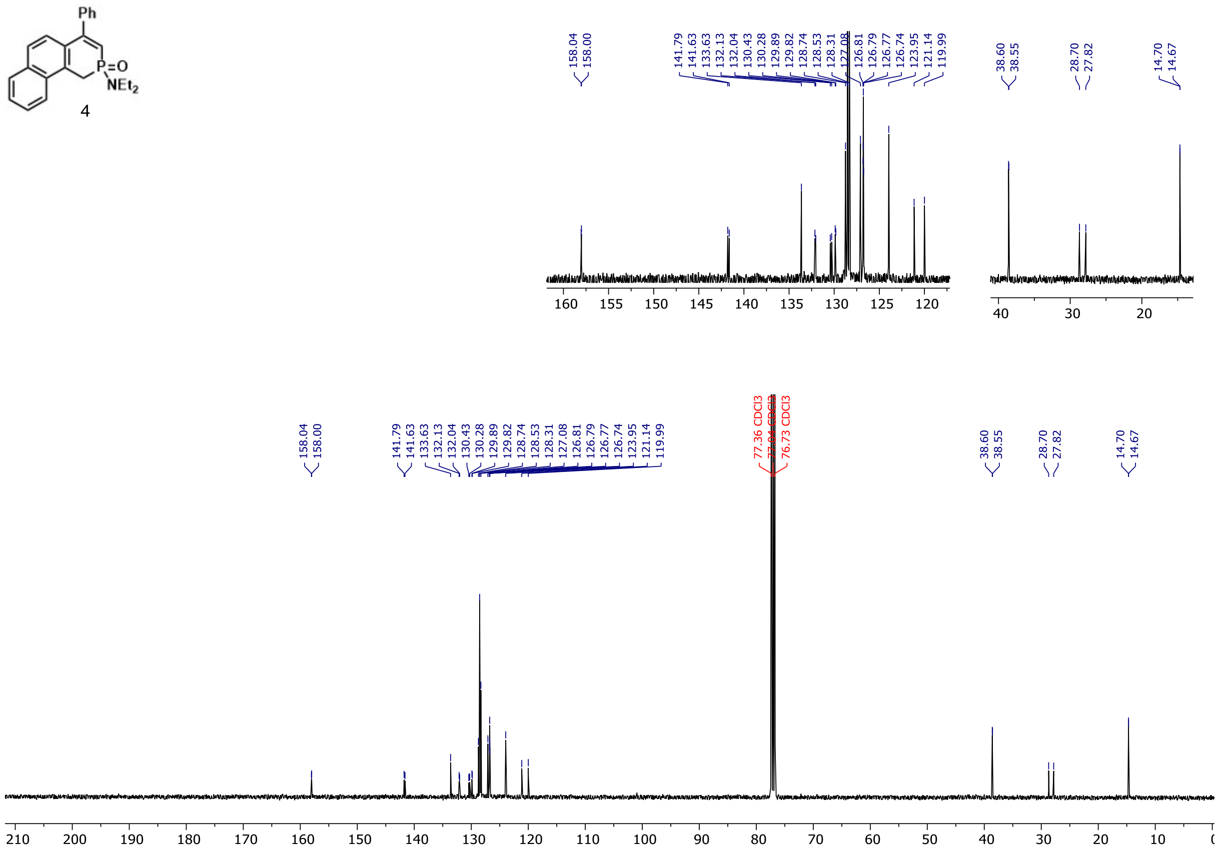

Figure S10 $^{13}C\{^1H\}$ Spectrum of **4** (101 MHz, $CDCl_3$)

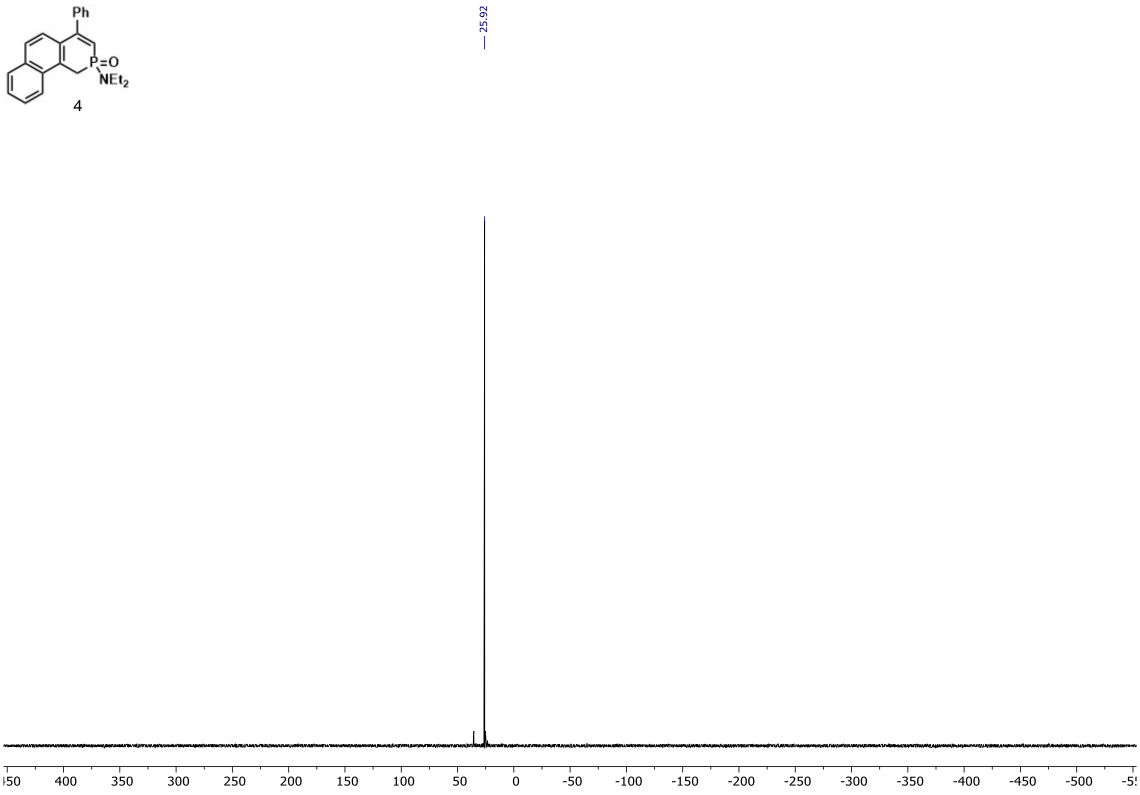

Figure S11 $^{31}P\{^1H\}$ Spectrum of **4** (162 MHz, $CDCl_3$)

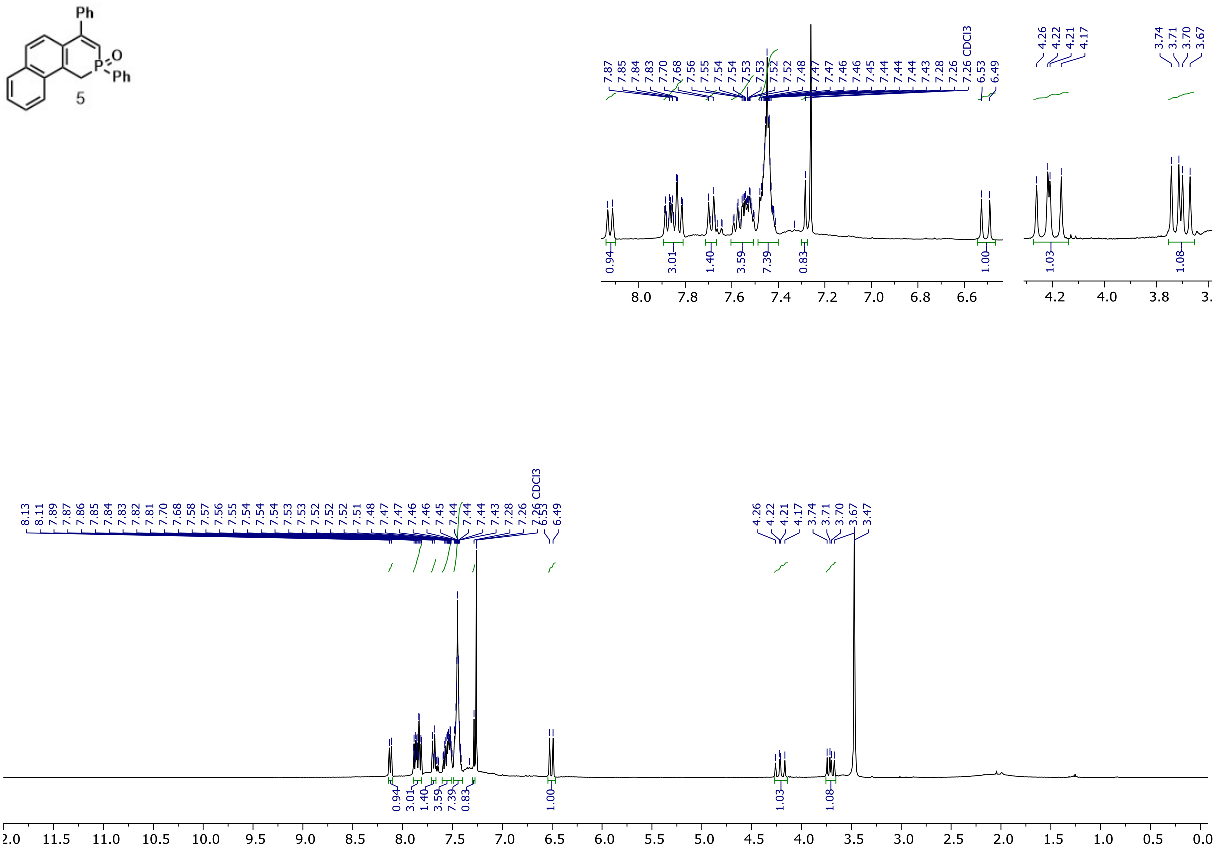

Figure S12 ^{1}H Spectrum of **5** (400 MHz, $CDCl_3$)

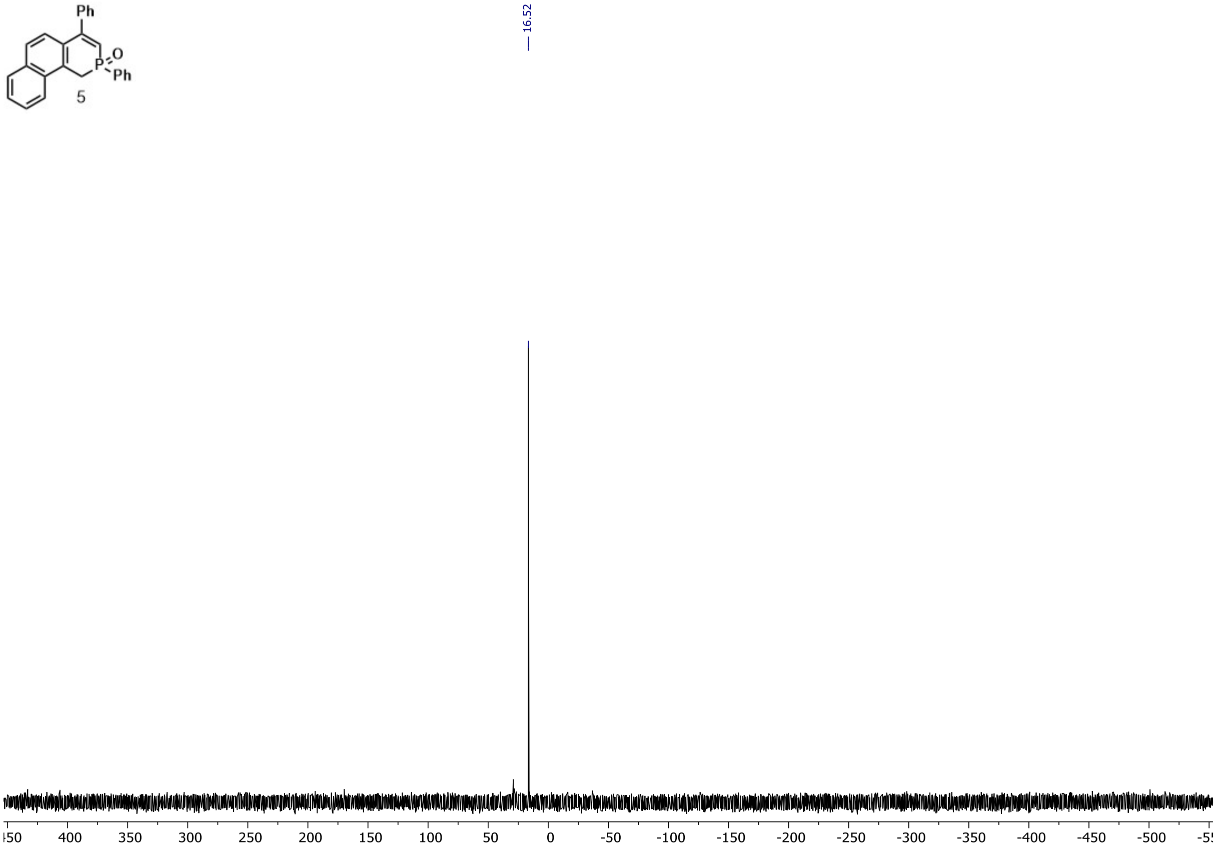

Figure S13 ^{31}P{^{1}H} Spectrum of **5** (162 MHz, $CDCl_3$)

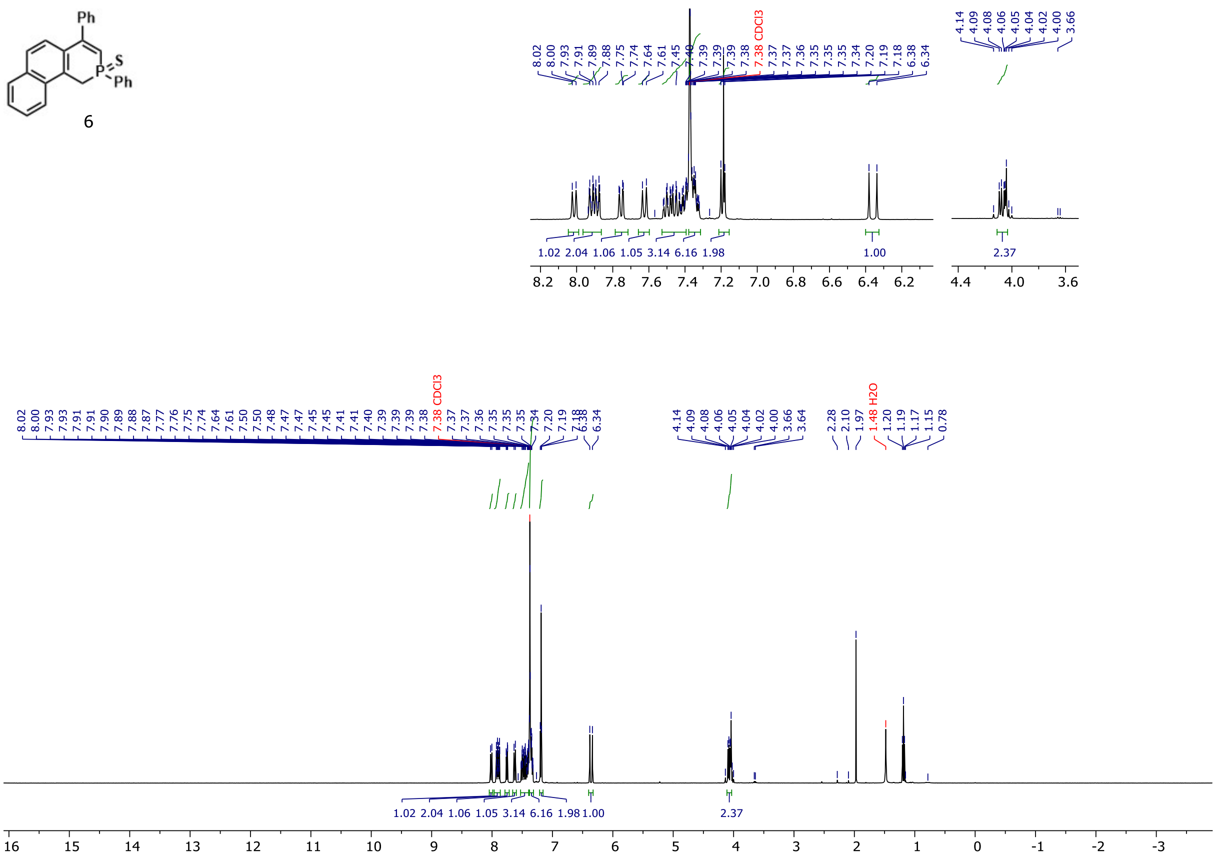

Figure S14 ^{1}H Spectrum of **6** (400 MHz, $CDCl_3$)

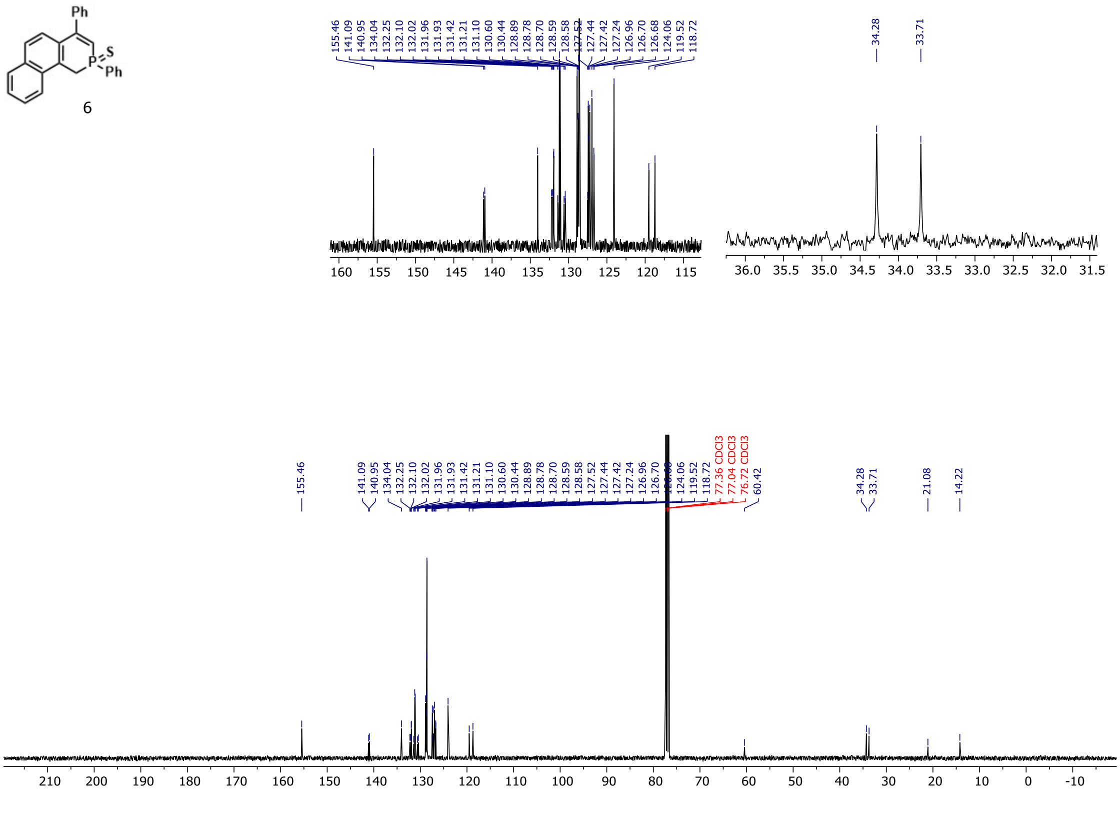

Figure S15 $^{13}C\{^{1}H\}$ Spectrum of **6** (101 MHz, $CDCl_3$)

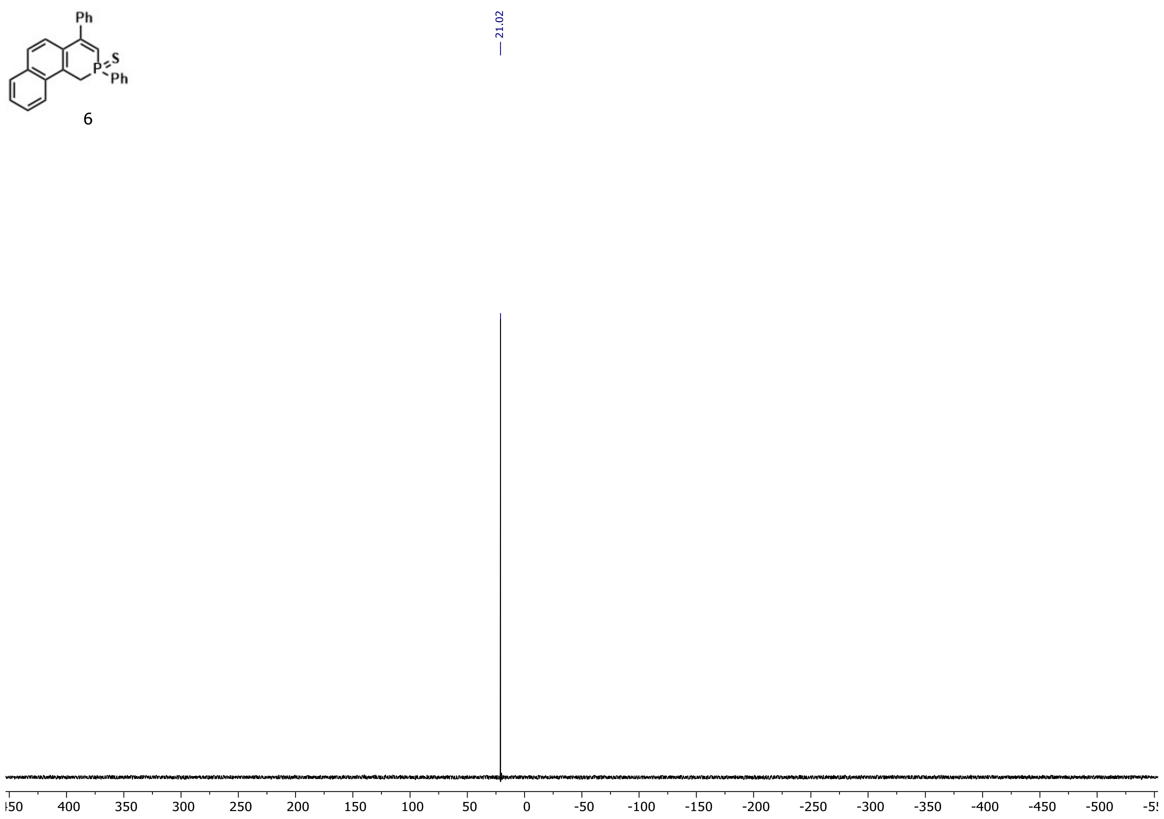

Figure S16 $^{31}P\{^1H\}$ Spectrum of **6** (162 MHz, $CDCl_3$)

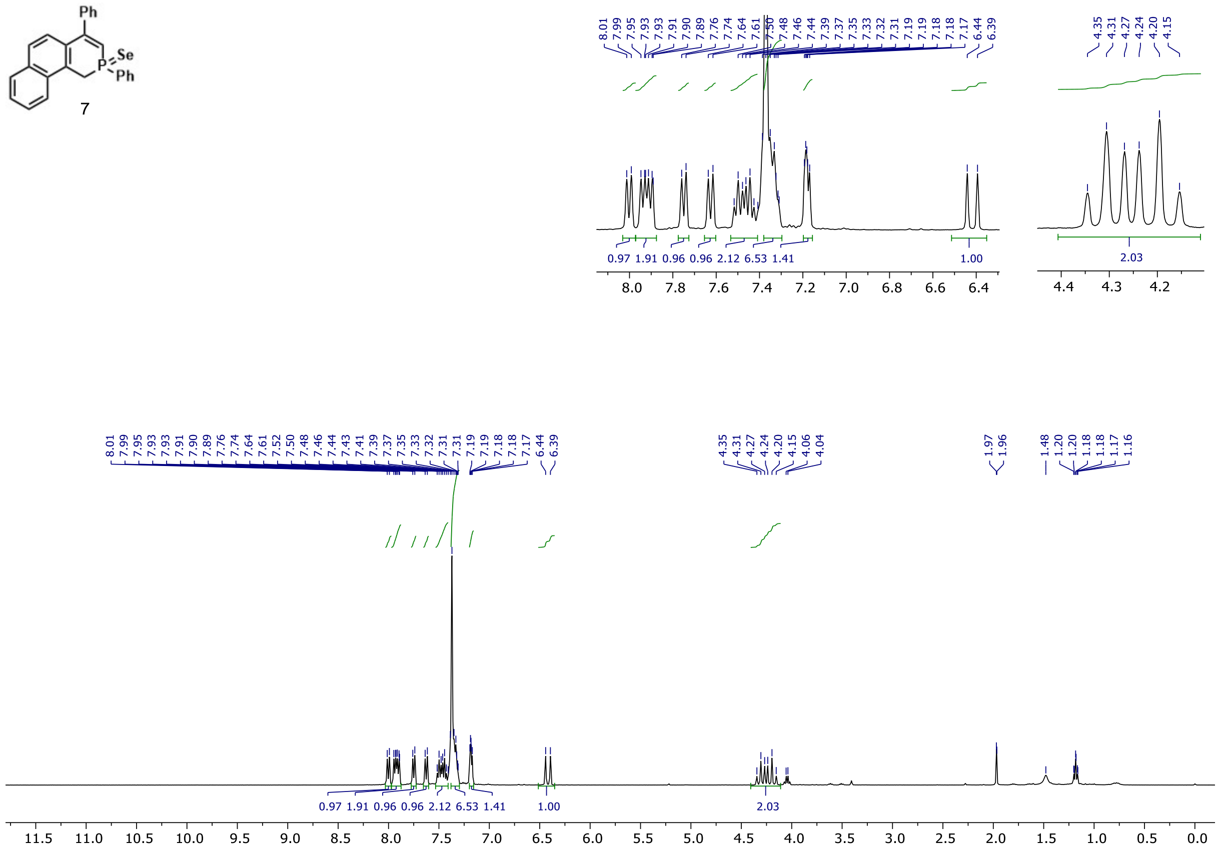

Figure S17 ^1H Spectrum of **7** (400 MHz, $CDCl_3$)

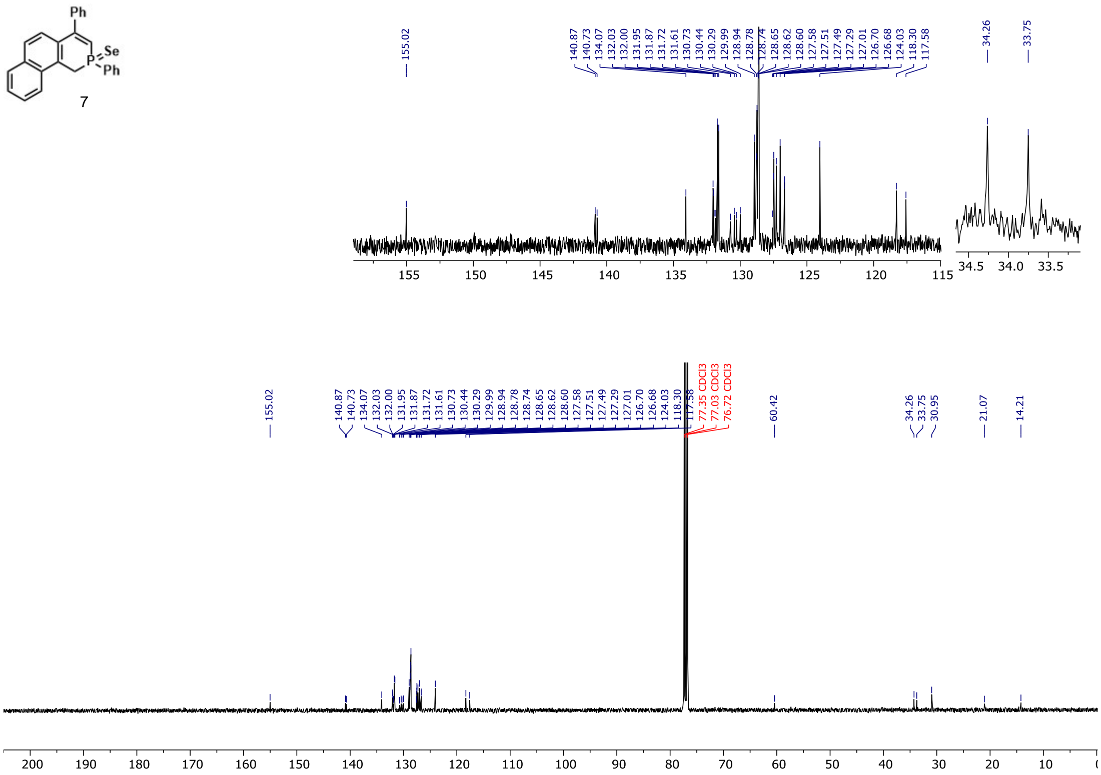

Figure S18 $^{13}C\{^{1}H\}$ Spectrum of **7** (101 MHz, $CDCl_3$)

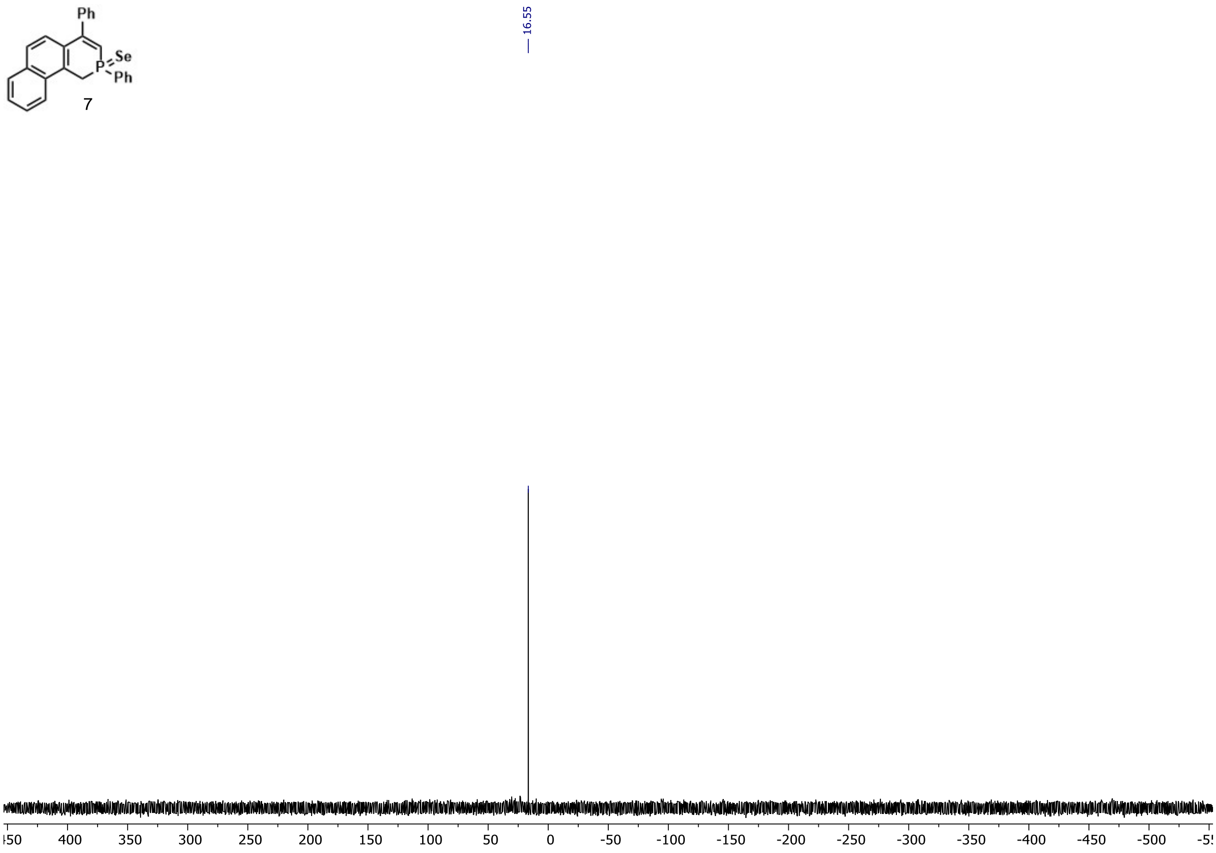

Figure S12 $^{31}P\{^{1}H\}$ Spectrum of **7** (162 MHz, $CDCl_3$)

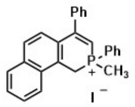

8

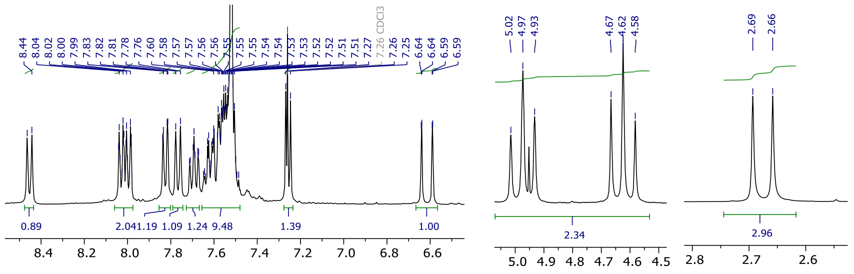

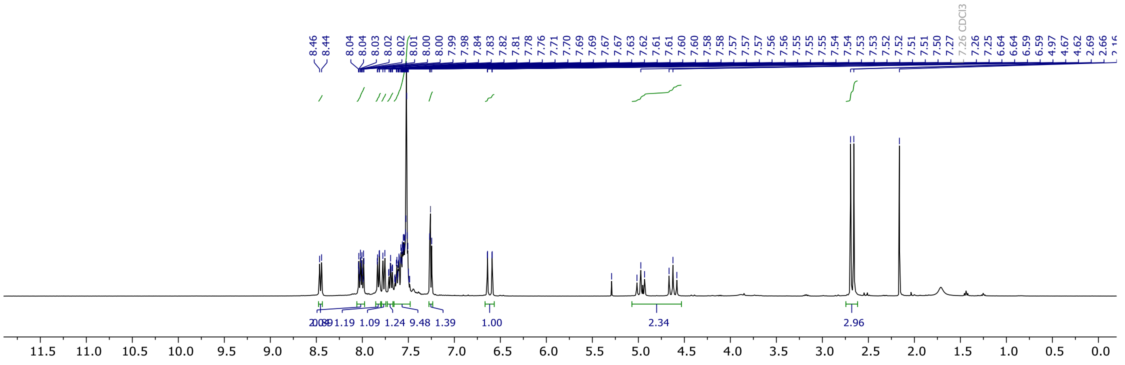

Figure S19 ^{1}H Spectrum of **8** (400 MHz, $CDCl_3$)

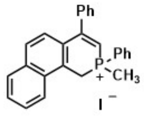

8

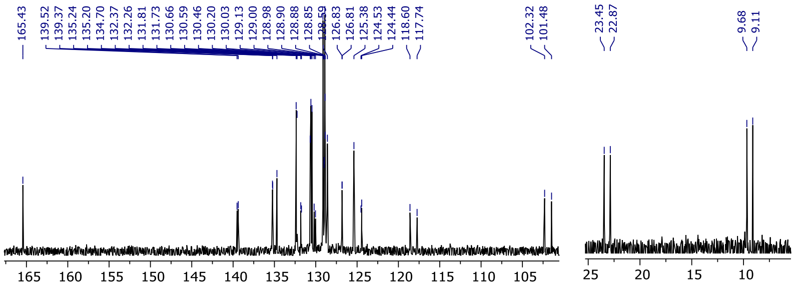

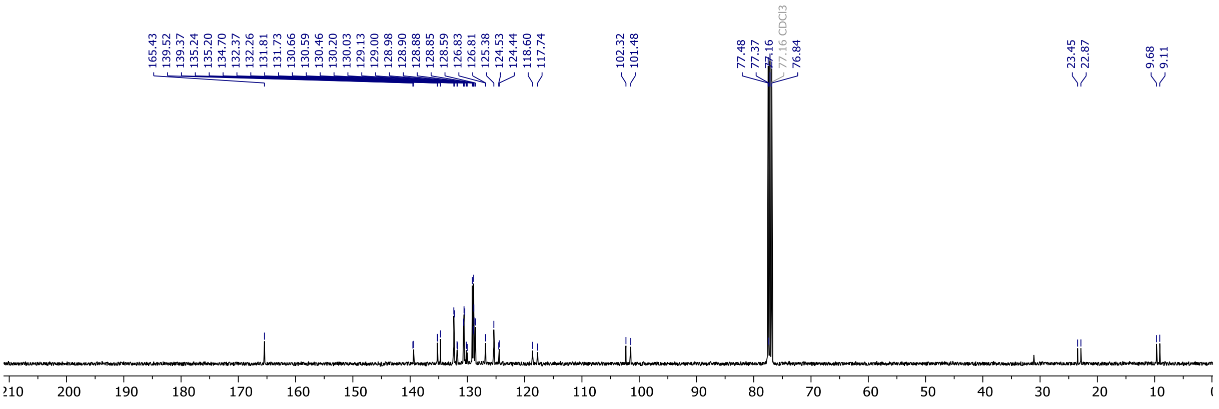

Figure S20 ^{13}C{^{1}H} Spectrum of **8** (400 MHz, $CDCl_3$)

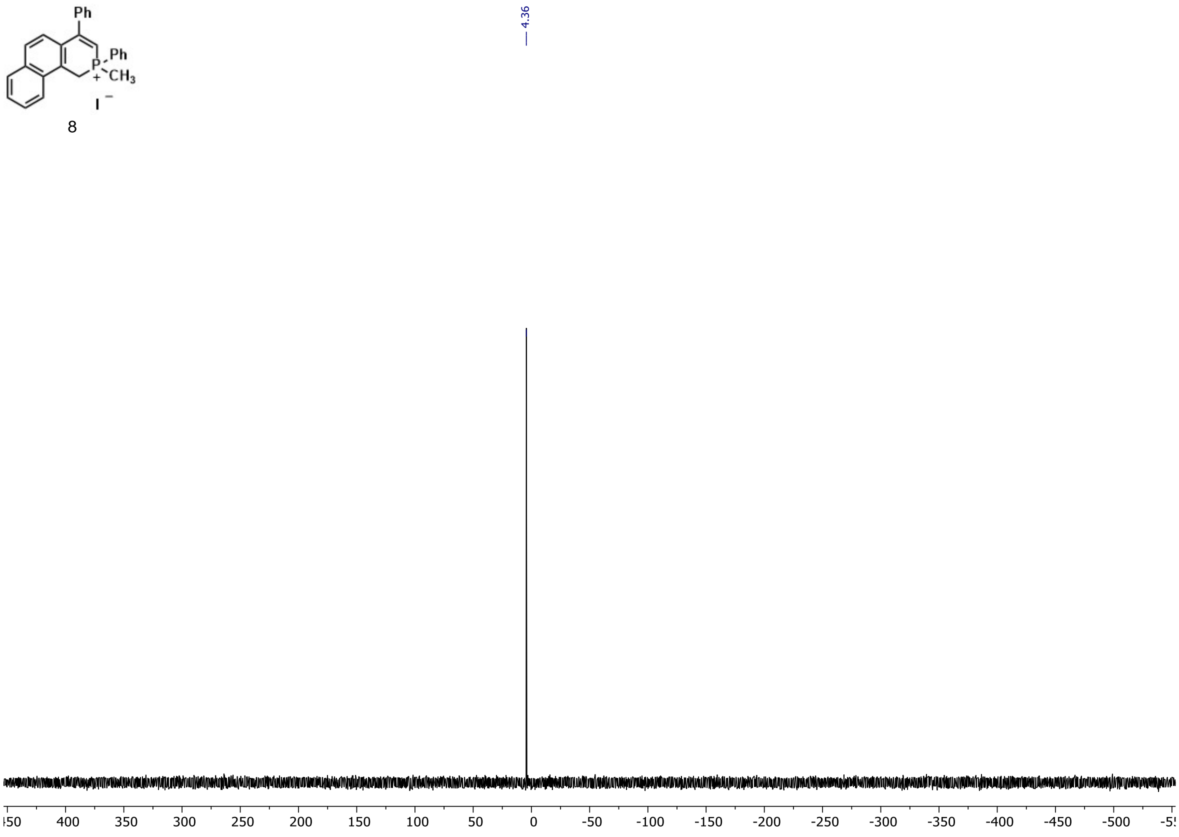

Figure S21 $^{31}P\{^{1}H\}$ Spectrum of **8** (162 MHz, $CDCl_3$)

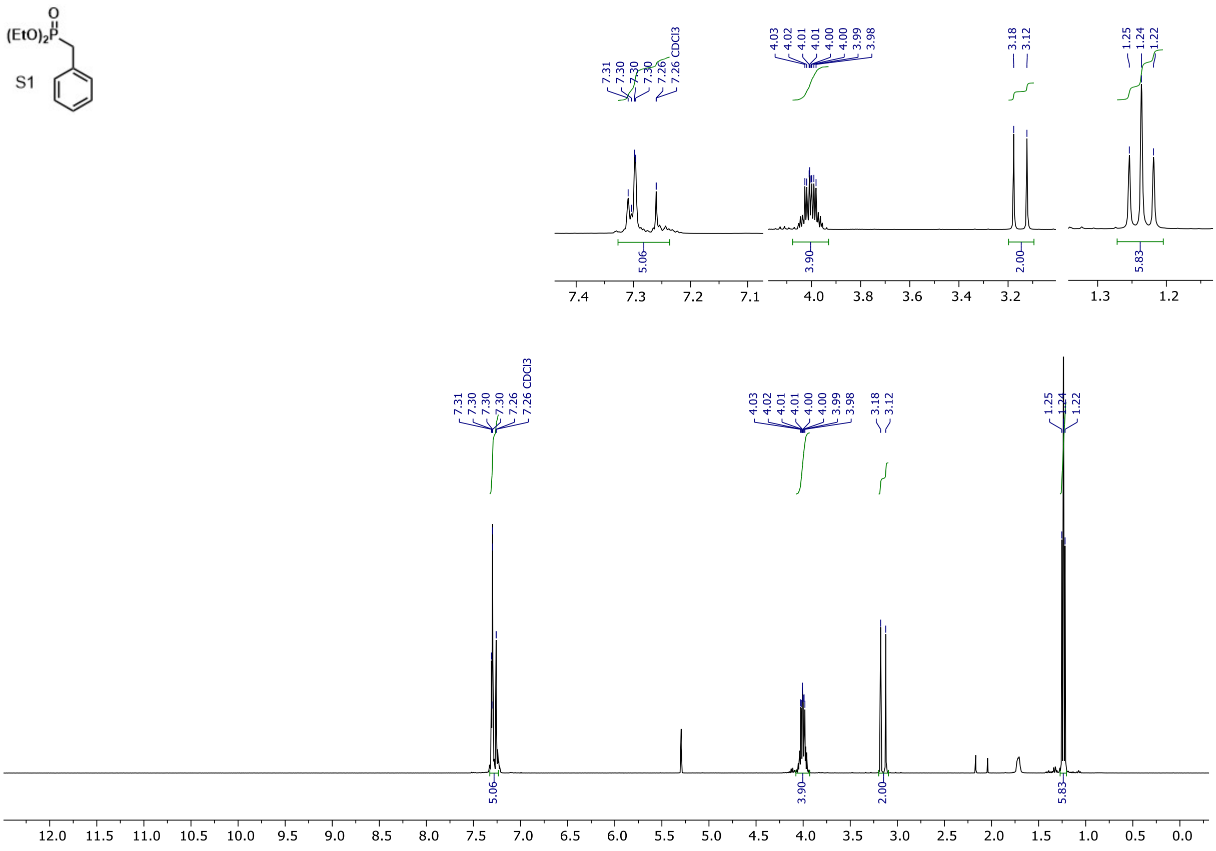

Figure S22 ^{1}H Spectrum of **S1** (400 MHz, $CDCl_3$)

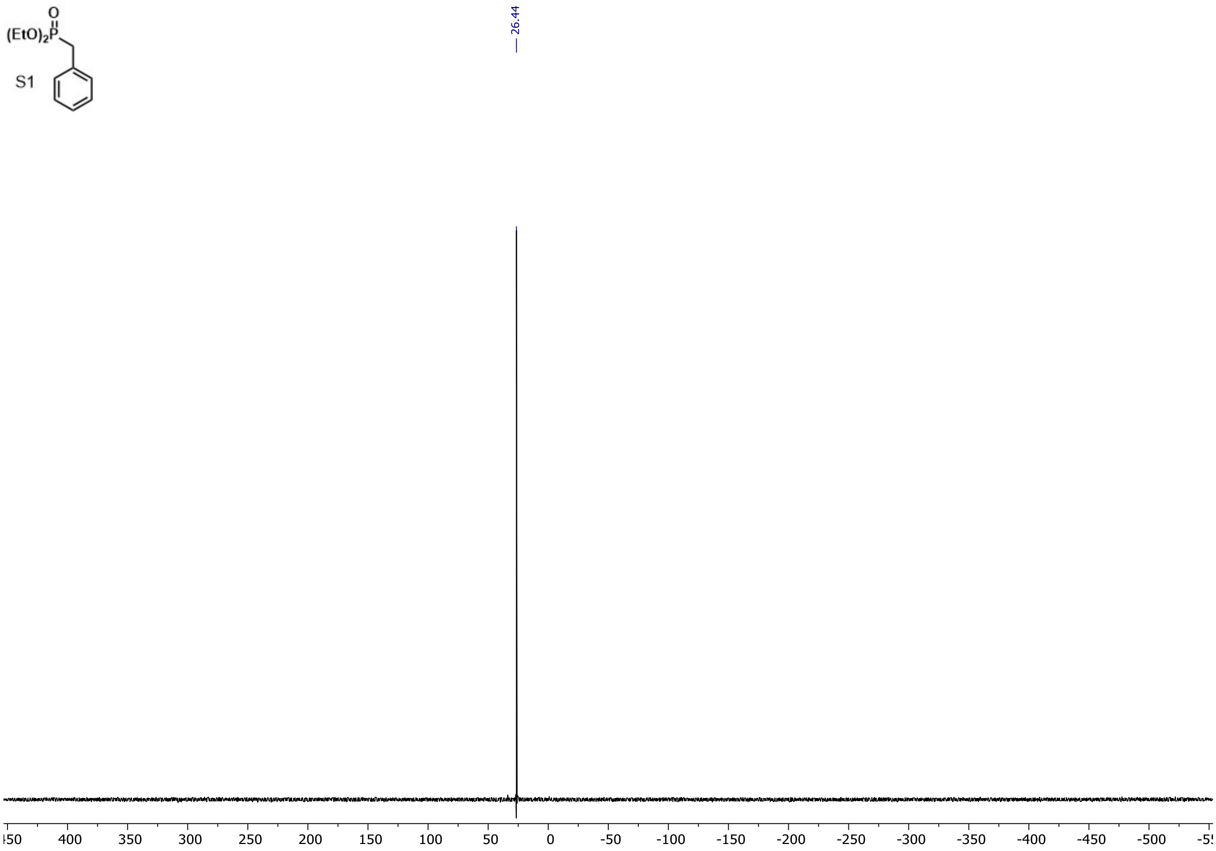

Figure S23 $^{31}P\{^1H\}$ Spectrum of **S1** (162 MHz, $CDCl_3$)

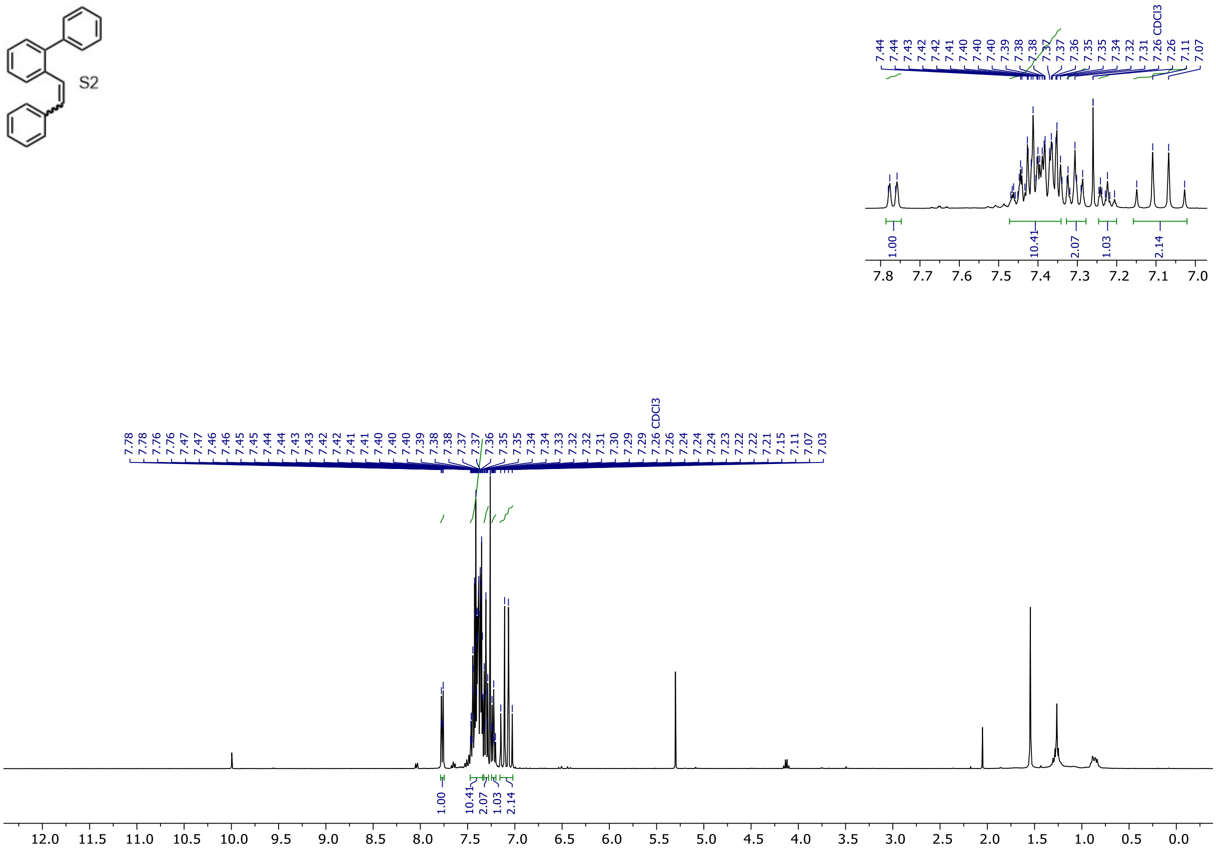

Figure S24 ^1H Spectrum of **S2** (400 MHz, $CDCl_3$)

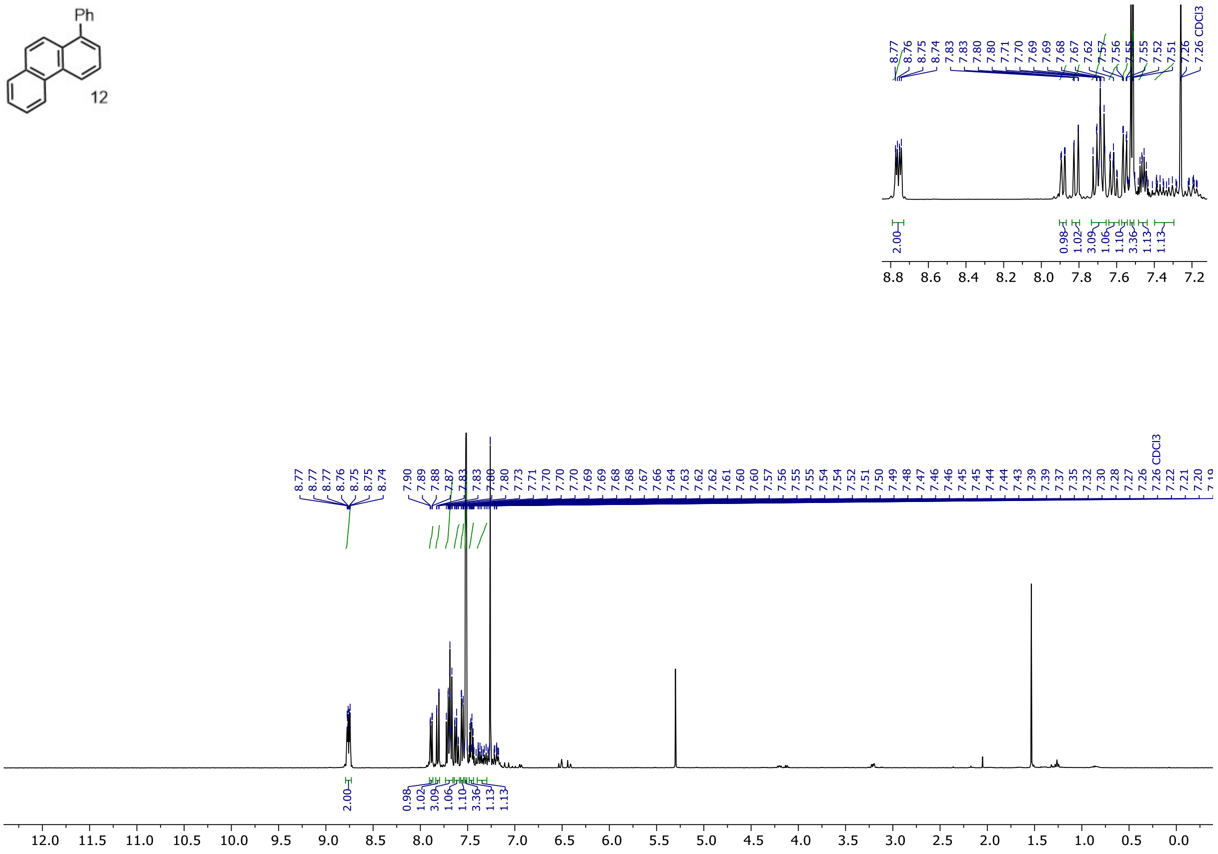

Figure S25 ^{1}H Spectrum of **12** (400 MHz, $CDCl_3$)

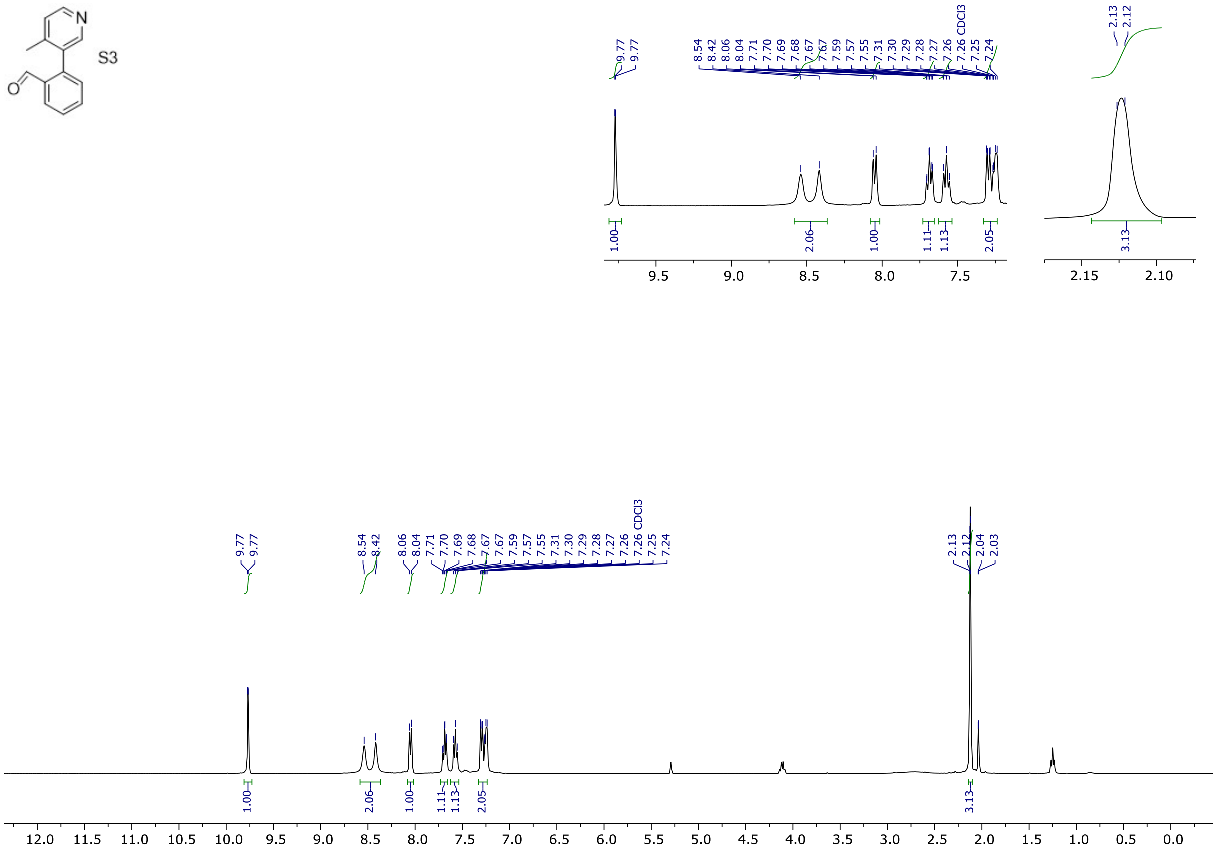

Figure S26 ^{1}H Spectrum of **S3** (400 MHz, $CDCl_3$)

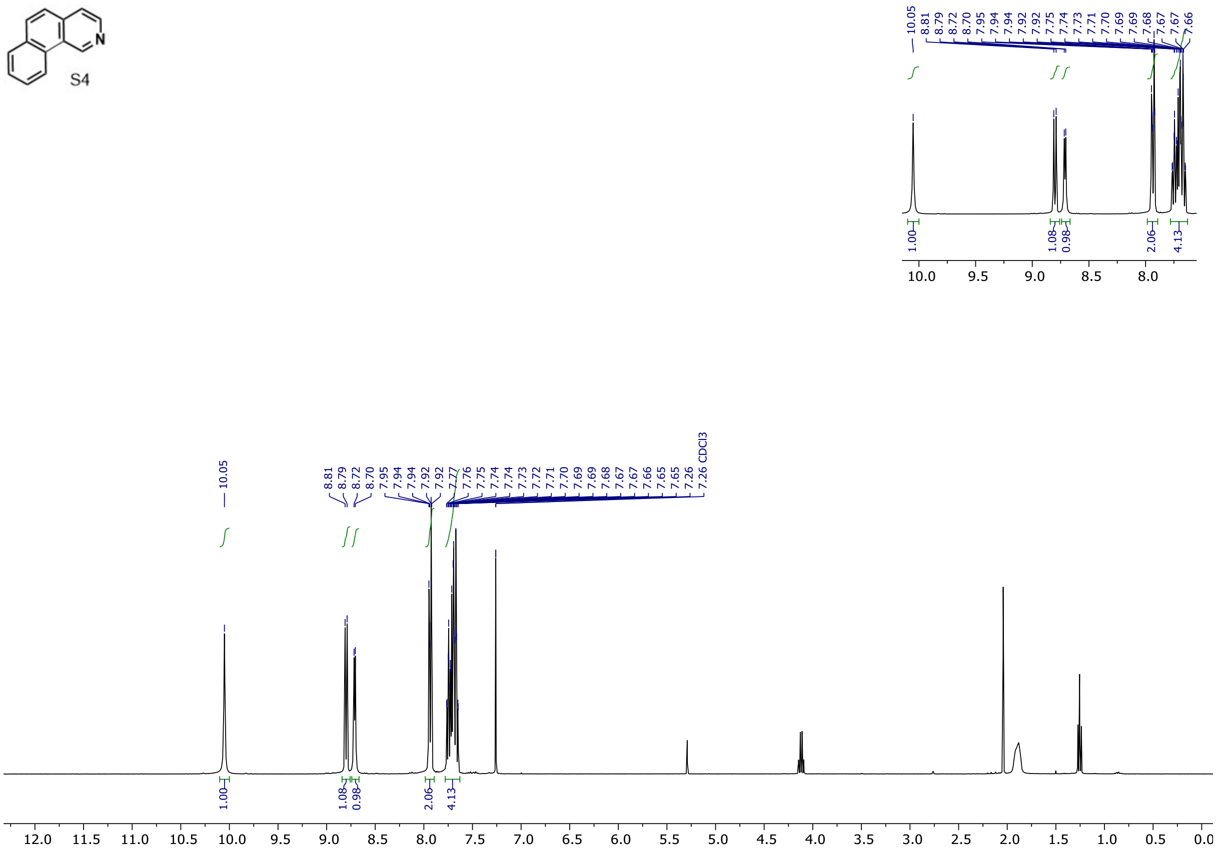

Figure S27 ^{1}H Spectrum of **S4** (400 MHz, $CDCl_3$)

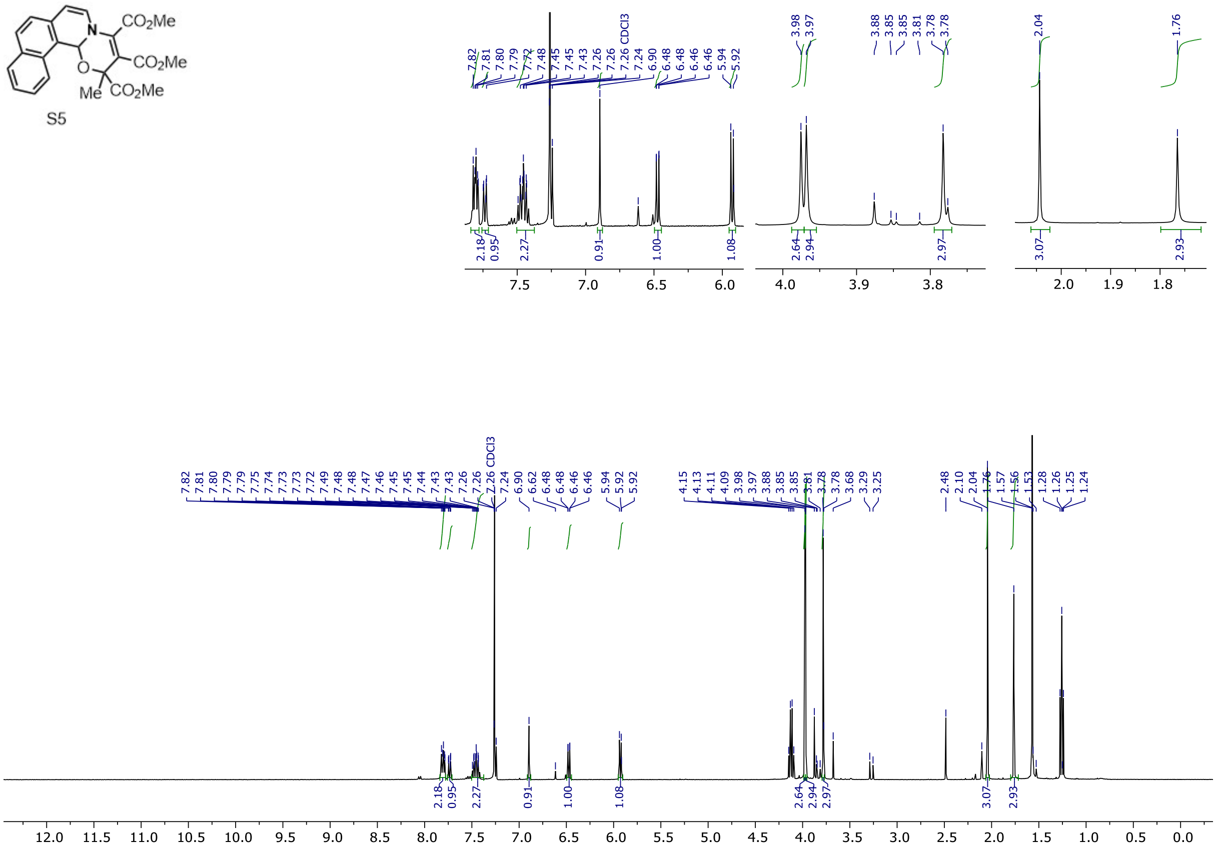

Figure S28 ^{1}H Spectrum of **S5** (400 MHz, $CDCl_3$)

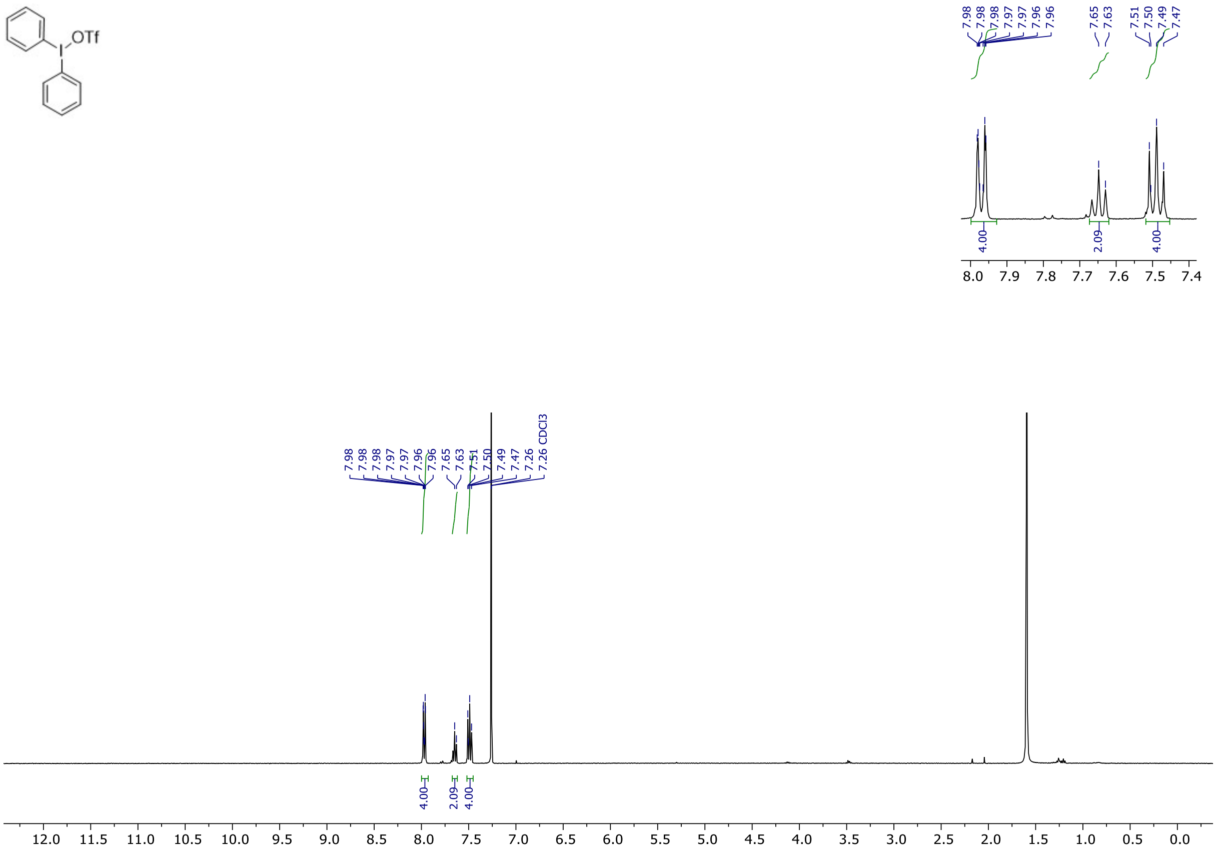

Figure S29 ^{1}H Spectrum of diphenyliodonium trifluoromethanesulfonate (400 MHz, $CDCl_3$)

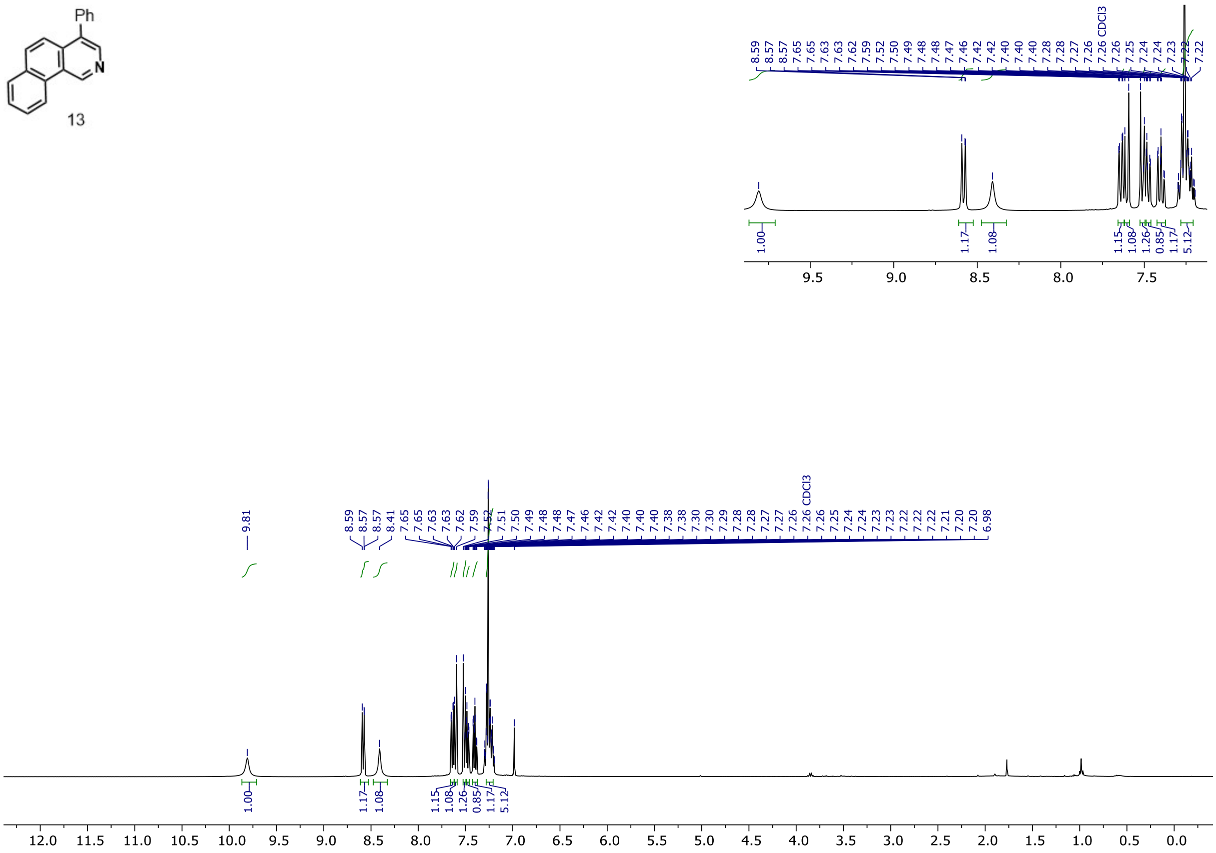

Figure S30 ^{1}H Spectrum of **13** (400 MHz, $CDCl_3$)

4. DFT Calculations

Optimization of the structures and calculation of HOMO/LUMO energies were performed with the Gaussian09 programme[10]. B3LYP[11,12] was used as the functional in combination with the 6-311++G(d,p) basis set. The vibrational analysis showed that all structures correspond to the local minima (zero imaginary frequencies) in the potential energy surface.. The Cartesian coordinates of all optimized structures are following.

*4-Phenylbenzo[h]isophosphinoline (**1**)*

```
Total energy =                                  -1073.41698999 a.u.
Number of imaginary frequencies =               0
Zero-point correction=                          0.257685
(Hartree/Particle)Thermal correction to Energy=                  0.272594
Thermal correction to Enthalpy=                 0.273538
Thermal correction to Gibbs Free Energy=        0.214378
Sum of electronic and zero-point Energies=           -1073.159305
Sum of electronic and thermal Energies=              -1073.144396
Sum of electronic and thermal Enthalpies=            -1073.143452
Sum of electronic and thermal Free Energies=         -1073.202612

Charge =  0 Multiplicity = 1 C                     -1.18159  -2.07269
0.21873
 C                  -2.44661  -1.41533   0.12002
 C                  -2.48705   0.00208   0.00287
 C                  -1.23372   0.76447  -0.01633
 C                   0.00928   0.05465   0.04135
 C                  -0.01805  -1.37474   0.17667
 C                  -1.26       2.17177  -0.08011
 P                   0.07636   3.25615  -0.09681
 C                   1.3937    2.10414  -0.04841
 C                   1.2735    0.72814  -0.00335
 C                  -3.64847  -2.15905   0.14318
 C                  -4.87207  -1.53352   0.04953
 C                  -4.92286  -0.13424  -0.07098
 C                  -3.76008   0.61044  -0.09223
 C                   2.55004  -0.05939  -0.01187
 C                   3.41604  -0.01831   1.08707
 C                   4.6267   -0.7096    1.06474
 C                   4.99224  -1.4464   -0.05992
 C                   4.13958  -1.48888  -1.16256
 C                   2.92733  -0.80313  -1.13763
 H                  -1.16574  -3.15186   0.33016
 H                   0.92178  -1.90261   0.25907
 H                  -2.22574   2.66073  -0.11399
 H                   2.3979    2.51655  -0.0784
 H                  -3.58888  -3.23851   0.2351
 H                  -5.78819  -2.11273   0.06678
 H                  -5.88084   0.3673   -0.14905
 H                  -3.84645   1.68384  -0.18965
 H                   3.1348    0.55349   1.96459
 H                   5.28325  -0.67137   1.92695
 H                   5.93494  -1.98152  -0.07874
 H                   4.41994  -2.05296  -2.04526
 H                   2.27043  -0.83499  -2.00001
```

*2-Ethoxy-4-phenyl-1H-benzo[h]isophosphinoline 2-oxide (**2**)*

```
Total energy =                                  -1303.80233068 a.u.
Number of imaginary frequencies =               0
Zero-point correction=                          0.344347 (Hartree/Particle)
Thermal correction to Energy=                   0.365077
Thermal correction to Enthalpy=                 0.366022
Thermal correction to Gibbs Free Energy=        0.292857
Sum of electronic and zero-point Energies=           -1303.457984
Sum of electronic and thermal Energies=              -1303.437253
Sum of electronic and thermal Enthalpies=            -1303.436309
Sum of electronic and thermal Free Energies=         -1303.509474

Charge =  0 Multiplicity = 1
 C                    -2.31178  -2.2485    0.58222
 C                    -3.23412  -1.22609   0.25576
 C                    -2.73535   0.05954  -0.1337
 C                    -1.32015   0.28662  -0.1751
 C                    -0.43857  -0.76018   0.11029
 C                    -0.96574  -2.02476   0.50067
 C                    -0.79822   1.65331  -0.56721
 P                     0.73866   2.11059   0.29043
 C                     1.66844   0.58891   0.15361
 C                     1.04398  -0.60535   0.07466
 C                    -4.63342  -1.45407   0.30645
 C                    -5.52462  -0.46421  -0.02583
 C                    -5.0441    0.80237  -0.42566
 C                    -3.69402   1.05634  -0.47482
 C                     1.89069  -1.8329   -0.03642
 C                     2.89418  -2.08897   0.90614
 C                     3.72051  -3.20427   0.77989
 C                     3.56056  -4.07607  -0.29484
 C                     2.56416  -3.83006  -1.23975
 C                     1.73092  -2.72275  -1.10798
 H                    -2.68687  -3.21686   0.89575
 H                    -0.27844  -2.8206    0.75391
 H                    -1.51638   2.44309  -0.35164
 H                     2.75125   0.64164   0.13945
 H                    -4.98681  -2.43324   0.61232
 H                    -6.59185  -0.64972   0.01558
 H                    -5.74676   1.58313  -0.69412
 H                    -3.36742   2.03763  -0.79029
 H                     3.01456  -1.41727   1.74859
 H                     4.48626  -3.39246   1.52414
 H                     4.2045   -4.94243  -0.39525
 H                     2.43677  -4.50069  -2.08223
 H                     0.9581   -2.53829  -1.84583
 H                    -0.57911   1.69153  -1.64137
 C                     2.14201   4.31518  -0.35349
 H                     1.95224   5.05428  -1.1338
 H                     1.7089    4.6695    0.58419
 C                     3.63005   4.04765  -0.2044
 H                     3.82474   3.33959   0.60516
 H                     4.05065   3.64903  -1.1307
 H                     4.14959   4.98033   0.03589
 O                     0.60551   2.68827   1.6602
 O                     1.42745   3.1283   -0.78384
```

*2-Hydroxy-4-phenyl-1H-benzo[h]isophosphinoline 2-oxide (**3**)*

```
Total energy =                                  -1225.16603504 a.u.
Number of imaginary frequencies =               0
Zero-point correction=                          0.287765 (Hartree/Particle)
Thermal correction to Energy=                   0.305648
Thermal correction to Enthalpy=                 0.306593
Thermal correction to Gibbs Free Energy=        0.241341
Sum of electronic and zero-point Energies=           -1224.878270
Sum of electronic and thermal Energies=              -1224.860387
Sum of electronic and thermal Enthalpies=            -1224.859443
Sum of electronic and thermal Free Energies=         -1224.924694
```

Charge = 0 Multiplicity = 1

C	-1.19609	-2.40411	0.6834
C	-2.42927	-1.81106	0.32221
C	-2.43888	-0.4527	-0.13371
C	-1.20698	0.27674	-0.20672
C	-0.00222	-0.35654	0.11237
C	-0.02684	-1.70504	0.57066
C	-1.22227	1.72024	-0.66627
C	1.46012	1.67365	0.06149
C	1.31934	0.33079	0.04117
C	-3.64583	-2.53634	0.40252
C	-4.83782	-1.96195	0.03681
C	-4.85714	-0.62821	-0.42766
C	-3.69597	0.10319	-0.50705
C	2.55629	-0.50364	-0.04685
C	3.60299	-0.32237	0.86554
C	4.77875	-1.06249	0.75396
C	4.92844	-1.98837	-0.27614
C	3.89218	-2.17585	-1.19095
C	2.71281	-1.44611	-1.07313
H	-1.18898	-3.42562	1.04823
H	0.90476	-2.17801	0.85026
H	-2.18284	2.19896	-0.48166
H	2.44383	2.12719	0.02081
H	-3.61446	-3.56092	0.75797
H	-5.76141	-2.52573	0.10137
H	-5.79723	-0.17568	-0.72222
H	-3.75273	1.11953	-0.87182
H	3.48587	0.39133	1.67318
H	5.57563	-0.91627	1.4745
H	5.84407	-2.56192	-0.36517
H	4.00316	-2.89028	-1.99898
H	1.91164	-1.59757	-1.78776
H	-1.02254	1.78579	-1.74311
P	0.03488	2.74261	0.15803
O	-0.32157	3.31302	1.48939
O	0.34937	3.91043	-0.95249
H	0.25601	4.78343	-0.55109

*2-(Diethylamino)-4-phenyl-1H-benzo[h]isophosphinoline 2-oxide (**4**)*

```
Total energy =                                  -1362.54854844 a.u.
Number of imaginary frequencies =               0
Zero-point correction=                          0.413112 (Hartree/Particle)
Thermal correction to Energy=                   0.436684
Thermal correction to Enthalpy=                 0.437628
Thermal correction to Gibbs Free Energy=         0.358890
Sum of electronic and zero-point Energies=           -1362.135436
```

```
Sum of electronic and thermal Energies=            -1362.111864
Sum of electronic and thermal Enthalpies=          -1362.110920
Sum of electronic and thermal Free Energies=       -1362.189658

Charge =  0 Multiplicity = 1
 C                  -3.12386   1.70062  -0.52667
 C                  -3.66571   0.42227  -0.25236
 C                  -2.77706  -0.65681   0.06636
 C                  -1.36349  -0.41879   0.09524
 C                  -0.86809   0.86408  -0.14606
 C                  -1.77479   1.91353  -0.46551
 C                  -0.41295  -1.55328   0.42679
 C                   1.53336   0.23404  -0.36002
 C                   0.5894    1.17206  -0.1396
 C                  -5.06591   0.19675  -0.28714
 C                  -5.59211  -1.04032  -0.00942
 C                  -4.72744  -2.10803   0.31721
 C                  -3.36574  -1.92228   0.3515
 C                   1.02292   2.58096   0.10558
 C                   1.90815   3.219    -0.77273
 C                   2.3541    4.5147   -0.51814
 C                   1.92541   5.19222   0.62131
 C                   1.04183   4.56794   1.50188
 C                   0.58877   3.27749   1.24278
 H                  -3.79493   2.51276  -0.78529
 H                  -1.37902   2.89741  -0.67991
 H                  -0.882    -2.52321   0.27845
 H                   2.57986   0.51014  -0.35824
 H                  -5.71662   1.02794  -0.53807
 H                  -6.66373  -1.20098  -0.03946
 H                  -5.14093  -3.0853    0.53965
 H                  -2.74113  -2.7671    0.60679
 H                   2.23357   2.69959  -1.66697
 H                   3.0322    4.99579  -1.21425
 H                   2.27292   6.19965   0.82051
 H                   0.70592   5.08648   2.39305
 H                  -0.09878   2.79976   1.93182
 H                  -0.10389  -1.48006   1.47617
 P                   1.08396  -1.49169  -0.62913
 O                   0.86983  -1.86661  -2.06396
 N                   2.28735  -2.40723   0.16868
 C                   3.45676  -2.77585  -0.66945
 H                   4.10764  -3.38368  -0.03714
 H                   3.13092  -3.40772  -1.50575
 C                   4.26569  -1.59764  -1.20604
 H                   3.69402  -1.00271  -1.92114
 H                   4.60907  -0.9501   -0.39498
 H                   5.1447   -1.97837  -1.73357
 C                   1.93344  -3.47853   1.12051
 H                   2.05538  -4.45637   0.63442
 H                   0.87994  -3.40756   1.38702
 C                   2.76866  -3.42103   2.40171
 H                   3.83817  -3.49728   2.19068
 H                   2.59777  -2.48056   2.93106
 H                   2.50141  -4.24821   3.06671
```

*2,4-Diphenyl-1H-benzo[h]isophosphinoline 2-oxide (**5**)*

```
Total energy =                                   -1380.99610921 a.u.
Number of imaginary frequencies =                0
Zero-point correction=                           0.364121 (Hartree/Particle)
```

```
Thermal correction to Energy=                  0.385535
Thermal correction to Enthalpy=                0.386479
Thermal correction to Gibbs Free Energy=       0.312145
Sum of electronic and zero-point Energies=          -1380.631988
Sum of electronic and thermal Energies=             -1380.610574
Sum of electronic and thermal Enthalpies=           -1380.609630
Sum of electronic and thermal Free Energies=        -1380.683964

Charge =  0 Multiplicity = 1
 C                  -3.7289   -0.15152  -0.24188
 C                  -3.49072  -1.5246    0.00578
 C                  -2.14045  -1.97769   0.16329
 C                  -1.06291  -1.03685   0.06158
 C                  -1.33864   0.31782  -0.14433
 C                  -2.69116   0.73591  -0.30392
 C                   0.3648   -1.51185   0.21356
 C                   1.00899   1.05964  -0.59003
 C                  -0.27084   1.34912  -0.26781
 C                  -4.56102  -2.45078   0.10101
 C                  -4.32709  -3.77953   0.35478
 C                  -3.00107  -4.23505   0.52478
 C                  -1.94276  -3.36284   0.42962
 C                  -0.63286   2.78364  -0.05114
 C                  -0.33413   3.74778  -1.02194
 C                  -0.62308   5.09313  -0.8002
 C                  -1.20762   5.49595   0.39854
 C                  -1.50922   4.54392   1.37247
 C                  -1.23208   3.19859   1.14681
 H                  -4.74912   0.18991  -0.38077
 H                  -2.89559   1.78026  -0.49717
 H                   0.47242  -2.5711   -0.01451
 H                   1.73455   1.85752  -0.70416
 H                  -5.5742   -2.08522  -0.02948
 H                  -5.15265  -4.4785    0.42557
 H                  -2.81532  -5.28339   0.72935
 H                  -0.9432   -3.75089   0.56867
 H                   0.11219   3.43613  -1.95946
 H                  -0.3938    5.82484  -1.5668
 H                  -1.42998   6.54266   0.57244
 H                  -1.96053   4.84959   2.30983
 H                  -1.47234   2.46412   1.90745
 H                   0.70649  -1.36601   1.24597
 P                   1.54501  -0.63077  -0.88104
 O                   1.57924  -1.09979  -2.30645
 C                   3.18086  -0.78719  -0.07367
 C                   4.19825  -1.41125  -0.80282
 C                   3.4463   -0.31511   1.21805
 C                   5.46483  -1.56931  -0.24172
 H                   3.98284  -1.76139  -1.80598
 C                   4.71263  -0.47409   1.77545
 H                   2.67173   0.18695   1.78846
 C                   5.72226  -1.10312   1.04597
 H                   6.24979  -2.05397  -0.81144
 H                   4.91333  -0.10459   2.77488
 H                   6.70803  -1.22525   1.48092
```

*2,4-Diphenyl-1H-benzo[h]isophosphinoline 2-sulfide (**6**)*

```
Total energy =                                 -1703.96571434 a.u.
Number of imaginary frequencies =              0
Zero-point correction=                         0.362189 (Hartree/Particle)
Thermal correction to Energy=                  0.384112
Thermal correction to Enthalpy=                0.385056
Thermal correction to Gibbs Free Energy=       0.309113
Sum of electronic and zero-point Energies=          -1703.603525
Sum of electronic and thermal Energies=             -1703.581603
Sum of electronic and thermal Enthalpies=           -1703.580659
Sum of electronic and thermal Free Energies=        -1703.656602

Charge =  0 Multiplicity = 1
 C                    3.77603  -0.20426   0.16645
 C                    3.50562  -1.56466  -0.11507
 C                    2.14587  -1.97916  -0.29743
 C                    1.09317  -1.01272  -0.18467
 C                    1.40048   0.32924   0.05616
 C                    2.76093   0.70832   0.23999
 C                   -0.34271  -1.44562  -0.37473
 C                   -0.93155   1.11458   0.49649
 C                    0.35753   1.38186   0.19523
 C                    4.55305  -2.51545  -0.22049
 C                    4.28829  -3.8317   -0.50678
 C                    2.953    -4.24925  -0.69992
 C                    1.91605  -3.35288  -0.5958
 C                    0.75247   2.81407   0.03128
 C                    0.46153   3.75153   1.03036
 C                    0.78325   5.09666   0.85805
 C                    1.39431   5.52617  -0.31798
 C                    1.68918   4.6007   -1.31927
 C                    1.37819   3.25538  -1.14361
 H                    4.80316   0.10731   0.32343
 H                    2.9899    1.74212   0.46041
 H                   -0.48256  -2.50819  -0.18893
 H                   -1.64345   1.91959   0.63918
 H                    5.57364  -2.17893  -0.07104
 H                    5.09653  -4.54984  -0.58495
 H                    2.74277  -5.28794  -0.92858
 H                    0.90882  -3.71376  -0.75135
 H                   -0.00415   3.41869   1.95103
 H                    0.55915   5.80721   1.64579
 H                    1.64249   6.57279  -0.45314
 H                    2.1613    4.92731  -2.23916
 H                    1.6128    2.54163  -1.92547
 H                   -0.67093  -1.24367  -1.40181
 P                   -1.51412  -0.5683    0.74431
 C                   -3.13447  -0.6504   -0.12456
 C                   -4.20358  -1.32035   0.47437
 C                   -3.30657  -0.05935  -1.38343
 C                   -5.43009  -1.40737  -0.18284
 H                   -4.06566  -1.76158   1.4551
 C                   -4.53392  -0.14601  -2.03556
 H                   -2.49084   0.48053  -1.85206
 C                   -5.59654  -0.82277  -1.43645
 H                   -6.25565  -1.92903   0.28809
 H                   -4.66174   0.31762  -3.00731
 H                   -6.55207  -0.88889  -1.94466
 S                   -1.55805  -1.20924   2.61013
```

*2,4-Diphenyl-1H-benzo[h]isophosphinoline 2-selenide (**7**)*

```
Total energy =                                   -3707.30161460 a.u.
Number of imaginary frequencies =                0
Zero-point correction=                           0.361707 (Hartree/Particle)
Thermal correction to Energy=                    0.383876
Thermal correction to Enthalpy=                  0.384820
Thermal correction to Gibbs Free Energy=         0.308071
Sum of electronic and zero-point Energies=            -3706.939908
Sum of electronic and thermal Energies=               -3706.917739
Sum of electronic and thermal Enthalpies=             -3706.916795
Sum of electronic and thermal Free Energies=          -3706.993543

Charge =  0 Multiplicity = 1
 C                    3.87294  -0.48744   0.12601
 C                    3.4875   -1.79551  -0.25255
 C                    2.10665  -2.05965  -0.5317
 C                    1.15012  -0.99821  -0.41877
 C                    1.57225   0.28981  -0.07757
 C                    2.94974   0.51784   0.20249
 C                   -0.30691  -1.26848  -0.71793
 C                   -0.69344   1.27646   0.29589
 C                    0.63046   1.43135   0.07569
 C                    4.44102  -2.8403   -0.35893
 C                    4.06577  -4.10562  -0.73665
 C                    2.70948  -4.37503  -1.02393
 C                    1.7611   -3.3852   -0.92223
 C                    1.16966   2.82416   0.0265
 C                    0.91059   3.72517   1.06719
 C                    1.36967   5.03941   0.99997
 C                    2.08885   5.47481  -0.11098
 C                    2.3532    4.5855   -1.15275
 C                    1.90424   3.2699   -1.08191
 H                    4.9146   -0.29106   0.35634
 H                    3.26535   1.50865   0.49982
 H                   -0.5624   -2.32096  -0.62217
 H                   -1.33107   2.13767   0.45942
 H                    5.47912  -2.61733  -0.13575
 H                    4.80286  -4.89663  -0.8145
 H                    2.41199  -5.37356  -1.32353
 H                    0.73417  -3.63417  -1.15088
 H                    0.36135   3.38626   1.93826
 H                    1.16806   5.72077   1.81895
 H                    2.44411   6.49766  -0.16433
 H                    2.90901   4.91735  -2.02264
 H                    2.11568   2.58399  -1.89468
 H                   -0.5508   -0.95918  -1.74186
 P                   -1.45223  -0.3499    0.39888
 C                   -3.02062  -0.21243  -0.55656
 C                   -4.18455  -0.81145  -0.07052
 C                   -3.05444   0.4789   -1.77535
 C                   -5.36925  -0.72993  -0.80097
 H                   -4.15206  -1.32945   0.88173
 C                   -4.24074   0.56076  -2.50002
 H                   -2.16268   0.96495  -2.15581
 C                   -5.39913  -0.04606  -2.01436
 H                   -6.26911  -1.19749  -0.4175
 H                   -4.26151   1.10128  -3.43968
 H                   -6.32241   0.01941  -2.57911
 Se                  -1.67223  -1.15065   2.36424
```

*2-Methyl-2,4-diphenyl-1,2-dihydrobenzo[h]isophosphinolin-2-ium iodide (**8**)*

```
Total energy =                                -3919.80433798 a.u.
Number of imaginary frequencies =             0
Zero-point correction=                        0.398172 (Hartree/Particle)
Thermal correction to Energy=                 0.422962
Thermal correction to Enthalpy=               0.423906
Thermal correction to Gibbs Free Energy=      0.339655
Sum of electronic and zero-point Energies=         -3919.406166
Sum of electronic and thermal Energies=            -3919.381376
Sum of electronic and thermal Enthalpies=          -3919.380432
Sum of electronic and thermal Free Energies=       -3919.464683

Charge =  0 Multiplicity = 1
 C                    0.14328   3.029    -1.78622
 C                   -0.93973   3.31868  -0.92173
 C                   -1.32442   2.35294   0.06576
 C                   -0.62046   1.10752   0.13922
 C                    0.45641   0.86433  -0.71899
 C                    0.82449   1.84865  -1.67834
 C                   -0.96648   0.11176   1.21634
 C                    0.73238  -1.57989  -0.24474
 C                    1.22006  -0.40957  -0.71316
 C                   -1.63781   4.55053  -1.00849
 C                   -2.67029   4.84122  -0.15196
 C                   -3.04555   3.90386   0.83575
 C                   -2.39324   2.69884   0.9409
 C                    2.61107  -0.40533  -1.25383
 C                    2.96118  -1.16628  -2.3739
 C                    4.27653  -1.17224  -2.83657
 C                    5.25224  -0.43161  -2.17299
 C                    4.90963   0.31585  -1.04578
 C                    3.59531   0.33816  -0.58868
 H                    0.43415   3.76265  -2.53013
 H                    1.65835   1.64698  -2.33746
 H                   -1.96148   0.24725   1.63417
 H                    1.33653  -2.47843  -0.25215
 H                   -1.33206   5.26574  -1.76473
 H                   -3.19365   5.78765  -0.22534
 H                   -3.85132   4.13955   1.5215
 H                   -2.69955   2.01646   1.72162
 H                    2.20169  -1.74266  -2.89116
 H                    4.5369   -1.75689  -3.71183
 H                    6.27665  -0.44163  -2.52786
 H                    5.6693    0.87606  -0.5128
 H                    3.33293   0.89089   0.30654
 H                   -0.20978   0.18232   2.04152
 P                   -0.79034  -1.60068   0.66471
 C                   -2.18955  -2.14436  -0.37109
 C                   -3.39719  -2.53903   0.22164
 C                   -2.07683  -2.12874  -1.76579
 C                   -4.47509  -2.9144   -0.57448
 H                   -3.50399  -2.55927   1.30042
 C                   -3.15897  -2.50686  -2.55767
 H                   -1.14513  -1.82584  -2.22877
 C                   -4.35657  -2.89911  -1.96411
 H                   -5.40513  -3.22132  -0.11033
 H                   -3.06471  -2.49437  -3.6373
 H                   -5.19711  -3.1937   -2.58189
 I                    1.74937  -0.291     3.28268
 C                   -0.63919  -2.68968   2.10213
```

H	-1.55867	-2.6741	2.68929
H	-0.43393	-3.70846	1.76833
H	0.1899	-2.28921	2.70465

*1-Phenylphenanthrene (**12**)*

```
Total energy =                                    -770.77575362 a.u.
Number of imaginary frequencies =                 0
Zero-point correction=                            0.273953 (Hartree/Particle)
Thermal correction to Energy=                     0.288258
Thermal correction to Enthalpy=                   0.289202
Thermal correction to Gibbs Free Energy=          0.231947
Sum of electronic and zero-point Energies=              -770.501801
Sum of electronic and thermal Energies=                 -770.487496
Sum of electronic and thermal Enthalpies=               -770.486552
Sum of electronic and thermal Free Energies=            -770.543807
```

Charge = 0 Multiplicity = 1

C	1.2418	-1.90606	-0.24089
C	2.48658	-1.2067	-0.1369
C	2.4817	0.20917	0.00673
C	1.20734	0.91664	0.05286
C	-0.01064	0.17431	-0.01596
C	0.0539	-1.25113	-0.18187
C	1.13472	2.32305	0.15335
C	-1.27581	2.24383	0.12712
C	-1.26187	0.86018	0.04331
C	3.71549	-1.90355	-0.18368
C	4.91631	-1.23384	-0.09063
C	4.91944	0.16483	0.0526
C	3.73059	0.86577	0.09876
C	-2.56528	0.13287	0.02374
C	-3.48684	0.35831	-1.00702
C	-4.72487	-0.28241	-1.01131
C	-5.06471	-1.15689	0.01886
C	-4.15808	-1.38635	1.05321
C	-2.91966	-0.74892	1.05463
H	1.26479	-2.98274	-0.37432
H	-0.87042	-1.80551	-0.27461
H	2.04129	2.91107	0.20414
H	-2.22679	2.76227	0.17539
H	3.69688	-2.98284	-0.29467
H	5.85261	-1.77907	-0.12696
H	5.86043	0.69814	0.12807
H	3.77072	1.94109	0.21155
H	-3.224	1.03252	-1.81473
H	-5.42209	-0.09912	-1.82147
H	-6.028	-1.6544	0.01723
H	-4.41731	-2.05832	1.86398
H	-2.22433	-0.92321	1.86835
C	-0.08017	2.97433	0.1804
H	-0.11342	4.05551	0.25421

*4-Phenylbenzo[h]isoquinoline (**13**)*

```
Total energy =                                 -786.81722065 a.u.
Number of imaginary frequencies =              0
Zero-point correction=                         0.262305 (Hartree/Particle)
Thermal correction to Energy=                  0.276492
Thermal correction to Enthalpy=                0.277436
Thermal correction to Gibbs Free Energy=       0.220330
Sum of electronic and zero-point Energies=           -786.554916
Sum of electronic and thermal Energies=              -786.540728
Sum of electronic and thermal Enthalpies=            -786.539784
Sum of electronic and thermal Free Energies=         -786.596891

Charge =  0 Multiplicity = 1
 C                    1.28011  -1.91645  -0.24519
 C                    2.51252  -1.19287  -0.14097
 C                    2.48104   0.22187   0.00952
 C                    1.19022   0.89165   0.06048
 C                   -0.00948   0.13059  -0.01187
 C                    0.07546  -1.29208  -0.18258
 C                    1.0615    2.29441   0.16457
 C                   -1.21898   2.20641   0.15058
 C                   -1.25152   0.82366   0.05349
 C                    3.75436  -1.86494  -0.19402
 C                    4.94071  -1.16951  -0.10019
 C                    4.91579   0.22816   0.05012
 C                    3.71406   0.90657   0.10294
 C                   -2.56903   0.12986   0.02553
 C                   -3.49919   0.41869  -0.98175
 C                   -4.75065  -0.19486  -0.99384
 C                   -5.09438  -1.105     0.00354
 C                   -4.1792   -1.39723   1.0143
 C                   -2.92787  -0.78617   1.02496
 H                    1.32756  -2.99197  -0.38236
 H                   -0.83851  -1.86357  -0.27464
 H                    1.94528   2.92146   0.21348
 H                   -2.14925   2.76362   0.20677
 H                    3.75862  -2.94374  -0.31015
 H                    5.88816  -1.69452  -0.14127
 H                    5.84634   0.7792    0.12618
 H                    3.73075   1.98181   0.22147
 H                   -3.23315   1.12016  -1.76466
 H                   -5.4554    0.03769  -1.78443
 H                   -6.06825  -1.58128  -0.00455
 H                   -4.4424   -2.09646   1.80024
 H                   -2.22759  -1.00589   1.82327
 N                   -0.09072   2.93955   0.20186
```

Frontier MOs of compounds 1, 2, 3, 4, 12, and 13

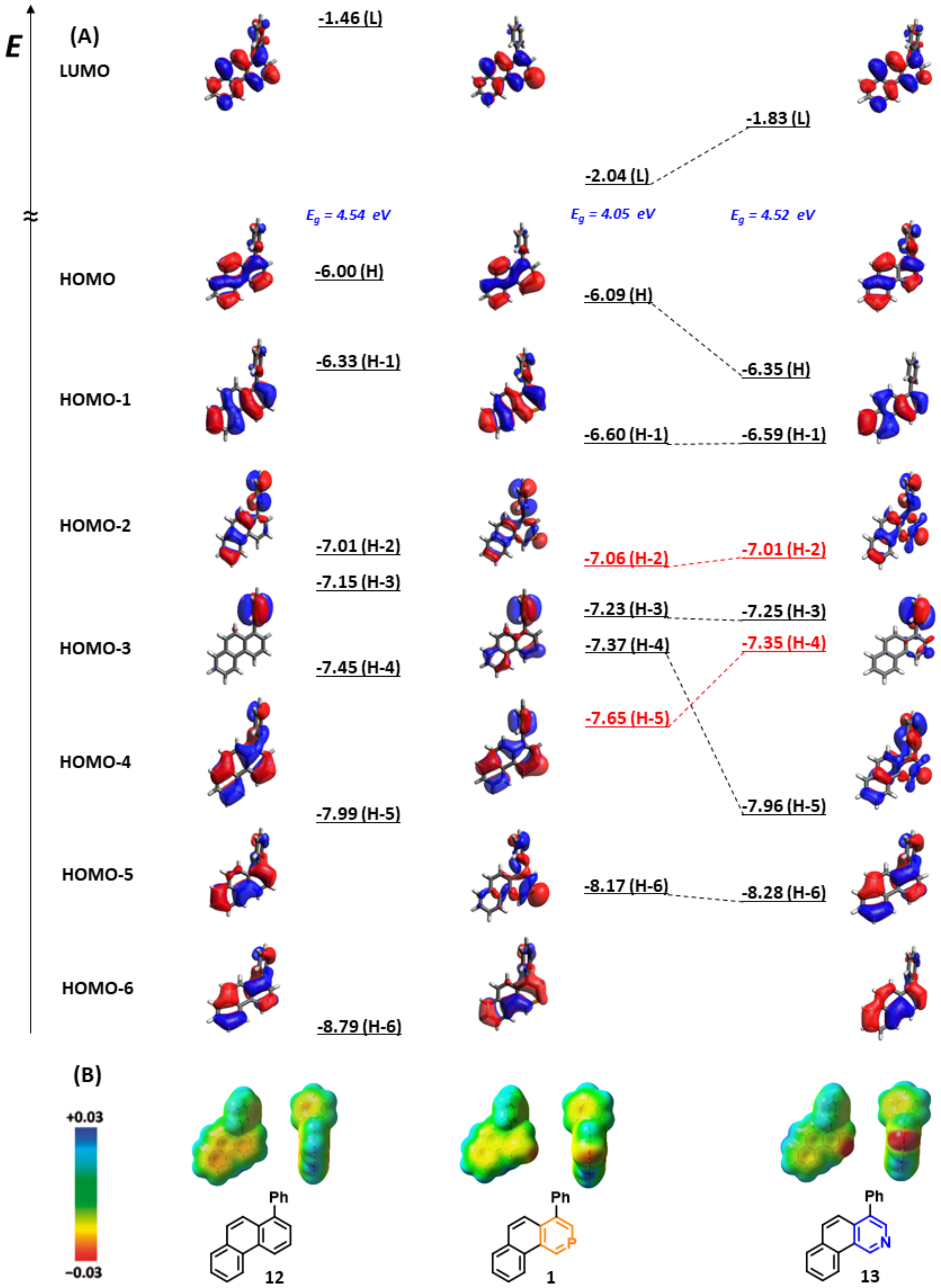

Figure S16. (A) Calculated HOMO/LUMO orbitals and energy gaps (E_g) (DFT, B3LYP/6-311G++(d,p)) for 1-phenyl phenanthrene **12** and its derivatives containing phosphorus **1** and nitrogen **13** atoms. (B) Electrostatic potentials mapped onto the electron density isosurface for series compounds **1**, **12**, and **13** in the range from – 0.03 (red) to 0.03 (blue).

The vertical energy electronic transitions, corresponding wavelengths with oscillator strength (f), and their major orbital contributions for compounds 1, 2, 3, 4, 5, 6, 7, 8, 12, and 13

Table S1 Calculated vertical energy electronic transition, wavelength, oscillator strength, and major orbital contributions of **1** by the B3LYP/6-311++G(d,p) method

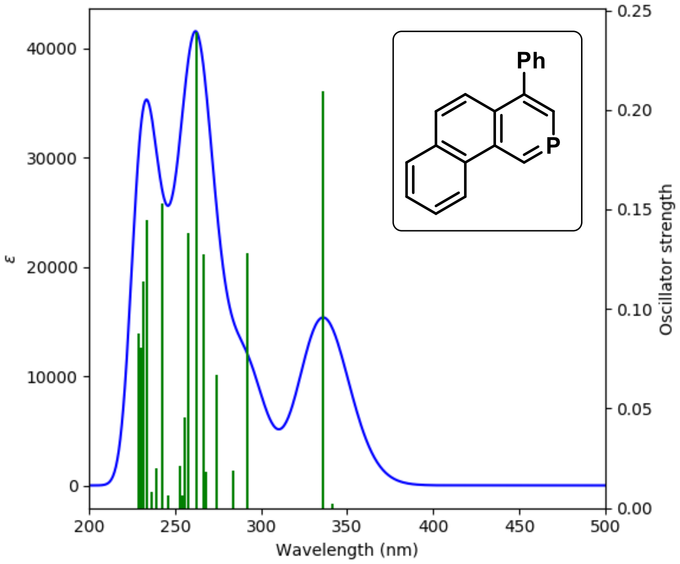

Energy (eV)	Wavelength (nm)	Osc. Strength	Major orbital contributions
3.53	228.77	0.0874	H-4->L+1 (14%), HOMO->L+6 (38%), HOMO->L+7 (14%)
3.51	229.82	0.0807	H-6->LUMO (31%), H-1->L+3 (46%)
3.48	231.57	0.1136	H-6->LUMO (10%), H-1->L+2 (16%), H-1->L+3 (19%), H-1->L+4 (14%)
**3.46**	**233.34**	**0.1445**	H-1->L+2 (19%), H-1->L+3 (14%), H-1->L+4 (26%)
3.41	236.48	0.0083	HOMO->L+5 (86%)
3.38	238.73	0.0201	H-5->L+1 (79%)
**3.32**	**242.75**	**0.1531**	H-4->LUMO (41%), HOMO->L+2 (20%)
3.28	245.61	0.0061	H-4->L+1 (11%), H-3->L+1 (72%)
3.19	252.55	0.021	HOMO->L+4 (81%)
3.17	254.15	0.0062	H-4->L+1 (18%), H-3->L+1 (15%), H-1->L+2 (16%), HOMO->L+3 (26%)
3.16	255.24	0.0456	H-5->LUMO (68%)
3.13	257.32	0.1381	H-5->LUMO (10%), H-2->L+1 (16%), H-1->L+1 (11%), HOMO->L+3 (41%)
**3.07**	**262.68**	**0.2389**	H-4->LUMO (17%), H-2->L+1 (26%), H-1->L+1 (10%), H-1->L+2 (16%)
3.03	266.31	0.1276	H-3->LUMO (43%), H-2->L+1 (16%), H-1->L+1 (24%)
3.01	268.06	0.0182	H-4->LUMO (16%), H-3->LUMO (33%), H-2->L+1 (26%), HOMO->L+2 (11%)
2.94	274.37	0.067	H-4->LUMO (16%), H-3->LUMO (15%), H-1->L+1 (29%), HOMO->L+2 (33%)
2.84	284.04	0.0188	H-2->LUMO (74%)
**2.76**	**292.12**	**0.128**	H-2->LUMO (17%), H-1->LUMO (46%), HOMO->L+1 (29%)
**2.40**	**336.04**	**0.2095**	**HOMO->LUMO (88%)**
2.36	341.25	0.0025	H-1->LUMO (39%), HOMO->L+1 (58%)

Table S2 Calculated vertical energy electronic transition, wavelength, oscillator strength, and major orbital contributions of **2** by the B3LYP/6-311++G(d,p) method

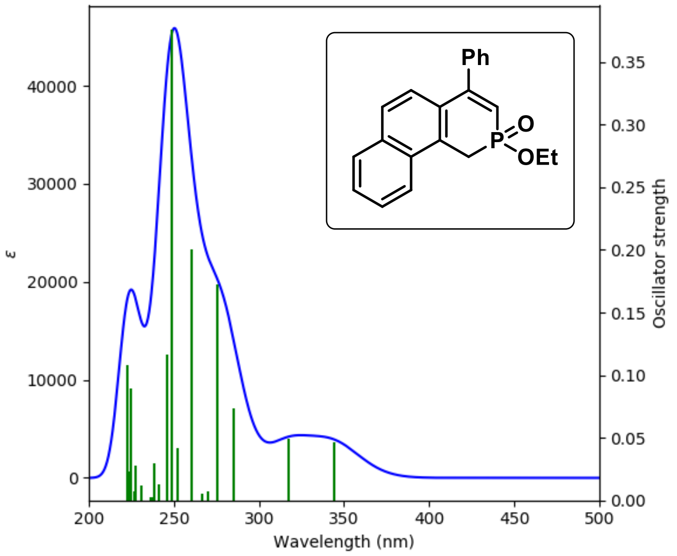

Energy (eV)	Wavelength (nm)	Osc. Strength	Major orbital contributions
3.62	222.80	0.11	H-4->L+1 (51%)
3.60	223.83	0.02	H-3->L+1 (34%), H-1->L+3 (20%), HOMO->L+7 (14%)
**3.59**	**224.60**	**0.09**	H-3->L+1 (14%), HOMO->L+5 (12%), HOMO->L+7 (15%), HOMO->L+8 (14%)
3.56	226.58	0.01	H-7->LUMO (10%), HOMO->L+4 (10%), HOMO->L+5 (39%), HOMO->L+6 (28%)
3.55	227.44	0.03	H-7->LUMO (59%), HOMO->L+6 (17%)
3.50	230.53	0.01	H-6->LUMO (10%), H-2->L+1 (55%)
3.42	236.07	0.00	H-2->L+1 (11%), H-1->L+3 (40%)
3.41	236.85	0.00	HOMO->L+4 (64%), HOMO->L+6 (23%)
3.39	238.17	0.03	H-6->LUMO (47%), H-1->L+2 (16%), HOMO->L+4 (12%)
3.35	240.90	0.01	H-6->LUMO (10%), H-1->L+2 (55%)
3.28	246.13	0.12	H-5->LUMO (63%)
**3.24**	**248.97**	**0.38**	H-5->LUMO (24%), H-1->L+1 (45%)
3.20	252.25	0.04	HOMO->L+3 (86%)
**3.10**	**260.02**	**0.20**	H-3->LUMO (32%), H-1->L+1 (19%), HOMO->L+2 (25%)
3.03	266.59	0.01	H-4->LUMO (66%), H-3->LUMO (19%)
2.99	269.92	0.01	H-4->LUMO (18%), H-3->LUMO (33%), HOMO->L+2 (37%)
**2.93**	**275.63**	**0.17**	H-3->LUMO (10%), H-2->LUMO (11%), H-1->LUMO (10%), HOMO->L+1 (47%)
2.83	285.33	0.07	H-2->LUMO (68%), H-1->LUMO (12%), HOMO->L+1 (10%)
2.54	317.22	0.05	H-1->LUMO (66%), HOMO->L+1 (23%)
**2.34**	**344.41**	**0.05**	**HOMO->LUMO (93%)**

Table S3 Calculated vertical energy electronic transition, wavelength, oscillator strength, and major orbital contributions of **3** by the B3LYP/6-311++G(d,p) method

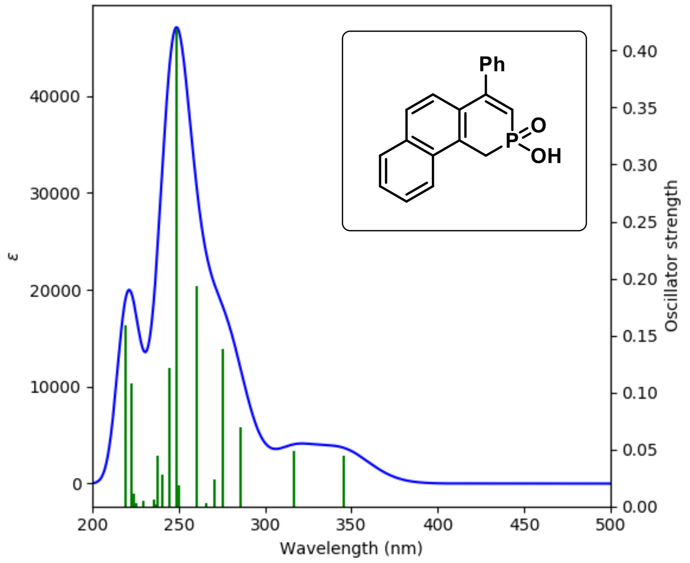

Energy (eV)	Wavelength (nm)	Osc. Strength	Major orbital contributions
3.68	219.27	0.16	H-4->L+1 (47%)
3.62	222.86	0.11	H-7->LUMO (23%), H-4->L+1 (17%)
3.60	223.77	0.01	H-3->L+1 (34%), H-1->L+3 (18%), HOMO->L+6 (14%), HOMO->L+7 (13%)
3.60	224.11	0.00	H-3->L+1 (21%), HOMO->L+5 (20%), HOMO->L+6 (19%), HOMO->L+7 (12%)
3.58	225.03	0.00	HOMO->L+5 (23%), HOMO->L+7 (61%)
3.51	229.77	0.00	H-6->LUMO (11%), H-2->L+1 (48%), H-1->L+2 (12%)
3.43	235.43	0.01	H-2->L+1 (14%), H-1->L+3 (30%), HOMO->L+4 (16%)
3.42	236.01	0.00	HOMO->L+4 (71%)
3.40	237.38	0.04	H-6->LUMO (58%), H-1->L+2 (16%), HOMO->L+6 (13%)
3.36	240.10	0.03	H-1->L+2 (55%)
3.30	244.52	0.12	H-5->LUMO (65%)
**3.25**	**248.43**	**0.42**	H-5->LUMO (22%), H-1->L+1 (42%), HOMO->L+2 (11%)
3.22	250.32	0.02	HOMO->L+3 (85%)
**3.10**	**260.19**	**0.19**	H-3->LUMO (23%), H-1->L+1 (25%), HOMO->L+2 (29%)
3.04	265.59	0.00	H-4->LUMO (88%)
2.98	270.50	0.02	H-3->LUMO (47%), HOMO->L+1 (12%), HOMO->L+2 (31%)
**2.93**	**275.37**	**0.14**	H-3->LUMO (21%), H-2->LUMO (14%), HOMO->L+1 (41%)
2.82	285.83	0.07	H-2->LUMO (67%), H-1->LUMO (12%), HOMO->L+1 (11%)
2.54	317.01	0.05	H-1->LUMO (67%), HOMO->L+1 (21%)
**2.33**	**345.65**	**0.04**	**HOMO->LUMO (93%)**

Table S4 Calculated vertical energy electronic transition, wavelength, oscillator strength, and major orbital contributions of **4** by the B3LYP/6-311++G(d,p) method

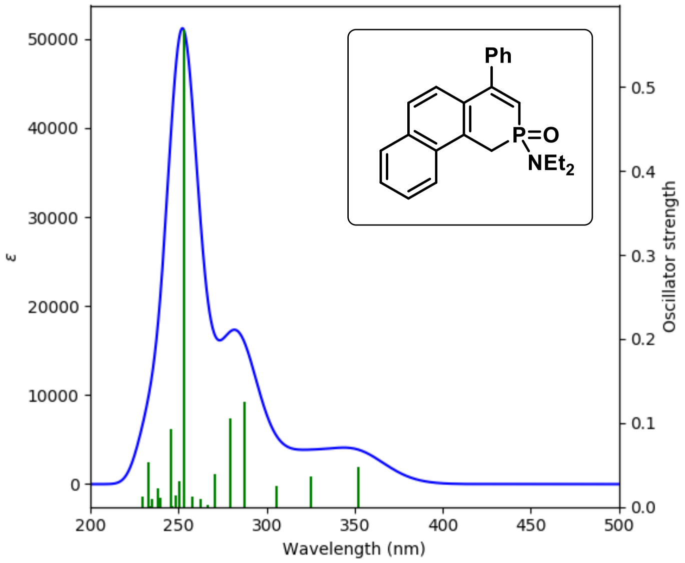

Energy (eV)	Wavelength (nm)	Osc. Strength	Major orbital contributions
3.51	229.83	0.01	H-7->LUMO (13%), H-3->L+1 (38%)
3.46	232.85	0.05	H-2->L+2 (75%)
3.44	234.36	0.00	HOMO->L+5 (53%), HOMO->L+6 (26%)
3.43	235.01	0.01	H-1->L+4 (12%), HOMO->L+5 (34%), HOMO->L+6 (20%), HOMO->L+7 (18%)
3.38	238.38	0.02	H-7->LUMO (10%), H-3->L+1 (19%), H-1->L+3 (34%)
3.37	239.61	0.01	H-7->LUMO (23%), H-1->L+3 (11%), HOMO->L+6 (27%), HOMO->L+7 (19%)
3.28	245.81	0.09	H-2->L+1 (17%), H-1->L+2 (45%)
3.27	246.47	0.00	H-6->LUMO (83%)
3.25	248.07	0.01	HOMO->L+4 (84%)
3.22	250.41	0.03	H-2->L+1 (66%), H-1->L+2 (20%)
**3.19**	**253.09**	**0.57**	H-3->LUMO (11%), H-1->L+1 (24%), HOMO->L+2 (14%), HOMO->L+3 (15%)
3.12	258.12	0.01	H-1->L+1 (11%), HOMO->L+3 (69%)
3.08	262.26	0.01	H-5->LUMO (40%), H-4->LUMO (31%), H-1->L+1 (15%)
3.02	266.70	0.00	H-5->LUMO (48%), H-4->LUMO (28%), H-1->L+1 (19%)
2.98	270.44	0.04	H-4->LUMO (16%), H-3->LUMO (13%), H-1->L+1 (20%), HOMO->L+2 (38%)
2.89	279.39	0.11	H-3->LUMO (20%), HOMO->L+1 (35%), HOMO->L+2 (31%)
**2.81**	**287.29**	**0.12**	H-3->LUMO (45%), H-2->LUMO (19%), HOMO->L+1 (21%)
2.64	305.58	0.02	H-2->LUMO (62%), H-1->LUMO (11%), HOMO->L+1 (18%)
2.48	325.01	0.04	H-1->LUMO (77%), HOMO->L+1 (16%)
**2.29**	**351.81**	**0.05**	**HOMO->LUMO (94%)**

Table S5 Calculated vertical energy electronic transition, wavelength, oscillator strength, and major orbital contributions of **5** by the B3LYP/6-311++G(d,p) method

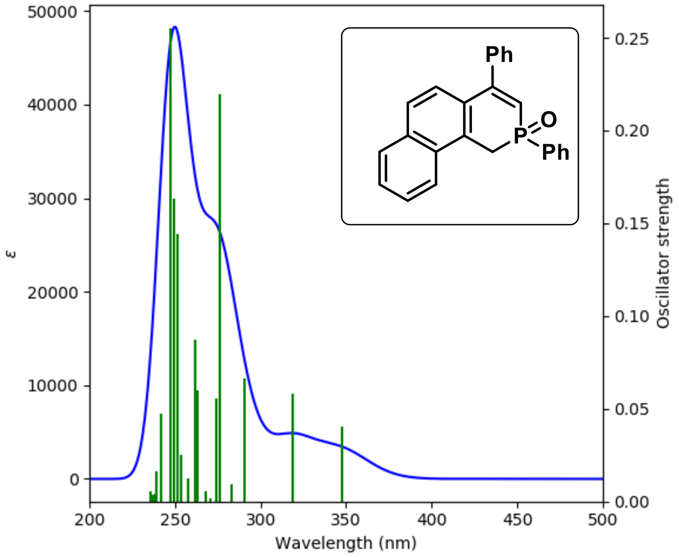

Energy (eV)	Wavelength (nm)	Osc. Strength	Major orbital contributions
3.42	235.93	0.01	H-6->L+4 (14%), H-5->L+2 (12%), H-3->L+2 (29%)
3.40	237.07	0.00	H-2->L+1 (25%), H-1->L+5 (34%)
3.38	238.41	0.00	H-8->LUMO (26%), HOMO->L+6 (46%), HOMO->L+8 (10%)
3.37	239.29	0.02	H-8->LUMO (34%), HOMO->L+6 (35%), HOMO->L+9 (11%)
3.34	241.71	0.05	H-1->L+3 (64%)
**3.26**	**247.11**	**0.26**	H-1->L+2 (57%)
3.23	249.64	0.16	H-1->L+1 (16%), H-1->L+2 (32%), HOMO->L+4 (22%)
3.21	251.40	0.14	H-1->L+1 (18%), HOMO->L+4 (60%)
3.18	253.36	0.02	H-1->L+1 (11%), HOMO->L+5 (70%)
3.13	257.65	0.01	H-7->LUMO (56%), H-6->LUMO (26%)
3.08	261.54	0.09	H-5->LUMO (24%), H-4->LUMO (43%), H-1->L+1 (11%)
3.07	263.04	0.06	H-7->LUMO (13%), H-5->LUMO (49%), HOMO->L+3 (13%)
3.01	267.61	0.01	H-7->LUMO (20%), H-6->LUMO (28%), H-5->LUMO (13%), H-4->LUMO (26%)
2.98	270.42	0.00	H-6->LUMO (26%), H-4->LUMO (19%), HOMO->L+3 (34%)
2.94	273.90	0.06	HOMO->L+2 (90%)
**2.92**	**276.23**	**0.22**	H-2->LUMO (10%), HOMO->L+1 (48%), HOMO->L+3 (10%)
2.85	282.93	0.01	H-3->LUMO (76%)
2.77	290.87	0.07	H-2->LUMO (72%), HOMO->L+1 (10%)
2.53	318.68	0.06	H-1->LUMO (70%), HOMO->L+1 (20%)
**2.32**	**347.82**	**0.04**	**HOMO->LUMO (93%)**

Table S6 Calculated vertical energy electronic transition, wavelength, oscillator strength, and major orbital contributions of **6** by the B3LYP/6-311++G(d,p) method

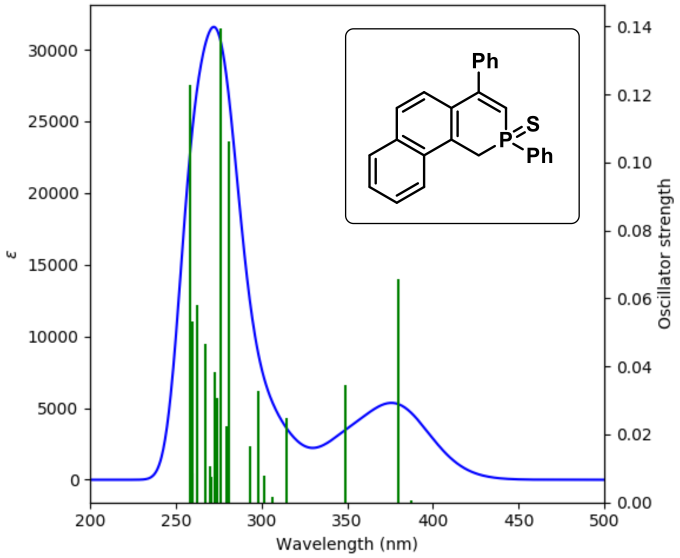

Energy (eV)	Wavelength (nm)	Osc. Strength	Major orbital contributions
**3.13**	**257.95**	**0.12**	H-7->LUMO (40%), H-6->LUMO (13%), H-2->L+3 (16%)
3.11	259.41	0.05	H-6->LUMO (68%), HOMO->L+5 (23%)
3.10	259.85	0.01	H-6->LUMO (16%), H-5->LUMO (11%), HOMO->L+5 (58%)
3.07	262.62	0.06	H-7->LUMO (44%), H-2->L+3 (34%)
3.02	266.82	0.05	H-5->LUMO (24%), H-2->L+3 (12%), H-1->L+3 (47%)
2.99	269.81	0.01	H-1->L+4 (61%), HOMO->L+4 (12%)
2.98	270.23	0.01	H-5->LUMO (27%), H-1->L+3 (33%), H-1->L+4 (18%)
2.96	272.80	0.04	H-5->LUMO (10%), HOMO->L+3 (37%), HOMO->L+4 (31%)
2.95	273.72	0.03	HOMO->L+3 (24%), HOMO->L+4 (53%)
**2.92**	**276.36**	**0.14**	H-4->LUMO (15%), H-2->L+1 (33%), HOMO->L+3 (27%)
2.88	279.70	0.02	H-2->L+2 (85%)
2.87	280.77	0.11	H-4->LUMO (50%), H-3->LUMO (16%), H-2->L+1 (18%)
2.75	293.04	0.02	H-2->L+1 (12%), H-1->L+1 (72%)
2.71	297.85	0.03	HOMO->L+1 (66%), HOMO->L+2 (20%)
2.67	301.72	0.01	H-1->L+1 (12%), H-1->L+2 (61%), HOMO->L+1 (14%)
2.63	306.59	0.00	H-1->L+2 (13%), HOMO->L+2 (70%)
2.56	314.66	0.02	H-3->LUMO (63%), H-2->L+1 (13%)
2.31	348.82	0.03	H-2->LUMO (86%)
2.12	379.96	0.07	HOMO->LUMO (94%)
**2.08**	**387.58**	**0.00**	**H-1->LUMO (90%)**

Table S7 Calculated vertical energy electronic transition, wavelength, oscillator strength, and major orbital contributions of **7** by the B3LYP/6-311++G(d,p) method

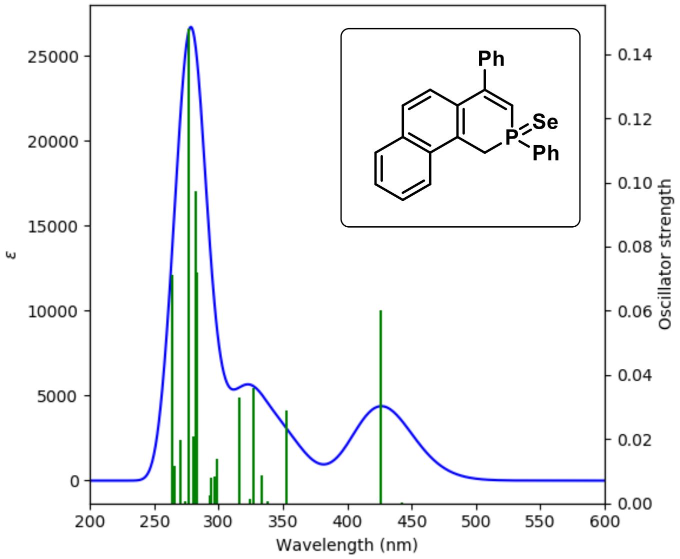

Energy (eV)	Wavelength (nm)	Osc. Strength	Major orbital contributions
3.05	264.02	0.07	H-7->LUMO (28%), H-5->LUMO (19%), H-2->L+3 (25%)
3.03	265.96	0.01	HOMO->L+6 (70%)
2.98	270.37	0.02	H-5->LUMO (62%), H-2->L+3 (17%)
2.94	274.20	0.00	H-1->L+5 (97%)
**2.92**	**276.27**	**0.15**	H-4->LUMO (23%), H-2->L+1 (35%), H-2->L+3 (17%), HOMO->L+5 (16%)
2.88	280.15	0.02	HOMO->L+5 (77%)
2.86	282.41	0.10	H-4->LUMO (20%), H-2->L+1 (11%), H-2->L+2 (57%)
2.85	283.15	0.07	H-4->LUMO (28%), H-3->LUMO (10%), H-2->L+1 (13%), H-2->L+2 (39%)
2.75	292.75	0.00	H-1->L+3 (84%), H-1->L+4 (12%)
2.74	294.46	0.01	H-1->L+4 (41%), HOMO->L+4 (42%)
2.72	296.56	0.01	H-1->L+4 (33%), HOMO->L+3 (48%), HOMO->L+4 (14%)
2.70	298.51	0.01	H-1->L+4 (12%), HOMO->L+3 (43%), HOMO->L+4 (42%)
2.55	316.08	0.03	H-3->LUMO (66%), H-2->L+1 (22%)
2.48	324.65	0.00	H-1->L+1 (84%), H-1->L+2 (11%)
2.46	327.20	0.04	HOMO->L+1 (72%), HOMO->L+2 (21%)
2.42	333.18	0.01	H-1->L+2 (59%), HOMO->L+1 (12%), HOMO->L+2 (20%)
2.38	338.27	0.00	H-1->L+2 (28%), HOMO->L+1 (11%), HOMO->L+2 (57%)
2.29	352.92	0.03	H-2->LUMO (94%)
**1.89**	**426.40**	**0.06**	**HOMO->LUMO (98%)**
1.82	442.09	0.00	H-1->LUMO (99%)

Table S8 Calculated vertical energy electronic transition, wavelength, oscillator strength, and major orbital contributions of **8** by the B3LYP/6-311++G(d,p) method

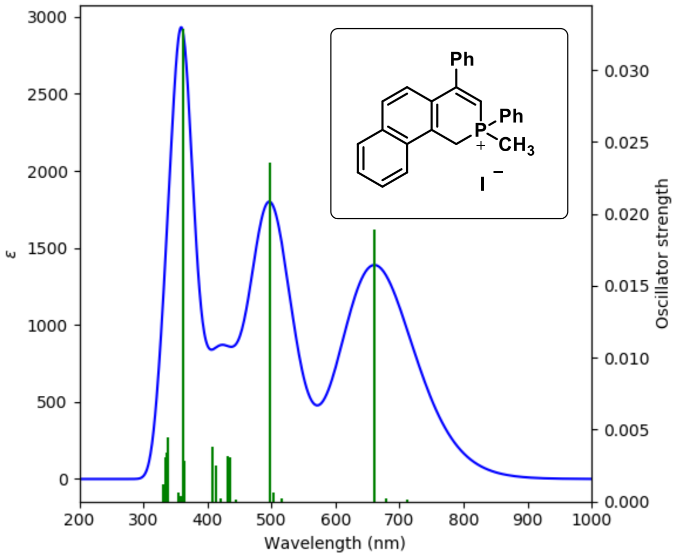

Energy (eV)	Wavelength (nm)	Osc. Strength	Major orbital contributions
2.43	331.25	0.001	H-1->L+6 (88%)
2.42	333.87	0.003	H-1->L+5 (92%)
2.39	337.25	0.003	HOMO->L+6 (97%)
2.38	338.53	0.005	HOMO->L+5 (93%)
2.27	355.17	0.001	H-2->L+4 (95%)
2.25	358.21	0.000	H-1->L+4 (94%)
**2.23**	**361.62**	**0.033**	H-3->LUMO (94%)
2.21	364.11	0.003	HOMO->L+4 (98%)
1.97	408.78	0.004	H-2->L+3 (89%)
1.95	412.70	0.003	H-1->L+3 (86%)
1.92	419.87	0.000	HOMO->L+3 (90%)
1.87	431.49	0.003	H-2->L+2 (87%)
1.85	435.72	0.003	H-1->L+2 (83%)
1.82	443.70	0.000	HOMO->L+2 (91%)
**1.62**	**497.93**	**0.024**	H-1->L+1 (95%)
1.61	502.41	0.001	H-2->L+1 (94%)
1.56	516.00	0.000	HOMO->L+1 (98%)
**1.22**	**660.69**	**0.019**	H-2->LUMO (39%), H-1->LUMO (49%), HOMO->LUMO (11%)
1.19	680.00	0.000	H-2->LUMO (56%), H-1->LUMO (44%)
**1.13**	**712.63**	**0.000**	**HOMO->LUMO (88%)**

Table S9 Calculated vertical energy electronic transition, wavelength, oscillator strength, and major orbital contributions of **12** by the B3LYP/6-311++G(d,p) method

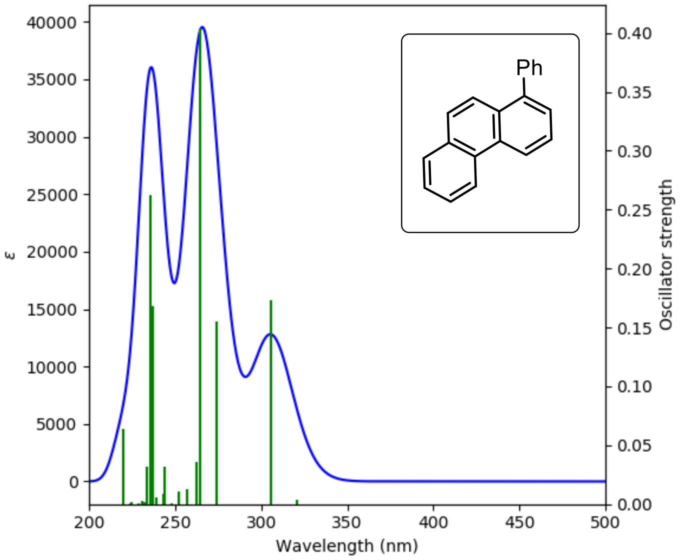

Energy (eV)	Wavelength (nm)	Osc. Strength	Major orbital contributions
3.66	220.07	0.063	H-4->LUMO (43%), HOMO->L+5 (10%)
3.60	223.73	0.001	H-3->L+2 (29%), H-2->L+3 (29%)
3.59	224.36	0.002	H-1->L+4 (36%), H-1->L+5 (25%), H-1->L+6 (18%), HOMO->L+7 (10%)
3.53	228.77	0.001	H-1->L+4 (10%), HOMO->L+7 (76%)
3.50	230.45	0.003	HOMO->L+6 (79%)
3.47	232.29	0.002	H-4->L+1 (20%), H-3->L+1 (29%), H-1->L+4 (11%), H-1->L+5 (15%)
3.46	233.41	0.032	H-3->L+1 (52%)
**3.43**	**235.34**	**0.262**	H-4->L+1 (10%), H-2->L+1 (30%), H-1->L+3 (43%)
3.41	236.68	0.168	H-3->LUMO (13%), H-1->L+2 (23%), H-1->L+3 (32%)
3.37	238.99	0.006	HOMO->L+4 (52%), HOMO->L+5 (37%)
3.32	242.76	0.008	H-4->LUMO (22%), H-3->LUMO (23%), HOMO->L+5 (19%)
3.31	244.01	0.032	H-3->LUMO (47%)
3.26	247.63	0.001	H-2->LUMO (42%), H-2->L+1 (14%)
3.20	251.92	0.010	H-2->L+1 (28%), H-1->L+2 (36%)
3.14	256.82	0.013	HOMO->L+3 (69%)
3.07	262.33	0.035	H-2->LUMO (20%), HOMO->L+2 (56%)
**3.05**	**264.26**	**0.404**	H-1->LUMO (13%), H-1->L+1 (54%)
2.94	274.25	0.156	H-1->LUMO (40%), H-1->L+1 (12%), HOMO->L+1 (25%), HOMO->L+2 (17%)
**2.64**	**305.61**	**0.173**	**H-1->L+1 (14%), HOMO->LUMO (83%)**
2.52	320.59	0.004	H-1->LUMO (35%), HOMO->L+1 (63%)

Table S10 Calculated vertical energy electronic transition, wavelength, oscillator strength, and major orbital contributions of **13** by the B3LYP/6-311++G(d,p) method

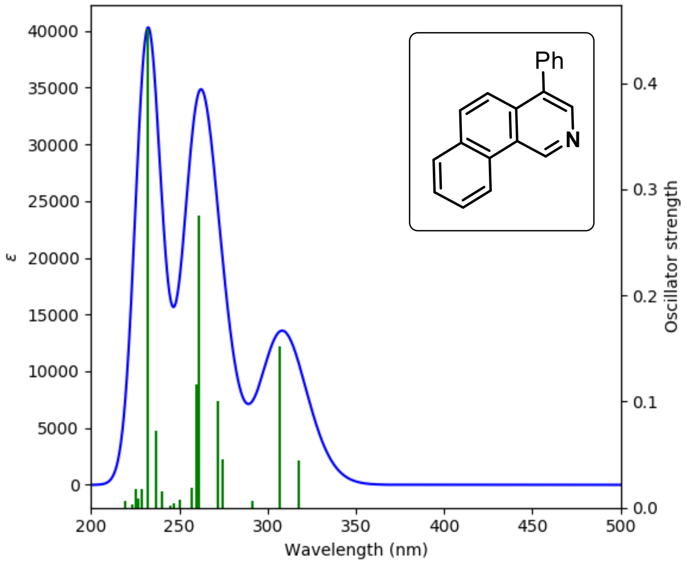

Energy (eV)	Wavelength (nm)	Osc. Strength	Major orbital contributions
3.67	219.60	0.007	H-1->L+5 (29%), HOMO->L+6 (54%)
3.62	223.08	0.003	H-3->L+2 (17%), H-2->L+3 (24%), H-1->L+3 (11%), H-1->L+4 (18%)
3.58	225.59	0.017	H-2->L+2 (67%)
3.57	225.82	0.004	H-1->L+4 (43%), HOMO->L+5 (17%)
3.56	226.75	0.009	HOMO->L+5 (74%)
3.53	228.77	0.018	H-1->L+3 (77%)
**3.48**	**232.06**	**0.452**	H-4->L+1 (32%), H-1->L+2 (27%)
3.40	237.08	0.072	H-3->L+1 (51%), HOMO->L+4 (27%)
3.36	240.04	0.015	H-5->LUMO (10%), H-3->L+1 (23%), HOMO->L+3 (14%), HOMO->L+4 (41%)
3.29	244.80	0.002	H-4->L+1 (28%), HOMO->L+2 (14%), HOMO->L+3 (14%)
3.27	246.88	0.005	H-4->LUMO (17%), HOMO->L+2 (16%), HOMO->L+3 (42%)
3.22	250.55	0.008	H-4->L+1 (14%), H-1->L+2 (32%), HOMO->L+2 (20%)
3.14	256.87	0.019	H-4->LUMO (10%), H-3->LUMO (64%)
3.11	259.66	0.116	H-4->LUMO (17%), H-3->LUMO (15%), H-1->L+1 (19%), HOMO->L+2 (30%)
**3.09**	**261.29**	**0.275**	H-4->LUMO (24%), H-2->L+1 (10%), H-1->L+1 (30%), HOMO->L+1 (12%)
2.97	271.90	0.101	H-2->L+1 (16%), H-1->LUMO (23%), H-1->L+1 (24%), HOMO->L+1 (16%)
2.94	274.74	0.046	H-2->L+1 (48%), H-1->LUMO (12%), HOMO->L+1 (20%)
2.77	291.42	0.006	H-2->LUMO (68%)
**2.63**	**306.73**	**0.152**	**H-1->LUMO (16%), HOMO->LUMO (55%), HOMO->L+1 (19%)**
2.54	317.44	0.044	H-1->LUMO (34%), HOMO->LUMO (30%), HOMO->L+1 (27%)

5. Quantum yields

Table S11: Absolute photoluminescence quantum yield and osc. strength of compounds **1** – **8**, **12** and **13.**

Compound	Φ_{PL} [%]	f
1	2.5	0.2095
2	1.4	0.0466
3	3.8	0.0449
4	1.0	0.0469
5	0.9	0.0405
6	2.6	0.0658
7	1.4	0.0601
8	1.4	0.0001
12	7.9	0.1727
13	20.4	0.1524

6. Comparison of solid-state and solution-phase emission for acid **3**

As shown in Figure S30, the absorption spectrum at 356 nm is indicative of the monomeric state of the compound. This observation was confirmed by solid-state emission spectroscopy, which showed that the molecule exists preferentially in its self-associated form. When 5% of methanol was added, immediate measurement showed the near-total disappearance of these aggregate bands, indicating the rapid disruption of the H-bonded network by the solvent. After a period of elapsed time, a moderate increase in these additional peaks was observed.

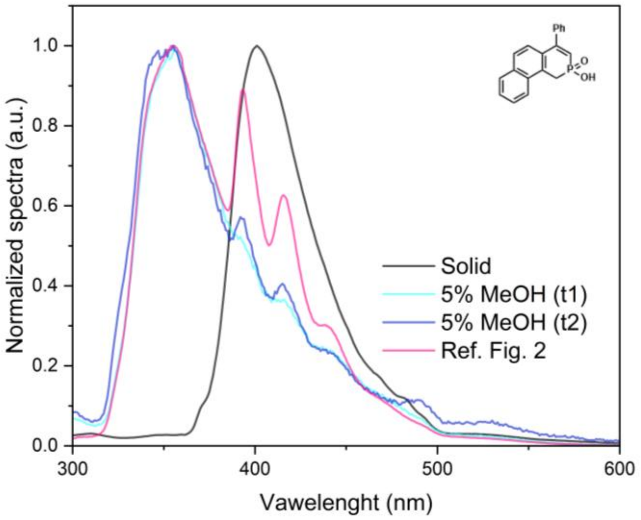

*Figure S30: Comparison of solid-state and time-dependent solution emission spectra of acid **3** in 5% MeOH.*

7. References

(1) Chen, H.; Shen, C.; Dong, K. Parallel Paired Photoelectrochemical Bromination of Alkylarenes with Electrochemical Pinacol Coupling. *J. Org. Chem.* **2024**, *89*(4), 2550–2555. https://doi.org/10.1021/acs.joc.3c02556.

(2) Mizerová, E.; Kos, M.; Jakubec, M.; Pavlica, M.; Žádný, J.; Církva, V.; Storch, J.; Beránek, T. Introduction of Phosphinine Ring into Aromatic Systems via Alkyne Cyclization. *Adv. Synth. Catal.* **2025**, *367*(2), e202401203. https://doi.org/10.1002/adsc.202401203.

(3) Hariri, M.; Darvish, F.; Mengue Me Ndong, K. P.; Sechet, N.; Chacktas, G.; Boosaliki, H.; Tran Do, M. L.; Mwande-Maguene, G.; Lebibi, J.; Burilov, A. R.; Ayad, T.; Virieux, D.; Pirat, J. L. Gold-Catalyzed Access to Isophosphinoline 2-Oxides. *J. Org. Chem.* **2021**, *86*(11), 7813–7824. https://doi.org/10.1021/acs.joc.1c00648.

(4) Huang, H.; Denne, J.; Yang, C.; Wang, H.; Kang, J. Y. Direct Aryloxylation/Alkyloxylation of Dialkyl Phosphonates for the Synthesis of Mixed Phosphonates. *Angew. Chem., Int. Ed.* **2018**, *57*(22), 6624-6628. https://doi.org/10.1002/anie.201802082.

(5) Liu, X.; Sotiropoulos, J. M.; Taillefer, M. A New Route to *E*-Stilbenes through the Transition-Metal-Free KO*t*Bu/DMF-Promoted Direct Coupling of Alcohols with Phenyl Acetonitriles. *Eur. J. Org. Chem.* **2022**, *2022*(26), e202200631. https://doi.org/10.1002/ejoc.202200631.

(6) Wang, X.; Zhong, J.; Luo, M.; Zeng, X. Cr-Catalyzed Intramolecular Arylative Cross-Coupling of Unactivated C-H Bonds with C-Halide Bonds. *Org. Lett.* **2024**, *26*(19), 4093–4097. https://doi.org/10.1021/acs.orglett.4c01145.

(7) Full, F.; Wölflick, Q.; Radacki, K.; Braunschweig, H.; Nowak-Król, A. Enhanced Optical Properties of Azaborole Helicenes by Lateral and Helical Extension. *Chem. - Eur. J.* **2022**, *28*(62), e202202280. https://doi.org/10.1002/chem.202202280.

(8) Guo, S. M.; Xu, P.; Studer, A. Meta-Selective Copper-Catalyzed C−H Arylation of Pyridines and Isoquinolines through Dearomatized Intermediates. *Angew. Chem., Int. Ed.* **2024**, *63*(26), e202405385. https://doi.org/10.1002/anie.202405385.

(9) Jeong, S.; Lee, C.; Joo, J. M. Synergistic Palladium/Silver/Ligand Catalysis for C−H Alkenylation of 2,1,3-Benzofused Heterodiazoles. *Adv. Synth. Catal.* **2025**, *367*(4), e202401139. https://doi.org/10.1002/adsc.202401139.

(10) Frisch, M. J.; Trucks, G. W.; Schlegel, H. B.; Scuseria, G. E.; Robb, M. A.; Cheeseman, J. R.; Scalmani, G.; Barone, V.; Mennucci, G. A.; Petersson, G. A.;

Nakatsuji, H.; Caricato, M.; Hratchian, X. Li. H. P.; Izmaylov, A. F.; Bloino, J.; Zheng, G.; Sonnenberg, J. L.; Hada, M. *Gaussian 09, Revission B.01*, **2009**, Gaussian Inc.

(11) Perdew, J. P.; Wang, Y. Accurate and simple analytic representation of the electron-gas correlation energy. *Phys. Rev. B* **1992**, *45*(23), 13244–13249. https://doi.org/10.1103/PhysRevB.45.13244.

(12) Becke, A. D. Density-functional thermochemistry. III. The role of exact exchange. *J. Chem. Phys. J. Chem. Phys.* **1993**, *98*(7), 5648–5652. https://doi.org/10.1063/1.464913.
